# Place field assembly distribution encodes preferred locations

**DOI:** 10.1371/journal.pbio.2002365

**Published:** 2017-09-12

**Authors:** Omar Mamad, Lars Stumpp, Harold M. McNamara, Charu Ramakrishnan, Karl Deisseroth, Richard B. Reilly, Marian Tsanov

**Affiliations:** 1 Trinity College Institute of Neuroscience, Trinity College Dublin, Dublin, Ireland; 2 School of Psychology, Trinity College Dublin, Dublin, Ireland; 3 Trinity Centre for Bioengineering, Trinity College Dublin, Dublin, Ireland; 4 School of Engineering, Trinity College Dublin, Dublin, Ireland; 5 Department of Physics, Harvard University, Cambridge, Massachusetts, United States of America; 6 Harvard-MIT Division of Health Sciences and Technology, Cambridge, Massachusetts, United States of America; 7 Department of Bioengineering, Stanford University, Stanford, California, United States of America; 8 Department of Neurosurgery, Stanford University, Stanford, California, United States of America; 9 Department of Psychiatry and Behavioral Sciences, Stanford University, Stanford, California, United States of America; 10 Howard Hughes Medical Institute, Stanford University, Stanford, California, United States of America; 11 CNC Program, Stanford University, Stanford, California, United States of America; 12 School of Medicine, Trinity College Dublin, Dublin, Ireland; Institute of Science and Technology Austria, Austria

## Abstract

The hippocampus is the main locus of episodic memory formation and the neurons there encode the spatial map of the environment. Hippocampal place cells represent location, but their role in the learning of preferential location remains unclear. The hippocampus may encode locations independently from the stimuli and events that are associated with these locations. We have discovered a unique population code for the experience-dependent value of the context. The degree of reward-driven navigation preference highly correlates with the spatial distribution of the place fields recorded in the CA1 region of the hippocampus. We show place field clustering towards rewarded locations. Optogenetic manipulation of the ventral tegmental area demonstrates that the experience-dependent place field assembly distribution is directed by tegmental dopaminergic activity. The ability of the place cells to remap parallels the acquisition of reward context. Our findings present key evidence that the hippocampal neurons are not merely mapping the static environment but also store the concurrent context reward value, enabling episodic memory for past experience to support future adaptive behavior.

## Author summary

Episodic memories relate positive or negative experiences to environmental context. The neurophysiological mechanisms of this connection, however, remain unknown. Hippocampal place cells represent location, but it is unclear if they encode only the spatial representation of the environment or if they are also processing information about the reward valence for different locations. Here, we use population analysis to test the hypothesis that the place cells process the dual encoding of spatial representation and experience-dependent reward expectation. We show a unique population code for the experience-dependent value of the context. We present evidence that the accumulation of the place fields mediates the learning of the reward context of the environment. Our data reveal that the causal link between place field distribution and behavioral place preference is mediated by the tegmental dopaminergic activity. Optogenetic control of the ventral tegmental area demonstrates that dopaminergic signaling integrates the encoding of location and reward from hippocampal neurons. These findings shed a new light on the ability of hippocampal neurons to store the experience-dependent context reward value, enabling episodic memory for past experience to support future adaptive behavior.

## Introduction

The hippocampus mediates the formation of adaptive memory for positive or negative experiences [1], but the neurophysiological mechanisms of this learning process remain unknown [2]. The hippocampus may encode locations independently from the stimuli and events that are associated with these locations [3]. Recent findings deduced artificial association between place cells and place preference through the use of optogenetic [4–6] or electrical stimulation [7]. These results provide key evidence linking place cell activity and context-dependent encoding of space [8]. However, it remains unclear if the place cells are simply coincidence detectors or they actively mediate the learning between reward and location. To address this question, we address here 2 possibilities: if place cells don’t integrate information about location and reward, then after global remapping, the distribution of place fields should not be biased towards the location previously associated with reward. Alternatively, if place cells do integrate information about both location and reward, then after global remapping, the distribution of place fields should be precisely biased towards the location previously associated with reward.

One remarkable but underexplored feature of the place cells is their ability to accumulate in locations of the environment that are consistently gainful over repeated exposure. Place fields tend to accumulate near the platform of the water maze, in which the percentage of cells with peak activity around the hidden platform was more than twice the percentage firing in equally large areas elsewhere in the arena [9]. CA1 place fields preferably map locations, such as the escape platform location in an annular water maze [9], selective delivery of water to a single location [10], or the food reward location in a T-maze [11]. The accumulation phenomenon has been described but it never has been validated as a learning mechanism. The biased mapping might simply reflect oversampling of a small number of place cells with no relation to the learning of the task. The place cells from the residual, nonrewarding locations of the environment may simply undergo incomplete field formation due to insufficient path sampling [12, 13]. In this case, remapping of the place cells triggered by the altered spatial navigation approach will dissociate the accumulated place fields from the animal’s preferred location. An alternative proposal is that the accumulation of the place fields is essential for the representation of the reward location. In this case, the scale of accumulation will consistently reflect the degree of place preference, even after scattered allocation of the place fields. We use here a behavioral setup in which, after the learning trials, the place cells undergo global remapping due to the altered spatial navigation approach of the animals during the probe. We designed a protocol to allow for significantly expressed place preference in combination with sufficient path sampling for place field formation in the nonpreferred zone. Previous findings indicated that spatial learning regulates place fields accumulation [14]. Here, we present explicit evidence that the accumulation of place cells is independent population-code mediating the integration of spatial navigation and reward location. We then show that accumulation of place fields is an experience-dependent plastic process, which depends on spatially tuned tegmental dopaminergic activation.

## Results

### Differential navigation in continuous T-maze

**Fig 1:**
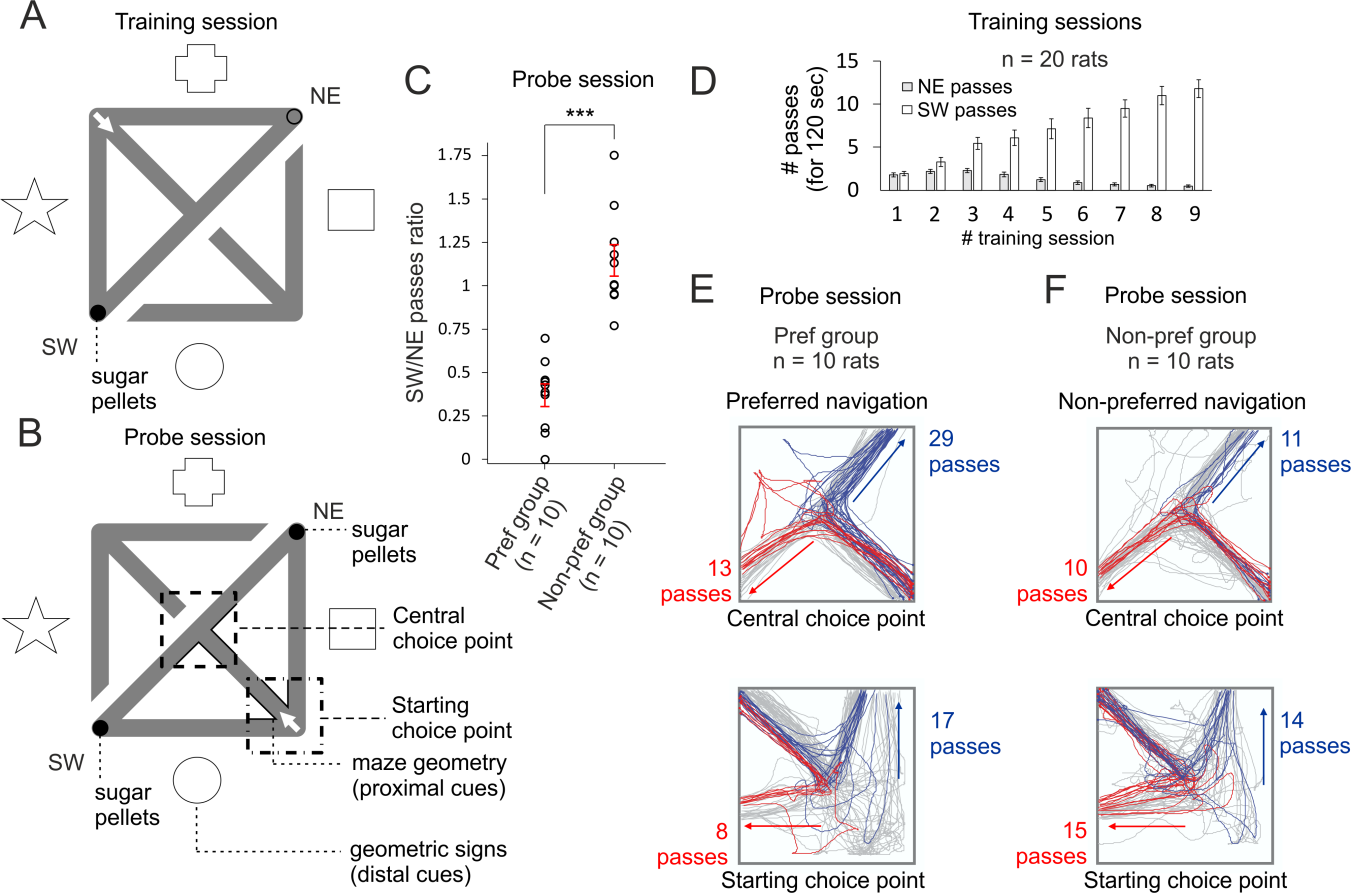
Differential navigation in continuous T-maze. (A) Behavioral setup of the training sessions for the continuous T-maze. The animals were placed on the starting choice point (marked with a white arrow) and allowed to freely navigate for 12 minutes. Two pellets were continuously positioned at the end of the southwest (SW) corner (reward zone, marked with filled black circle), while no pellets were positioned in the northeast (NE) corner. The acquisition of the task was based on the spatial guiding of both maze geometry (proximal cues) and geometric paper signs on the curtains surrounding the arena (distal cues). The identification of the distal cues was designed in a manner in which approximately half of the animals would rely on them for spatial navigation. During the training sessions, the animals were placed on the starting choice point (marked with a white arrow) and allowed to explore the maze. Direct passes between the SW and NE zones were not rewarded with pellets. The opposite section of the maze was visible for the rats but not accessible. The animals (*n* = 20 rats) underwent 9 training sessions over 3 days. (B) During the probe session (on day 4), the animals were exposed to a reversed configuration of the maze, in which access to the training T-maze was disconnected. The animals were placed on the starting choice point of the opposite T-maze (marked with a white arrow), while access to the training T-maze was disconnected. In this way, the rats were exposed to the same proximal but opposite distal cues. Two pellets were constantly positioned in both SW and NE corners. We evaluated the direction of the passes from the starting choice point (marked with a white arrow) and the central choice point. (C) Comparison of the ratio of SW to NE passes. Error bars, mean ± SEM, *n* = 10 rats (preference group), *n* = 10 rats (nonpreference group), 2-tailed independent *t* test, *t*(18) = 6.955, ***p < 0.001. Both groups (the preference and nonpreference groups) had the same training. The chance level of preferential versus nonpreferential navigation was based on the number of passes from the choice points towards each of the corners given the total number of passes; binomial probability values of *p* < 0.05 indicated preferential navigation. (D) Learning curve of the animals’ navigation during the training sessions. All animals underwent 9 training sessions over 3 days. White bars show the number of passes towards the rewarded SW corner for the first 120 seconds of each recording session, while the grey bars show the number of passes towards the NE corner (with no reward) for the first 120 seconds. Two-way ANOVA, between groups, F_(1,19)_ = 59.812, *p* < 0.001, *n* = 20. (E) Representation of the passes from the preference group rat towards the SW corner (in red) and towards NE corner (in blue) from the central choice point (above) and from the starting choice point (below). (F) Representation of the passes from the nonpreference group rat towards the SW corner (in red) and towards NE corner (in blue) from the central choice point (above) and from the starting choice point (below). The grey lines indicate passes from the corners towards the choice points or direct passes between the reward points. *Files dataset is available at Figshare public repository in Tsanov 2016 data / Continuous T-maze folder* *https*:*//figshare*.*com/s/b86a9a111353ba04bd32* *and Tsanov 2017 data / Continuous T-maze CA1 folder* *https*:*//figshare*.*com/s/5c5ba9b2811f3d7b7696*.

To dissociate the place field maps from consistent reward location, we used a continuous T-maze. We trained rats implanted with tetrodes in the CA1 region of the dorsal hippocampus to navigate in a continuous T-maze task, in which the southwest (SW) corner was the constant reward location (Fig 1A). To achieve differential navigation among the rats during the probe, we set the illumination of the recording room to levels at which the animals would rely on both distal and proximal cues (see Materials and methods, Continuous T-maze task). The distal cues represent geometric signs on the curtains around the recording arena, while the proximal cues refer to the maze geometry. All rats (*n* = 20) underwent 9 training sessions for 3 days, during which the animals learned to navigate towards the SW corner (Fig 1D). During the probe session, the rats were placed in the opposite T-maze, with rewards positioned in both corners of the maze (Fig 1B). The rats showed 2 types of navigation strategy (Fig 1C): (1) preference for the northeast (NE) corner passes, which was above chance level (S1A Fig, preference group, *n* = 10), with binomial probability values of *p* < 0.05 (Fig 1E, S1 Table); i.e., navigation predominantly based on proximal cues, and (2) no preference between corners in which the number of passes to each of the corners was below chance level (S1B Fig, nonpreference group, *n* = 10), with binomial probability values of *p* > 0.05 (Fig 1F, S1 Table); i.e., navigation based on opposing proximal and distal cues. Only rats with stable waveforms were allowed to the probe session (S2A–S2D Fig). We recorded 304 hippocampal place cells (*n* = 20 rats) and all of them underwent global remapping in the probe trial (S2E–S2H Fig). From the 241 cells that fired in the reward-associated loop (with navigation towards the SW corner) of the maze during training sessions (reward loop cells), 74.2% (179/241) remapped, while 25.8% (62/241) of place cells did not express place field for the maze configuration of the probe. Concurrently, 63 other place cells expressed place fields in the probe. These place cells were units that either expressed fields in the early training sessions of the nonrewarding loop of the maze or did not express any fields for the training maze configuration (nonreward loop cells). Five out of 179 (2.79%) of the remapped cells kept their location in respect to the maze geometry (mirror representation), while 6/179 (3.35%) kept their location in respect to the distal cues (opposite representation). Our maze setup allows for a combination of place fields global remapping with concurrent preferential navigation. Importantly, the maze design allowed for sufficient path sampling in the nonpreferred section of the maze. We next examined if the remapped place fields accumulated within the preferred navigation of the probe.

### Biased place field configuration after global remapping

**Fig 2:**
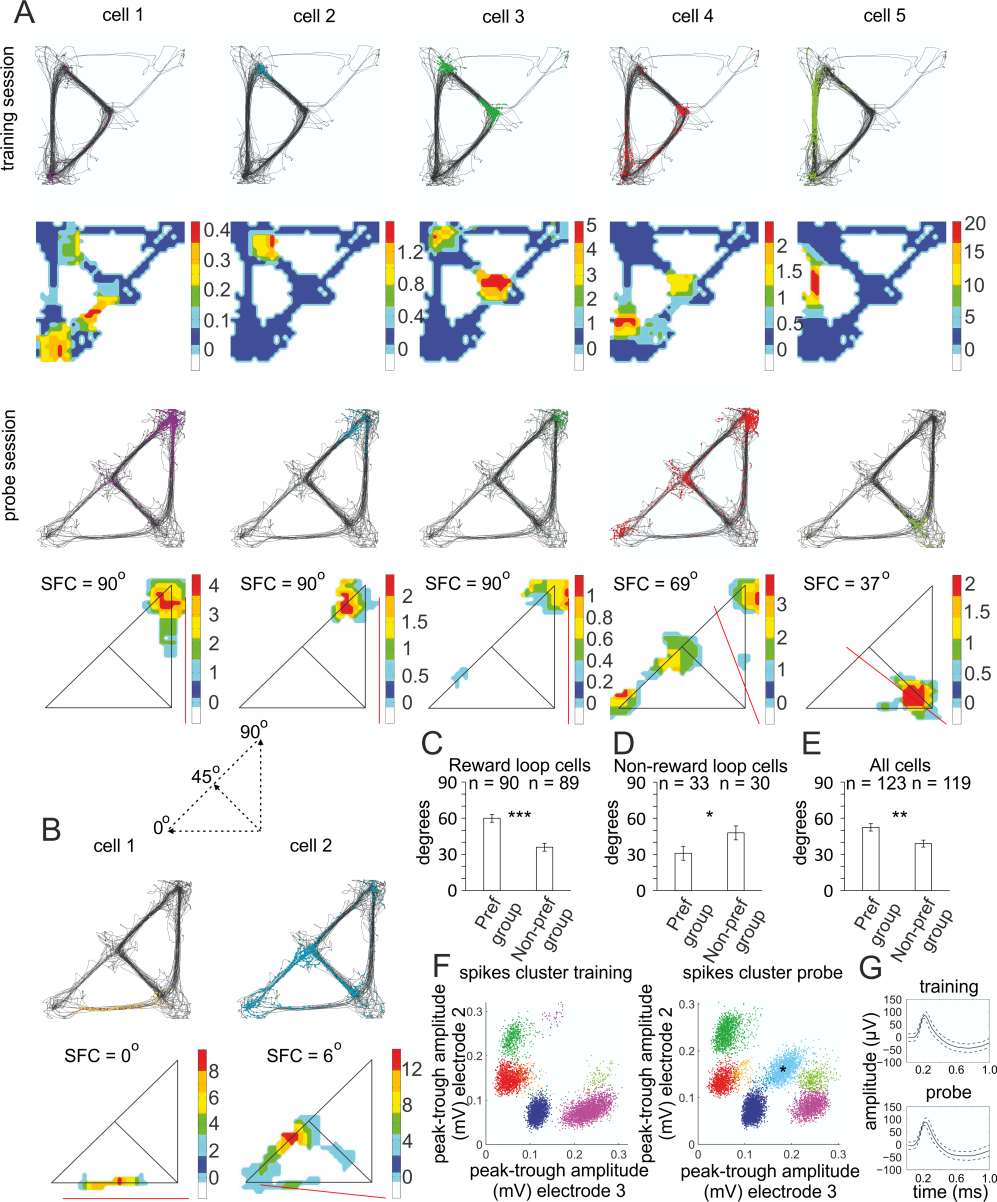
Spatial field configuration (SFC) in the probe session of continuous T-maze. (A) Five sample place cells recorded from the reward loop of the training sessions (reward loop cells) from a representative animal. Upper panels show the animal trajectory with spikes, marked with colored dots (above) and their color-coded firing rate map (below) from the last training session. Lower panels show the animal trajectory with spikes (above) and their place fields’ color-coded firing rate maps (below) from the probe session. The straight red line denotes the direction (in degrees) of the SFC between the southwest (SW) corner at 0° and the northeast (NE) corner at 90°, with midline at 45° (represented at the dashed inset below). (B) Two sample place cells that were absent from the reward loop of the training sessions from the same animal (nonreward loop cells). (C) SFC values from the preference and nonpreference groups are reported for the reward loop cells. Two-tailed independent *t* test, *n* = 90 cells (preference group), *n* = 89 cells (nonpreference group), *t*(177) = 5.189, ***p < 0.001; for (D), the nonreward loop cells, *n* = 33 and *n* = 30, respectively, *t*(61) = −2.112, *p = 0.039; and (E) for all cells, *n* = 123 and *n* = 119, respectively, *t*(240) = 3.210, **p = 0.001. Error bars, mean ± SEM. (F) Sample spike clusters during training session (left) and probe (right). The appearance of spike cluster (blue) of a nonreward loop cell is denoted with a black asterisk. (G) Waveform of a sample reward loop place cell recorded from the last training session (above) and the probe (below). The solid line shows the average waveform shape; the dashed lines show the 1-SD confidence intervals. *Files dataset is available at Figshare public repository in Tsanov 2016 data / Continuous T-maze folder* *https*:*//figshare*.*com/s/b86a9a111353ba04bd32* *and Tsanov 2017 data / Continuous T-maze CA1 folder* *https*:*//figshare*.*com/s/5c5ba9b2811f3d7b7696*.

We investigated whether the configuration of the remapped place fields from the reward loop in the training sessions (reward loop cells) differed between the preference (S3A Fig) and nonpreference groups (S3B Fig). We evaluated the spatial field configuration (SFC) of the individual place fields across both loops with respect to the midline of the maze at 45°, in which the SW corner is 0° and the NE corner is 90°. SFC evaluates the position of the individual place fields across the midline axis (in degrees). The mean SFC of the reward loop cells for the preference group (Fig 2A) was 59.9 ± 3.2°, compared to 35.9 ± 3.2° for the nonpreference group (Fig 2C). The mean SFC of the place fields that did not encode the reward loop in the training sessions (nonreward loop cells) (Fig 2B and 2F) was opposite to the reward loop cells fields with values of 30.9 ± 5.5° and 47.8 ± 5.7° for the preference and nonpreference group, respectively (Fig 2D). The total SFC, including reward and nonreward loop cells, was 52.1 ± 3.0° for the preference and 38.9 ± 2.8° for the nonpreference group (Fig 2E, S2 Table). The duration of the probe (12 minutes) was designed to allow for sufficient sampling of all bins of the maze (for field evaluation, we used a minimum of 9 bins), including sufficient time in the nonpreferred zone for the formation of stable place fields [13].

To confirm the clustering of the cells during the probe for the preference group of rats, we used another analytical approach. We evaluated the location of the center of mass (COM) and its position (spatial angle) in respect to the symmetry axis of the maze (S4A–S4D Fig). The mean COM angle (S4D Fig) of the reward loop cells for the preference group was 56.4 ± 2.5°, compared to 39.6 ± 1.8° for the nonpreference group (S4C Fig). The mean COM angle of the place fields that did not encode the reward loop in the training sessions (nonreward loop cells) was 37.5 ± 3.9° and 49.0 ± 3.8° for the preference and nonpreference groups, respectively, while the total COM angle, including reward and nonreward loop cells, was 51.2 ± 2.2° for the preference and 41.9 ± 1.7° for the nonpreference groups (S4C Fig, S3 Table). These data show that the place preference behavior was accompanied by biased configuration of the reward loop cells towards the preferred NE corner of the maze. The nonreward loop cells counterpoised the SFC bias for both groups.

### Place fields distribution predicts navigation preference

**Fig 3:**
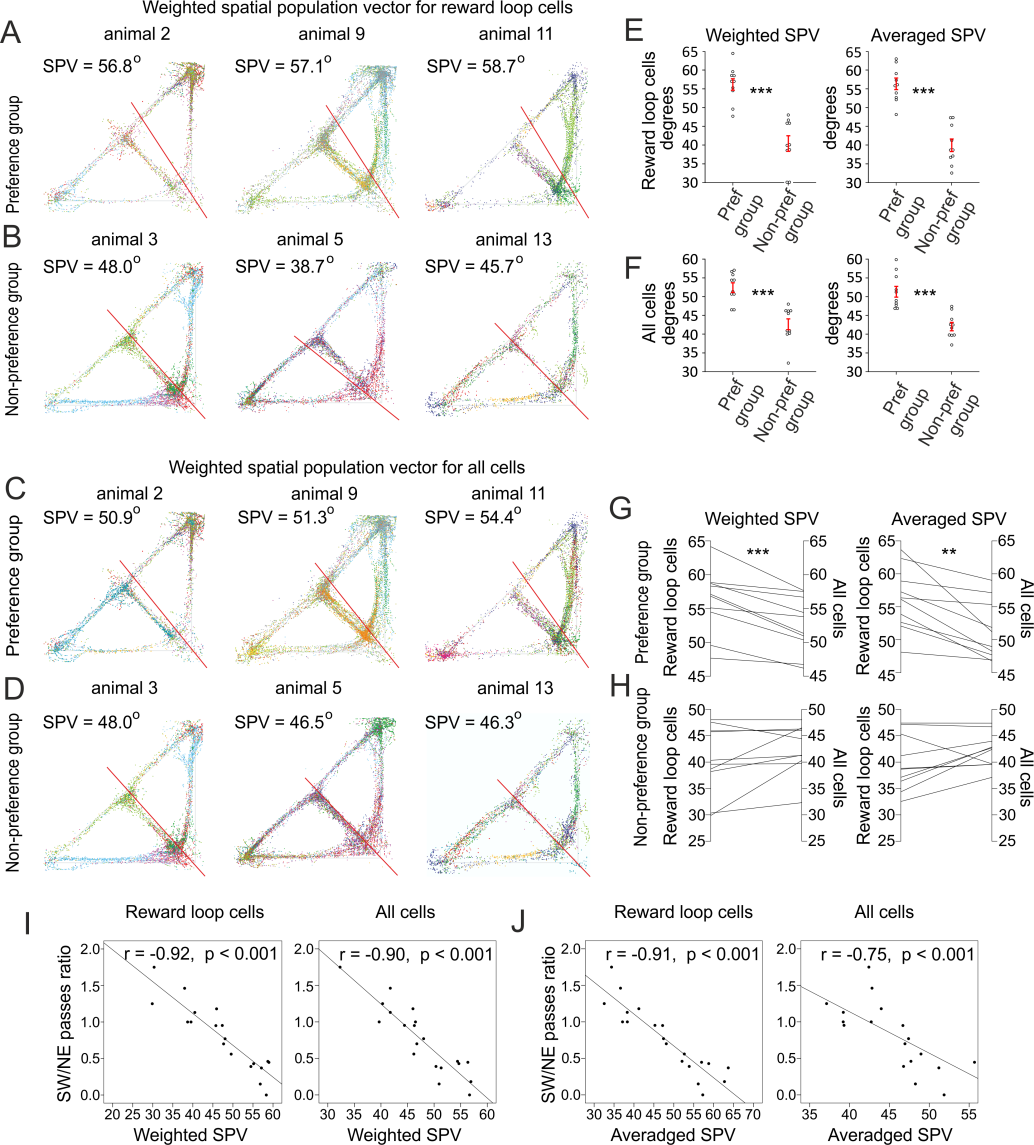
Navigation preference correlates with the place field assembly distribution. (A) Spatial distribution of the spikes (colored dots) from the reward loop cells (represented by different colors) recorded from 3 preference group animals and (B) from 3 nonpreference group animals. The straight red line denotes the weighted spatial population vector (SPV) between the southwest (SW) corner at 0° and the northeast (NE) corner at 90°. (C) Spatial distribution from all cells recorded from the same animals from the preference and (D) nonpreference groups, respectively. (E) Weighted SPV (left) for the preference group (*n* = 10 rats) and the nonpreference group (*n* = 10 rats), 2-tailed independent *t* test, *t*(18) = −6.116, ***p < 0.001; and averaged SPV (right), 2-tailed independent *t* test, *t*(18) = −7.283, ***p < 0.001, for reward loop cells. Error bars, mean ± SEM. (F) Weighted SPV (left), 2-tailed independent *t* test; *t*(18) = −5.048, ***p < 0.001; and averaged SPV (right), 2-tailed independent *t* test, *t*(18) = −5.208, **p = 0.006, for all cells. Error bars, mean ± SEM. (G) Comparison of the weighted SPV (left), *n* = 10, paired *t* test; *t*(9) = 5.043, ***p < 0.001; and averaged SPV (right), *n* = 10, paired *t* test; *t*(9) = 4.331, **p = 0.002 between the reward loop cells and all cells for the preference group. (H) Comparison of the weighted SPV (left), *n* = 10, paired *t* test; *t*(9) = −1.823, *p* = 0.102; and averaged SPV (right), *n* = 10, paired *t* test; *t*(9) = −1.740, *p* = 0.116 between the reward loop cells and all cells for the nonpreference group. (I) Correlation between the SW/NE passes ratio and the weighted SPV of the reward loop cells (left), *n* = 20 rats, Pearson’s *r* = −0.92, *p* < 0.001; and all cells (right), *n* = 20, Pearson’s *r* = −0.90, *p* < 0.001. (J) Correlation between the SW/NE passes ratio and the averaged SPV of the reward loop cells (left), *n* = 20, Pearson’s *r* = −0.91, *p* < 0.001; and all cells (right), *n* = 20, Pearson’s *r* = −0.75, *p* < 0.001. *Files dataset is available at Figshare public repository in Tsanov 2016 data / Continuous T-maze folder* *https*:*//figshare*.*com/s/b86a9a111353ba04bd32* *and Tsanov 2017 data / Continuous T-maze CA1 folder* *https*:*//figshare*.*com/s/5c5ba9b2811f3d7b7696*.

To evaluate whether the spatial distribution of the place field assemblies reflects the navigation preference of each animal, we analyzed the COM from all place cells’ spikes recorded from a single animal using a spatial population vector (SPV). This parameter estimates the Cartesian distribution of the spikes from multiple place fields. The SPV is based on place field rates, which represent spiking as a function of the occupancy for each pixel (see Materials and methods, SPV). Therefore, the SPV is not biased by the time spent in a particular section of the maze. The SPV values are measured also between the SW corner, where SPV is 0°, and the NE corner, where SPV is 90°. Values below 45° indicate that the place fields distributed preferably towards the SW corner of the maze, while values of above 45° indicate NE distribution preference. We computed both weighted SPV (in which the cells are weighted by their firing rate) and averaged SPV (in which all cells are weighted equally). The reward loop cells from the preference group (Fig 3A and 3E, S5A Fig) showed an uneven distribution of their spikes in favor of the NE corner, with weighted SPV of 56.1 ± 1.6° and averaged SPV of 56.2 ± 1.5°. Concurrently, the place cells from the nonpreference group (Fig 3B and 3E, S5B Fig) expressed values of 40.3 ± 2.0° for weighted SPV and 39.8 ± 1.6° for averaged SPV (S4 Table). The addition of the nonreward loop cells shifted the SPV values towards the midline of 45° (Fig 3F). The SPV values decreased for the place cells from the preference group (Fig 3C) to 52.4 ± 1.2° and 51.3 ± 1.4° weighted and averaged SPV, respectively (Fig 3G, S5C Fig), whereas the SPV values for the nonpreference group (Fig 3D) increased to 42.7 ± 1.4° and 42.0 ± 1.1°, respectively (Fig 3H, S5D Fig). The close link between place preference and place field assembly distribution is best represented by the correlation between the animals’ navigation and the SPV. The degree of place preference, expressed by the SW/NE passes ratio, showed strong correlation with the SPV values (Pearson’s *r* = −0.92, Fig 3I left). This correlation was not affected by the presence of the nonreward loop cells for the weighted SPV (Pearson’s *r* = −0.90, Fig 3I right). However, the averaged SPV correlation was greater for the reward loop cells (Pearson’s *r* = −0.91, Fig 3J left) compared to all cells (Pearson’s *r* = −0.75, Fig 3J right). Thus, the firing rate might complement the distribution of the place cells for preferred location. To demonstrate that the correlation of the SPV and animals’ navigation is not affected by the analytical design, we forced biased navigation to the south section of the maze during the probe (S6A Fig). Despite the high SW/NE passes ratio and the predominant timing in the SW corner (S6B Fig), the SPV was directing towards the opposite NE corner (S6C Fig). These findings provide key evidence that accumulation of place fields persists after global remapping, and the scale of place field assembly distribution precisely reflects the degree of place preference.

The ventral tegmental area (VTA) is a central structure in the propagation of reward signals [15, 16]. We recorded the activity of slow-spiking neurons (with firing rate of <10 Hz, i.e., the rate diapason of dopaminergic neurons) from VTA and measured their firing rates for the choice points of the probe exploration (S7A Fig). Cue-evoked activity in tegmental dopaminergic neurons reflects the value of the predicted rewards [17, 18]. We evaluated separately the firing rate for the passes towards the NE corner and towards the SW corner (S7B Fig). To evaluate the dissimilarity of the firing rate in both directions, we divided the firing rate for the SW passes over the firing rate for the NE passes (SW/NE passes firing rate ratio). The average ratio values for animals with preferred navigation (*n* = 3 rats, 16 cells) was 0.58 ± 0.04, compared to 1.01 ± 0.03 for animals with preferred navigation (*n* = 4 rats, 14 cells) (S7C Fig). The significantly lower ratio for the preference group animals (S7D Fig) indicates that the tegmental slow-spiking neurons spike with higher rate when the animals from this group are navigating towards their preferred section of the maze (S7E Fig). These data propose that the dopaminergic signaling might mediate the navigation-related bias of the place fields’ distribution.

### Suppression of VTA dopaminergic neurons evokes navigation preference

**Fig 4:**
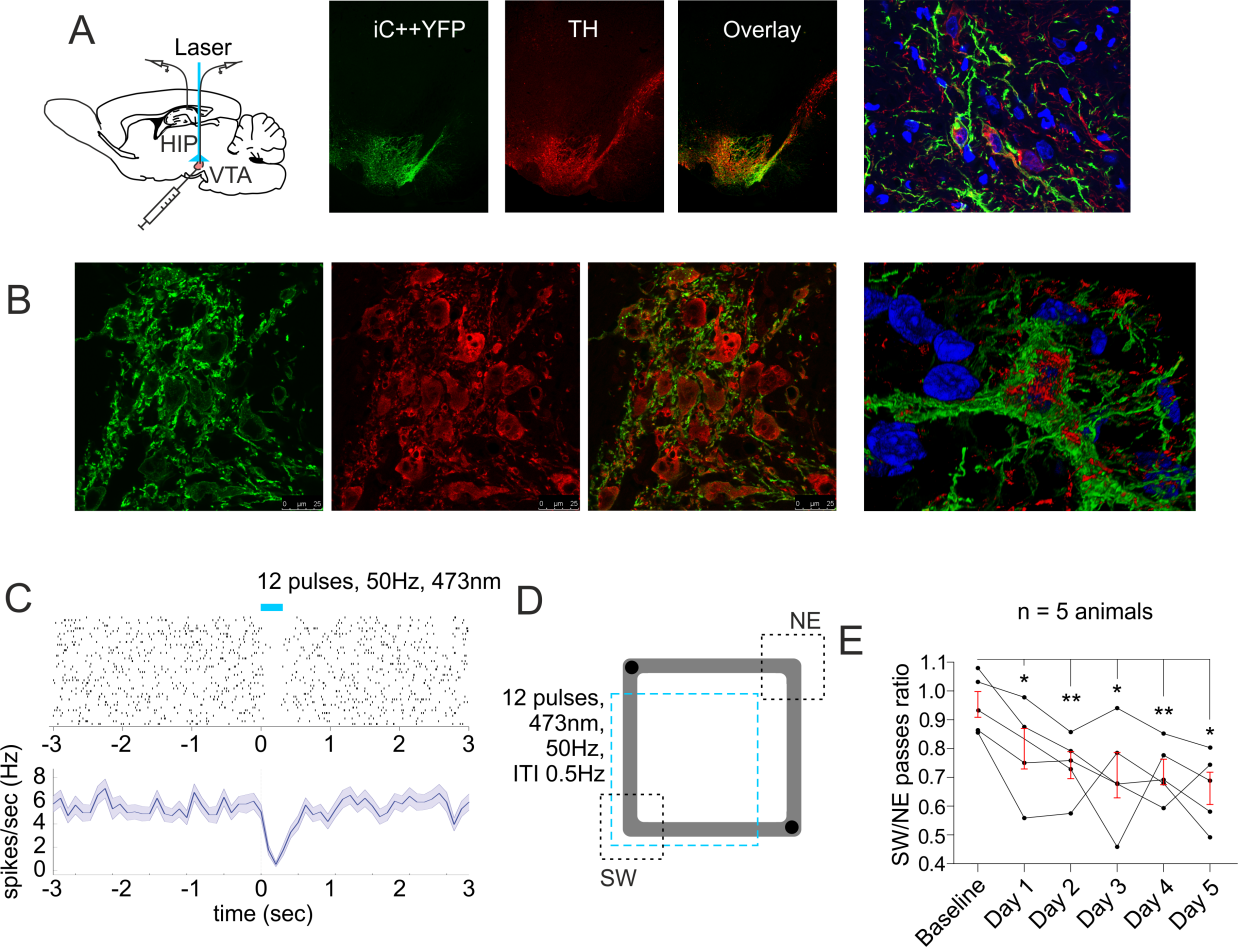
Optogenetic suppression of ventral tegmental area (VTA) dopaminergic neurons evokes place avoidance. (A) Atlas schematic shows chronically implanted animals with optic fiber and tetrodes in the VTA or hippocampal CA1 region. Middle panels: yellow fluorescent protein (YFP) expression, tyrosine hydroxylase (TH) staining, and their overlay in VTA of TH::Cre rats injected with Cre-inducible light-activated chloride channel (iC++) adeno-associated virus. The right image shows a confocal image of the VTA with YFP, TH, and DAPI overlaid. (B) High-magnification confocal images of YFP expression and TH staining and their colocalization in the VTA. Right image shows a confocal 3D render of the VTA neuron with YFP, TH, and DAPI overlay. (C) Raster plot from 40 repetitions (above) and firing frequency (below) of optically evoked time-locked inhibition of a VTA slow-spiking cell. Time 0 indicates the delivery of the first train of the stimulation protocol. (D) Behavioral setup of laser application (marked with a blue dashed square) in the south and west arms of the rectangular-shaped linear track. Black circles indicate pellets delivery. The ratio of the passes through the southwest (SW) and northeast (NE) corners (marked with black dashed squares) was compared between control and photoinhibition sessions. (E) Light-induced place avoidance throughout 5 consecutive days, *n* = 5 rats, paired *t* test, day 1, *t*(4) = 3.545, *p = 0.024; day 2, *t*(4) = 6.146, **p = 0.004; day 3, *t*(4) = 2.978, *p = 0.041; day 4, *t*(4) = 5.423, **p = 0.006; day 5, *t*(4) = 4.501, *p = 0.011. Error bars, mean ± SEM. *Files dataset is available at Figshare public repository in Tsanov 2016 data / iC++ electrophysiology and iC++ immunohistology folders* *https*:*//figshare*.*com/s/b86a9a111353ba04bd32*.

We next aimed to induce place preference behavior without altering the spatial navigation approach or reward location, but by suppressing the reward signals in the brain [19]. VTA dopaminergic suppression is known to evoke place avoidance [15]. Our goal was to test if dopaminergic signaling mediates the integration of location and reward encoding from hippocampal neurons. For inhibition of the VTA tyrosine hydroxylase positive (TH+) neurons, we injected a Cre-inducible viral construct, adeno-associated virus AAV-EF1a-DIO-iC++-YFP, expressing light-activated chloride channels (iC++) [20], in the TH::Cre rat line (Fig 4A). 90 ± 2% of neurons that expressed yellow fluorescent protein (YFP) also expressed TH, while 52 ± 8% of neurons that expressed TH also expressed YFP (*n* = 5 rats, *n* = 1,116 TH cells, *n* = 665 YFP cells; *n* = 590 TH-YFP cells; Fig 4B, S8A–S8C Fig). Local delivery of blue light (473 nm) suppressed the spiking of neurons infected with AAV-EF1a-DIO-iC++ (Fig 4C). Of these cells, 90.9% (30/33) spiked with baseline frequency below 10 Hz, with average frequency of 4.7 ± 2.6 Hz. Firing rate of <10 Hz is an electrophysiological characteristic of VTA dopaminergic neurons [21]. The application of blue light triggered inhibition in 38% (30/78) of the recorded slow-spiking neurons (S9A–S9C Fig) and 5.5% (3/55) of the fast-spiking cells. To confirm that injection of AAV-iC++ mostly affected the TH+ neurons, we tested if photoinhibition would trigger place avoidance, which is a behavioral correlate of dopaminergic suppression [15]. We used a rectangular-shaped linear track because the navigation of the animals during the baseline recordings was the most evenly distributed between the opposite corners when compared to other tracks. Light delivery to VTA in the SW area of the track (Fig 4D) resulted in gradual avoidance of this section, with a decrease of SW/NE passes ratio to 0.79 ± 0.07 of total passes after the first and 0.74 ± 0.05 after the second session, compared to the baseline ratio of 0.95 ± 0.05 (*n* = 5 rats, Fig 4E).

### The spatial distribution of place fields depends on tegmental dopaminergic activity

**Fig 5:**
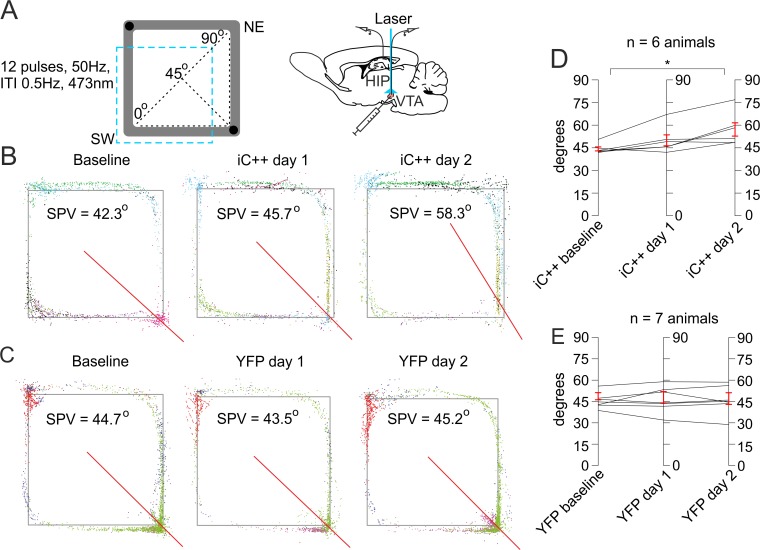
Suppression of tegmental dopaminergic activity induces bias in the place field assembly distribution. (A) Behavioral setup of laser application (marked with a blue dashed square) in the south and west arms of rectangular-shaped linear track. Black circles indicate pellets delivery. The spatial population vector (SPV) values are measured between the southwest (SW) corner, where the SPV is 0°, and the northeast (NE) corner, where SPV is 90°. The diagonal between the SW and NE corners indicates SPV of 45°. Atlas schematic on the right shows the location of the optic fiber in the ventral tegmental area (VTA) and recording tetrodes in the hippocampal CA1 region. (B) Sample spatial distribution of the spikes (colored dots) from the place cells (represented by different colors) recorded during the baseline session (left), first (middle) and second (right) light delivery session from a light-activated chloride channel (iC++) group animal and (C) from a control yellow fluorescent protein (YFP) group animal. The straight red line denotes the weighted SPV in degrees between SW at 0° and NE at 90°. (D) Comparison of the weighted SPV in degrees between the baseline (left), first session (middle) and second session (right) photoinhibition for the iC++ group of rats, *n* = 6 rats, paired *t* test, day 1: *t*(5) = −2.169, *p* = 0.082; day 2: *t*(5) = −3.784, *p = 0.013; and (E) control light application for the YFP group of rats, *n* = 7, paired *t* test, day 1: *t*(6) = 0.136, *p* = 0.896; day 2: *t*(6) = 0.564, *p* = 0.593. Error bars, mean ± SEM. *Files dataset is available at Figshare public repository in Tsanov 2016 data / Rectangular track folder* *https*:*//figshare*.*com/s/b86a9a111353ba04bd32*.

To test the hypothesis that reward signals guide the spatial distribution of place field assemblies, we analyzed the effect of photoinhibition on the hippocampal place field assembly distribution using the SPV of place cells taken from ensemble recordings in the rectangular-shaped linear track (Fig 5A). The weighted SPV for the YFP-iC++ group of rats (*n* = 6 rats, 51 place cells) shifted from 44.3 ± 1.3° to 50.0 ± 3.6° (Fig 5B) and 57.2 ± 4.4° after the first and the second photoinhibition session, respectively (Fig 5D, S5 Table). The observed effect was a consequence of partial place and rate remapping (S10A Fig). The changes in the weighted SPV and SW/NE passes ratio were significantly correlated (Pearson’s *r* = −0.24, *p* = 0.045). No significant change was evident for the SPV of the control YFP group of animals (*n* = 7 rats, 63 place cells, Fig 5C, S10B Fig) between the baseline 45.4 ± 2.0° and the light delivery sessions (46.4 ± 3.3° and 46.1 ± 3.6°; Fig 5E; S6 Table). Similarly, the averaged SPV for the YFP-iC++ group shifted from 44.6 ± 1.4° to 50.5 ± 2.8° and 52.4 ± 4.5° after the first and second photoinhibition sessions, respectively (S11A Fig). Forced biased navigation towards the NE corner with concurrent photoinhibition in the NE quadrant resulted in SPV with value opposed to the photoinhibition zone (42.2°), showing that SPV is not affected by the path sampling (S12 Fig). These results provide evidence that the dopaminergic signals regulate the place fields’ assembly distribution, which is accompanied behaviorally by navigation preference.

### Dopaminergic projections mediate augmentation of place cells’ firing rate

**Fig 6:**
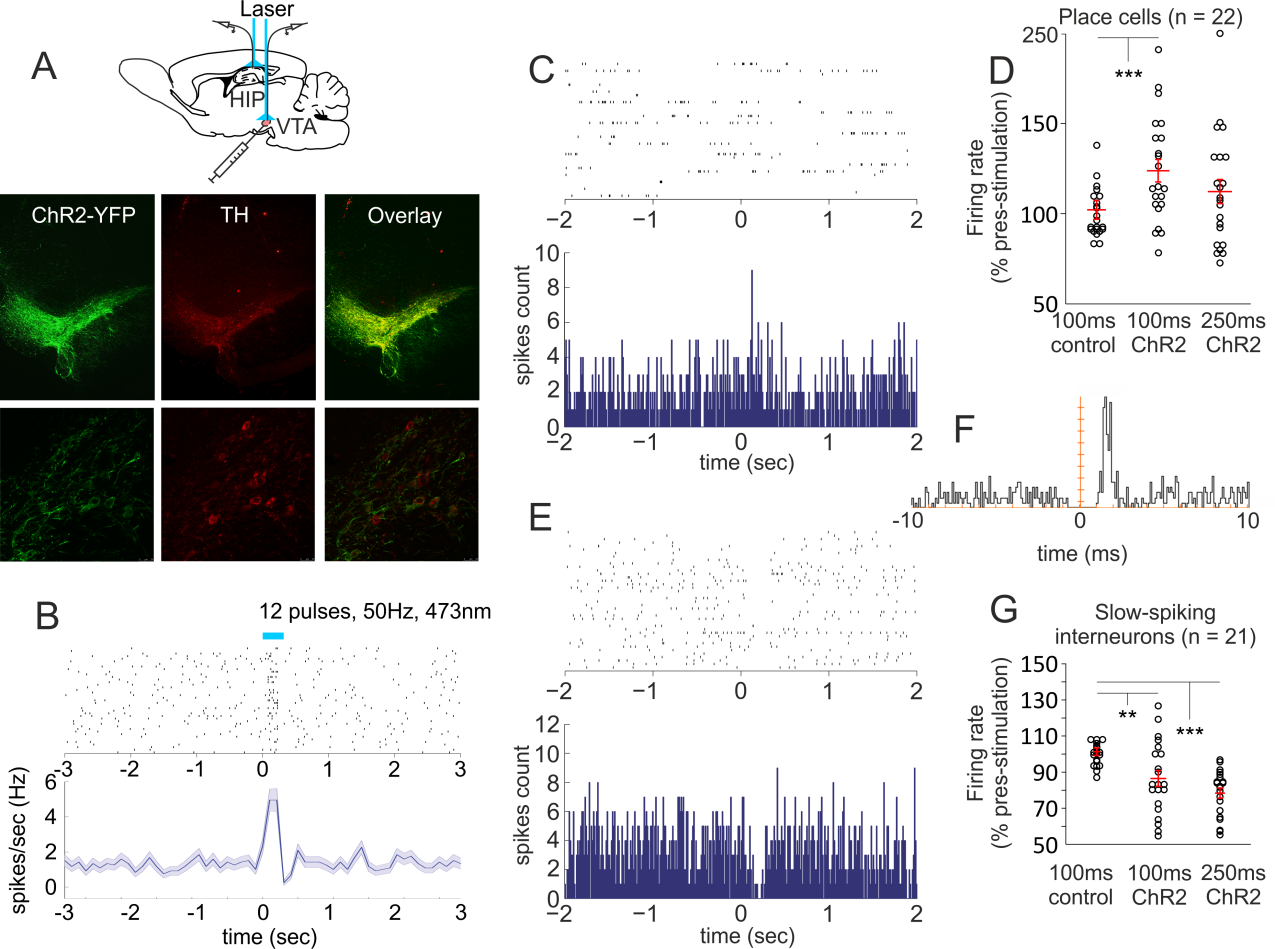
Optogenetic excitation of ventral tegmental area (VTA) dopaminergic neurons augments place cells’ spiking. (A) Atlas schematic shows optic fiber and tetrodes in the VTA or hippocampal CA1. Images below show channelrhodopsin 2 with yellow fluorescent protein (ChR2-YFP) expression, tyrosine hydroxylase (TH) staining, and their overlay in VTA of TH::Cre rats injected with E123T/T159C virus. (B) Raster plot from 40 repetitions (above) and firing frequency (below) of optically evoked time-locked excitation of a VTA slow-spiking cell. (C) Raster plot from 40 repetitions (above) and spike count of 120 repetitions (below) of a sample place cell. Time 0 indicates the onset of the stimulation protocol. (D) Firing rate of 22 place cells 100 ms after the onset of the stimulation protocol expressed as a percentage of the prestimulation values, for control (left bar) and photostimulation (middle bar), paired *t* test: *t*(21) = −3.910, ***p < 0.001; the right bar shows the firing rate (% prestimulation) for 250 ms after photostimulation onset, paired *t* test: *t*(21) = −1.767, *p* = 0.092. Error bars, mean ± SEM. (E) Raster plot and spike count of a slow-spiking interneuron. (F) Spiking cross correlogram between the place cell and the interneuron shown in (C) and (E), respectively. (G) Firing rate of 21 interneurons 100 ms after photostimulation (% prestimulation), for control (left bar) and photostimulation, paired *t* test: *t*(20) = 3.002, **p = 0.007. The right bar shows the firing rate for 250 ms after photostimulation onset, paired *t* test: *t*(20) = 6.841, ***p < 0.001. Error bars, mean ± SEM. *Files dataset is available at Figshare public repository in Tsanov 2016 data / E123T electrophysiology and E123T immunohistology folders* *https*:*//figshare*.*com/s/b86a9a111353ba04bd32*.

To examine how VTA projections affect hippocampal neuronal spiking, we implanted rats with an optical fiber and recording tetrodes in the pyramidal layer of dorsal hippocampal CA1 area and injected Cre-dependent AAV, which mediates blue light–induced depolarization of the dopaminergic neurons in TH::Cre rats [22]. The injection of AAV5-EF1a-DIO-ChR2-E123T/T159C resulted in specific expression of light-activated channelrhodopsin 2 (ChR2) tagged with a fluorescent protein in TH+ neurons (Fig 6A). Blue light delivery entrained the firing of slow-spiking neurons in the lateral VTA (Fig 6B). We evaluated the effect of the light delivery on the spiking of hippocampal place cells during a pellet-chasing task in open arena. The photostimulation (473 nm, 50 Hz, 12 pulses, 5 ms pulse duration) was applied every 6 seconds, including intrafield and extrafield passes. We investigated the firing frequency of 22 place cells from 3 rats during the photostimulation protocol with a duration of 250 ms as well as the neuronal firing in the first 100 ms after the protocol onset (Fig 6C). The spiking of the place cells increased to 123.8 ± 6.3% of the prestimulation firing rate for the first 100 ms and 112.3 ± 6.7% for the entire protocol of 250 ms (Fig 6D, S7 Table). The photostimulation effect was mediated by the intrafield spike rate increase, whereas the light delivery did not affect the number of extrafield spikes (S13A–S13C Fig, S8 Table). Concurrently, the photostimulation reduced the firing rate of 21 slow-spiking interneurons (cells with firing rate <10 Hz, Fig 6E) to 86.5 ± 4.4% for the first 100 ms and 78.4 ± 2.7% for 250 ms (Fig 6G, S7 Table). We identified a functional connection between the slow-spiking interneurons and the place cells (Fig 6F, S14A–S14C Fig). The spike cross correlation indicates a monosynaptic connection between cell pairs [23, 24]. The position of the cross correlation peak in relation to time 0 indicated that the place cells in our recordings were presynaptic, while the slow-spiking interneurons were the postsynaptic neuron of each pair. These data show that dopamine signal enhances the excitability of the hippocampal place cells, whereas for a subset of postsynaptic slow-spiking interneurons, dopaminergic signaling gradually reduces their ability to trigger spikes.

### Spatial activation of TH+ projections determines the direction of place field remapping

**Fig 7:**
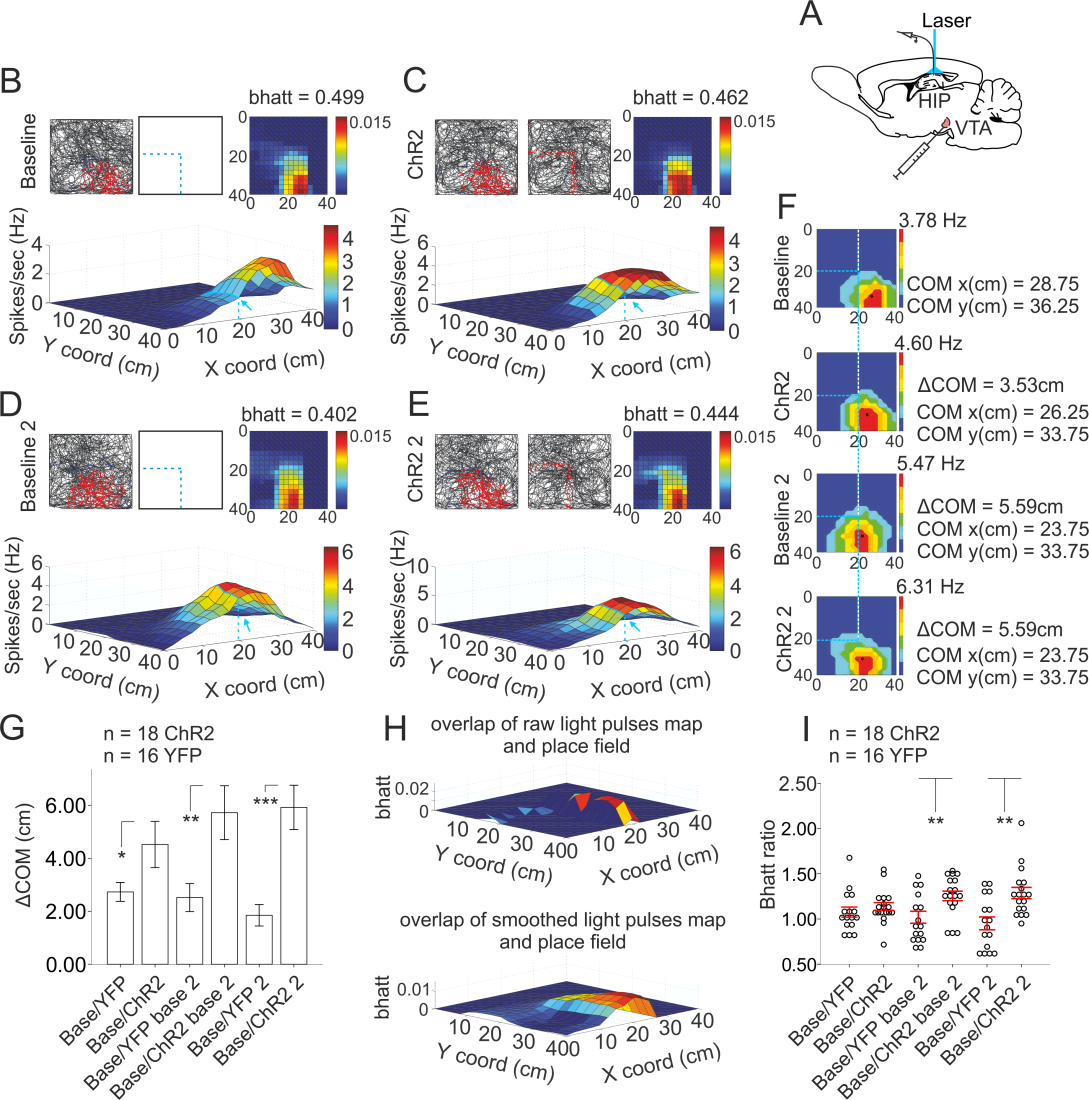
Dopamine signal directs the shift of place field center of mass (COM). (A) Atlas schematic shows E123T/T159C virus injection in the ventral tegmental area (VTA) of TH::Cre rats with optic fiber and tetrodes implanted in the hippocampal CA1. (B) Baseline recording of a sample place cell. Top left panel represents the animal trajectory with spikes (red dots), top middle panel shows the coordinates of the subsequent laser application (blue dashed lines), and top right panel shows color-coded Bhattacharyya distance metric (bhatt) overlap between the distribution of spikes and the applied light pulses from the subsequent photostimulation session (lower bhatt values mean higher overlap of both distributions). Bottom image shows 3D color-coded firing rate map. Blue line superimposed on the 3D firing map indicates where the laser light was applied. The x-coordinate is indicated with a blue arrow. Note that the overlapping of the 3D place field and the blue line and arrow is least evident during the first baseline recording. (C) First photostimulation session of the same place cell (channelrhodopsin 2 [ChR2]). Top middle panel shows the location of the applied laser light pulses (red dots). (D) Second baseline (baseline 2) and (E) second photostimulation (ChR2 2). (F) 2D color-coded rate maps for the same recordings (B-E). Comparison of the COM (marked with black circle) between the baseline (top), first photostimulation, second baseline, and second photostimulation (bottom). ΔCOM is measured by the change of COM location at the x-y coordinate system. COM and ΔCOM values (cm) are shown for each session on the right. Blue dashed lines indicate the laser application. Note the increasing proximity between the COM and the vertical dashed line. (G) ΔCOM for control (yellow fluorescent protein [YFP], *n* = 16 cells) and ChR2 (*n* = 18 cells) groups between the baseline and the first photostimulation, 2-tailed independent *t* test, *t*(32) = 2.101, *p = 0.030; second baseline (base 2), *t*(32) = 3.042, **p = 0.009; second photostimulation, *t*(32) = 4.184, ***p < 0.001. Error bars, mean ± SEM; **p < 0.01. (H) 3D color-coded raw map (above) and smoothed rate map (below) of the overlap between the delivered light pulses and the place field from the second baseline recording. High overlap is represented by red colors while low overlap is in blue. (I) Ratio of bhatt values of the baseline relative to the first photostimulation session; second baseline, 2-tailed independent *t* test, *t*(32) = 2.803, **p = 0.009; second photostimulation session, *t*(32) = 2.863, **p = 0.007 for YFP (*n* = 16) and ChR2 (*n* = 18) groups. Error bars, mean ± SEM. *Files dataset is available at Figshare public repository in Tsanov 2016 data addition folder* *https*:*//figshare*.*com/s/b2c6e7a8a0417820720c*.

To evaluate the causality of VTA activation on the direction of place field center remapping, we applied photostimulation tangential to the place fields recorded in the open field arena during a pellet-chasing task. With the open arena, we have eliminated the goal-directed [11] and directional place field plasticity [25] occurring in linear tracks with prospective reward location. We photostimulated the dopaminergic fibers in hippocampal CA1 of TH-Cre rats injected in VTA with AVV-ChR2-YFP (Fig 7A) and evaluated the field properties of hippocampal place cells (S1 Data). We evaluated the distribution of place cell spikes across the subsequent recordings: baseline (Fig 7B), first photostimulation (Fig 7C), second baseline (Fig 7D), and second photostimulation (Fig 7E). We estimated if there is a shift in the place fields’ COM and measured the distance (Fig 7F). We compared the field properties of place cells of the TH-Cre rats injected with AVV-ChR2-YFP (ChR2 group) to the cells from animals injected with control viral vector (YFP group, S15A and S15B Fig, S2 Data). We observed a gradual shift increase of the COM (ΔCOM) between the recording sessions (Fig 7G) for cells (*n* = 18) from animals injected with AVV-ChR2-YFP (ChR2, *n* = 4 rats) but not for cells (*n* = 16) from control rats (YFP, *n* = 3 rats) injected with AVV-YFP. To determine if the place field shift is directed towards the location of the applied light pulses, we used a specific measure (i.e., Bhattacharyya distance metric [bhatt], Fig 7H). Bhattacharyya distance quantifies the distance between the distribution of the place cell spikes and the distribution of the light pulses, which is constant (lower bhatt values mean higher overlap of both distributions). The bhatt value in our experiments was gradually reduced after the first photostimulation, second baseline, and second photostimulation only for the cells from the ChR2 group of rats (Fig 7I, in which the ratio of baseline over ChR2 increased) but not for the cells from control rats (YFP group, Fig 7I). There was no significant change in the peak and mean place field rate or in the spatial coherence of the place field (S16A Fig). A transient increase of the field size paralleled the remapping process (S16B Fig). These data show that photostimulation of the dopaminergic fibers evoked field plasticity. Furthermore, field ΔCOM shifted towards the stimulus location.

### Spatial activation of VTA guides place field plasticity

**Fig 8:**
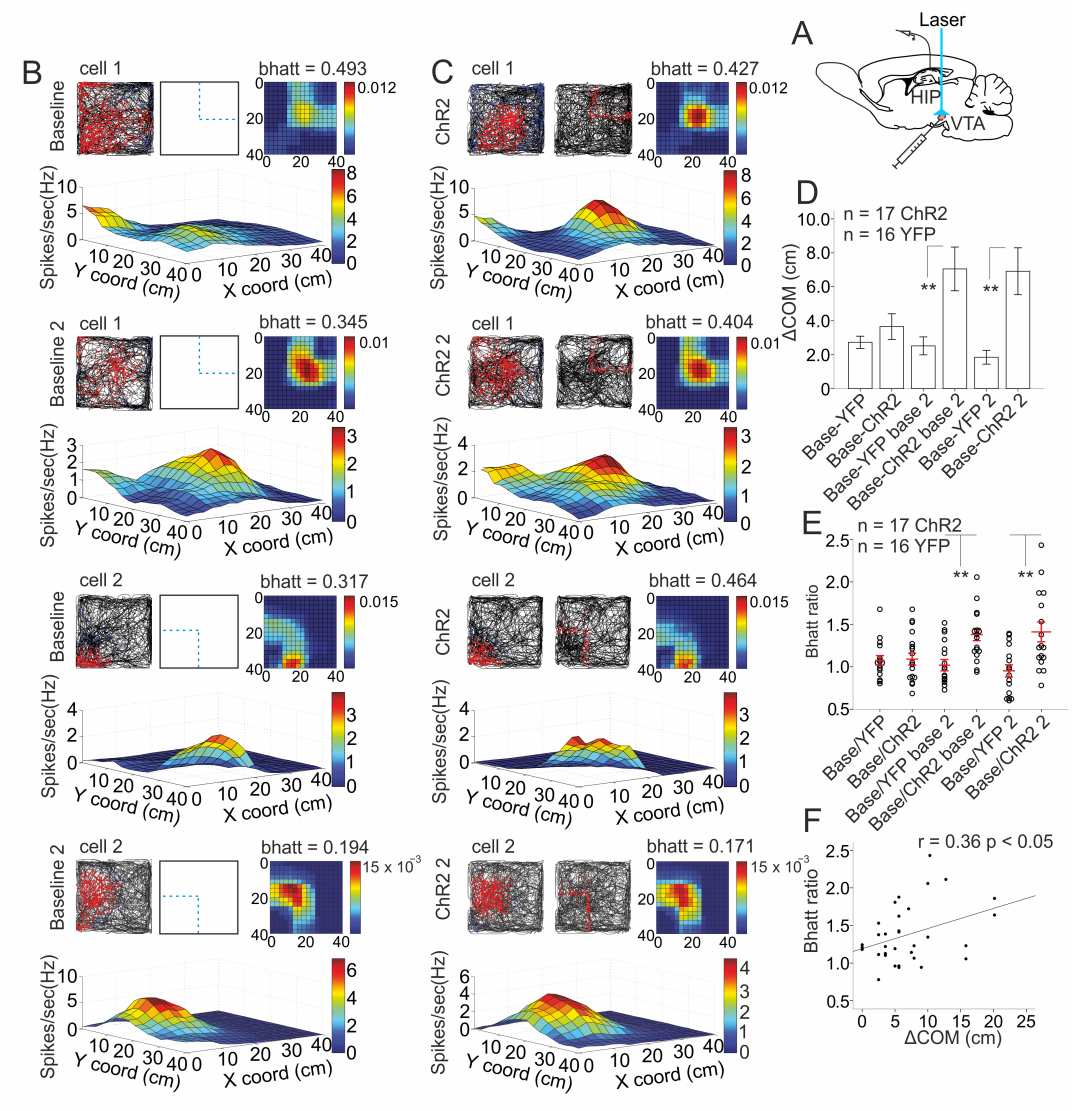
Ventral tegmental area (VTA) activation induces the shift of place field center of mass (COM). (A) Atlas schematic shows E123T/T159C virus injection and optic fiber implantation in the VTA of TH::Cre rats and tetrodes position in the hippocampal CA1. (B) Baseline recordings of 2 sample place cells. The upper group of panels shows the first baseline, while the lower group of panels shows the second baseline. Top left panels represent the animal trajectory with spikes (red dots), top middle panels show the coordinates of the laser application (blue dashed lines), and top right panels show color-coded Bhattacharyya distance metric (bhatt) overlap between the distribution of spikes and the applied light pulses from the subsequent photostimulation session (lower bhatt values mean higher overlap of both distributions). Bottom images show 3D color-coded firing rate maps. (C) First (channelrhodopsin 2 [ChR2], upper panel groups) and second (ChR2 2, lower panel groups) photostimulation sessions of the same place cells. Top middle panels show the location of the applied laser light pulses (red dots). (D) Center of mass shift (ΔCOM) between the baseline and the first photostimulation; second baseline (base 2), 2-tailed independent *t* test, *t*(31) = 3.291, **p = 0.002; second photostimulation, *t*(31) = 3.449, **p = 0.002 for control group (yellow fluorescent protein [YFP], *n* = 16 cells) and ChR2 group (*n* = 17 cells). Error bars, mean ± SEM; **p < 0.01. (E) Ratio of bhatt values of the baseline relative to the first photostimulation session; second baseline, 2-tailed independent *t* test, *t*(31) = 3.423, **p = 0.002; second photostimulation session, *t* test, *t*(31) = 3.289, **p = 0.003 for YFP (*n* = 16) and ChR2 (*n* = 17) groups. Error bars, mean ± SEM. (F) Correlation between bhatt and ΔCOM for the ChR2 group, *n* = 34, Pearson’s *r* = 0.360, *p* = 0.036. *Files dataset is available at Figshare public repository in Tsanov 2016 data / Open arena spatial stimulation folder* *https*:*//figshare*.*com/s/b86a9a111353ba04bd32*.

We next implanted optic fiber for light delivery in VTA to evaluate if the sparse TH+ projections evoke distributed place field plasticity across the hippocampal network (Fig 8A). The second baseline recording showed that the ChR2 group (Fig 8B) of cells shifted their COM (ΔCOM) 24 hours after the first photostimulation session (6.96 ± 1.21 cm, *n* = 3 rats, 17 cells, Fig 8C and 8D), whereas ΔCOM for the control YFP group was smaller (2.51 ± 0.5 cm, *n* = 3 rats, 16 cells, Fig 8D). The distribution of overlap between the photostimulated field and the place field measured by bhatt increased only for the ChR2 group (S3 Data) but not for the YFP controls (Fig 8E). The increased overlap indicates that the direction of ΔCOM shift was tuned towards the photostimulation coordinates only for the ChR2 group. The correlation between ΔCOM and Bhattacharyya distance was significant for the ChR2 (Pearson’s *r* = 0.36, *p* = 0.036, Fig 8F) but not for the YFP group (Pearson’s *r* = 0.05, *p* = 0.784). We also tested an alternative method for the activation of VTA projections to the hippocampal formation. Stimulation of the medial forebrain bundle (MFB) (S17 Fig), which contains dopaminergic projections from VTA, was expected to exert a similar effect on the place field plasticity. We applied nonselective electrical MFB stimulation (S18 Fig, S4 Data) and confirmed a significant correlation between ΔCOM and Bhattacharyya distance (Pearson’s *r* = 0.37, *p* = 0.024, S19A Fig). The increase of ΔCOM was significant after the second MFB stimulation session (4.72 ± 2.94 cm, *n* = 5 rats, 18 cells, S19B Fig) compared to the YFP controls. Together, these results indicate that place cells remap their fields in a direction determined by repeatedly augmented VTA activity.

## Discussion

In this study, we demonstrated that the spatial distribution of hippocampal place cells correlates to the degree of navigation preference. The population code for reward location was evident after global remapping. The assembly distribution bias of the reward loop cells precisely reflected the maze geometry previously associated with reward. The dopaminergic VTA fibers exerted activity-dependent control on the spatial distribution of the place field assemblies. Finally, the spatial location of the dopamine signal denoted the remapping direction of the place field’s COM. Our data indicate that the ability of place cells to encode episodic memories relies on dopamine-dependent place field plasticity.

### Evaluation of place field parameters in nonpreferred locations

The spatial environment can be associated with positive or negative context and repetitive exposure to such environment drives goal-directed behavior [26, 27]. The spatial memory in rodents is behaviorally measured by their navigation in tasks where the rodents learn to associate reward or aversion with a particular location in the environment [21]. The resulting place preference is evaluated either by the path of the animals towards the preferred destination or by the time spent there [15, 16]. One of the main challenges when investigating place cell activity during place preference tasks is the insufficient path or time spent in the nonpreferred location. Insufficient path sampling impedes the formation and evaluation of the place fields [12]. To achieve both place preference and sufficient path sampling in the nonpreferred location, we used a continuous version of the cross-maze task [28]. This experimental design allowed us to evaluate the behavior of the animals at both choice points of the maze. The continuous T-maze protocol did not induce differences in the task demands, and the consistency of the reward was independent of the animals’ behavior. The continuous T-maze design was set for the animals to rely on different strategies based on local and distal cues. In this way, the animals expressed differential navigation preference. The rats with significant place preference navigation during the probe navigated in this manner not because of the reward location (equivalent reward was positioned in both corners) but because of the integration of the spatial cues and reward during the training sessions. T-maze and plus-maze are behavioral setups for global remapping and this is described as journey-dependent mapping [29]. For this reason, we have chosen T-maze maze protocol and, subsequently, we have confirmed the global remapping with our data. The cross correlation of hippocampal pairs indicated global remapping during the probe; however, the distribution of the place cells for some animals was spatially biased, suggesting that the remapping was not random. Our goal here was to show that accumulation of place fields relates to the degree of preferential navigation, even after scattered remapping of the fields.

### Biased configuration of place fields after global remapping

We showed that place cells from animals preferably navigating in the east loop of the maze expressed a higher degree of accumulation, represented by the SFC values. A key finding of our study shows that place field accumulation encodes location-specific reward valence independently from the spatial representation code, which is reset after the global remapping. This finding supports the hypothesis that the field accumulation reflects the reward location, corresponding to the degree of place preference. Spatial configuration of the place fields in different subregions of a large environment is related to geometric or contextual similarities of these subregions [30]. The place fields recorded from the preference group of rats were characterized with more asymmetric configuration, suggesting memory-mediated contextual difference between both loops of the maze. Our findings demonstrate that the spatial configuration of the place fields varies as a function of the experience but not the actual reward location (equivalent reward was presented in both maze corners during the probe). This finding suggests that the place cells from the reward loop of the training sessions integrated spatial and reward information on a population level within a functional engram [4, 6]. Thus, after the global remapping during the probe session, the reward loop cells were distributed predominantly in the section of the maze associated with higher experience-dependent reward value. To compensate for this spatial representation bias, the nonreward loop cells were distributed predominantly in the opposite loop of the maze during the probe. Our data support the proposal that place fields encode not only the spatial geometry but also the reward expectance across the environment [31].

### Spatial distribution of place cells is biased for preferred navigation

The study of population dynamics in hippocampal neurons is one of the most powerful tools for understanding the link between place fields and navigation [32, 33]. To examine the proposal that fields distribution relates to preferred location, we used a metric that evaluates the distribution of the place cells within the Cartesian plane, i.e., SPV. Weighted dimensional representation of the population dynamics is a new analytical technique in place field data analysis; however, it is already applied in the decoding of movement direction from motor cortex neuronal ensembles [34]. Using the common computational approach, here we propose that neuronal populations in different brain circuits share fundamentally similar mechanisms of information encoding and retrieval. The benefit of the SPV is that it can relate navigation to the change of assembly field distribution for different forms of remapping such as rate and place remapping for different experimental conditions and behavioral setups. Previous studies have investigated place cell spiking for individual journeys of plus-maze tasks [35–37]. Our experimental design included continuous navigation, which allowed for the estimation of place preference (measured after the evaluation of repeated behavior) in parallel with the formation of stable place fields (measured after the evaluation of repeated path sampling). The effect of egocentric inputs was reduced to minimum because the animals were allowed to navigate in opposing directions and the behavior at the choice points relied exclusively on allocentric signals. Thus, the directionality of the place fields did not affect the SPV values. To relate the degree of SPV to scale of preferred navigation, we induced variability of the place preference behavior among the animals. The dissociation of the distal and proximal cues [38] during the probe resulted in differential path navigation strategies with sufficient path sampling of both loops. Here, using assembly measures of the remapped place fields, we established a link between the spatial distribution of place fields’ ensemble activity and the animal’s preferred navigation. The firing rate of individual place cells contributes to the SPV, indicating why weighted SPV correlates better with the navigation preference when compared to the averaged SPV. The SPV did not depend on the path sampling because the firing rate accounted for the occupancy of each pixel. The data provide evidence that the hippocampal place field assembly code closely reflects experience-dependent navigation preference. Our findings show that hippocampal neurons are not merely mapping the environment but their spatial distribution encodes learning-adopted location of a prospective reward.

### Dopaminergic inputs regulate the distribution hippocampal place fields

To understand the underlying mechanisms of reward-dependent changes in place cells’ activity, we optogenetically manipulated their dopaminergic inputs. Optogenetic stimulation of hippocampal dopaminergic fibers arising from VTA in mice during spatial learning of novel locations improves the place field stability and stabilizes spatial memory performance [21]. An alternative way to show the importance of dopaminergic signaling to hippocampal spatial representation is suppression of the VTA-generated signal. Accordingly, we found that photoinhibition of dopaminergic VTA activity evoked reconfiguration of the place fields. We used a novel adeno-associated virus iC++, which triggered chloride-conducting photoinhibition after blue light application [20]. The suppression of VTA dopaminergic neurons evoked partial place field remapping and shifted the SPV towards the arm without photoinhibition. Our data provide direct experimental evidence of the relationship between the hippocampal assembly configuration and the activity of midbrain dopaminergic neurons, encoding salient information [39, 40]. The hippocampal neurons encode newly learned goal locations through the reorganization of assembly firing patterns in the CA1 region [33, 41] and this process is NMDA-receptor dependent [14]. Our findings highlight the important role of dopaminergic signaling in field assembly reorganization. Moreover, dopamine-dependent shifts in the SPV indicated the direction of behavioral navigation preference. Our results extend previous findings that knockout mice for dopamine receptor D1 receptor do not remap in response to environmental manipulation [42].

### Tegmental dopaminergic signals guide preferential navigation

A major advantage of the use of rats for behavioral optogenetic experiments is the high degree of path sampling in achieved continuous navigation experiments. This degree of path sampling is essential for the precise evaluation of the place field parameters, including the COM. We implanted transgenic TH::Cre rats with optic fibers in the lateral section of the VTA, a region with a high degree of segregation between the TH and GAD expressing neurons [43]. In addition, the expression of adeno-associated virus in TH cells shows varying degrees of selectivity and penetrance; for our expression, the selectivity was 52% for AAV-iC++. This degree of expression was sufficient for the behavioral response of laser light application in our rectangular-shaped linear maze. The photoinhibition successfully induced place avoidance after 2 sessions. This finding supports the proposal that midbrain dopaminergic inputs are central to the integration of salience and hippocampus-dependent memory in rodents [39, 40, 44]. Functional magnetic resonance imaging (fMRI) studies in humans also confirm that VTA activity correlates with enhanced learning in the context of novelty [45]. Optogenetic manipulation of VTA cells allowed us to regulate the reward inputs to the entire dorsal hippocampal network, compared to local stimulation of dopaminergic fibers. Activation of the sparsely connected dopaminergic projections can induce a sufficient effect on the hippocampal spatial network to reflect navigation preference. The system effect of VTA activity on behavior may involve also the ventral striatal neurons that generate firing patterns correlating to task events such as prospective rewards, goal locations, and sensory stimuli predicting rewards [46, 47]. Another source of dopaminergic regulation of hippocampal spatial representation may originate from locus coeruleus. Dopamine coreleasing TH+ neurons in locus coeruleus mediate postencoding memory enhancement and optogenetic activation of these cells evokes novelty-associated memory enhancement [48]. These recent findings propose that the dopaminergic innervation in the hippocampus may need to be further examined, with particular focus on the locus coeruleus. Locus coeruleus could exert a more potent effect on hippocampal physiology and spatial representation compared to VTA in particular tasks involving novelty exploration and memory formation [48].

### Spatial VTA activation determines the direction of field plasticity

The photostimulation of dopaminergic projections in the hippocampus after AAV-ChR2-YFP virus injection in VTA resulted in increased spiking of the place cells to 123.8%, while slow-spiking interneurons (with monosynaptic inputs from the place cells) gradually suppressed their firing to 78.4% throughout the light delivery protocol. The recorded slow-spiking interneurons in the CA1 pyramidal cell layer share characteristics similar to those of Ivy cells, which are interneurons expressing neuropeptide Y (NPY) or the neuronal nitric oxide (NO) synthase isoform [49]. Parvalbumin-expressing interneurons in the hippocampal CA1 pyramidal cell layer (basket, bistratified, and axo-axonic cells) often display a faster spiking pattern [50]. The firing rate of the Ivy cells recorded in behaving animals is 2.4 ± 1.8 Hz during theta episodes and 3.0 ± 3.6 Hz during non-theta episodes [49]. In our recordings, the average spiking of this group of interneurons was 3.29 ± 2.3 Hz. Paired recordings in vitro showed that Ivy cells receive depressing excitatory postsynaptic potentials from the pyramidal cells [49]. Similarly, we found that photostimulation-augmented place cell activity was paralleled by gradually suppressed firing of postsynaptic slow-spiking interneurons. Reciprocally, Ivy cells regulate the excitability of pyramidal cell dendrites through slowly rising and decaying GABAergic inputs [49]. Increased firing rate of place cells through disinhibition has been recently proposed as one of the hippocampal mechanisms for rate remapping [51]. A similar mechanism might mediate the rate remapping observed in our recordings, while the field remapping may reflect a dopamine-specific plasticity mechanism. The observed suppression of hippocampal slow-spiking interneurons might facilitate the experience-dependent plasticity effect of dopaminergic inputs on the place cells. Concurrently, we show that optogenetic activation of the dopaminergic VTA neurons evoked a gradual shift of the COM of the place fields when the dopaminergic neurons were activated in close proximity to the place field. Furthermore, the bias of the observed ΔCOM indicated the spatial direction of dopaminergic photostimulation. Thus, the remapping of COM was evoked by location-specific dopaminergic activation. The direction of place field plasticity was evaluated by the Bhattacharyya distance overlap. The observed reduction of bhatt values indicated ΔCOM was due to displacement in the direction of the stimulation coordinates. Our data show dopamine-dependent place field plasticity, which may be the spatial substrate of dopamine-mediated long-term synaptic plasticity. Blockade of dopamine D1/D5 receptors in CA1 impairs the long-term synaptic plasticity in vivo [52]. Furthermore, novelty-induced release of dopamine in the hippocampus [53] facilitates long-term plasticity, which is prevented by blocking of D1/D5 receptors [54]. Finally, long-term synaptic plasticity is believed to be the cellular mechanism of learning and memory [55].

In summary, our data show that place cells do not passively map the spatial environment in a Cartesian coordinate system but continually tune their fields to encode the location of prospective reward on population level. The place fields’ spatial plasticity is the key element in their ability to undergo assembly redistribution in order to mediate memory formation.

## Materials and methods

### Ethics statement

We conducted our experiments in accordance with directive 2010/63/EU of the European Parliament, the council of 22 September 2010 on the protection of animals used for scientific purposes, and the S.I. No. 543 of 2012 and followed the Bioresources Ethics Committee, Trinity College Dublin, Ireland (individual authorization number AE19136/I037; procedure numbers 230113–1001, 230113–1002, 230113–1003, 230113–1004, and 230113–1005), and international guidelines of good practice under the supervision of Marian Tsanov, who is licensed by the Irish Medical Board (project authorization number: AE19136/P003). Lister hooded rats have been chosen for these experiments because of their anatomical and physiological similarities to humans. Several steps were taken to minimize stress in the animals, which helped during surgery and recovery. Animals were handled and allowed to grow accustomed to their environment well before surgery. The surgery was completed as quickly and safely as possible to reduce the recovery time. The surgery itself was undertaken in aseptic conditions to reduce the risk of infection and anesthesia was carefully monitored to ensure that the animal was not stressed or in pain. After the surgery, a painkiller and antibacterial were administered to aid in recovery. We undertook to comply with all the ethical and security issues by appropriate protocols to ensure the application of the 3 R’s (reduction, replacement, and refinement).

### Animals

Male, 3–6-month-old, Lister hooded TH::Cre rats (Rat Resource & Research Center P40OD011062, United States) were individually housed for at least 7 days before all experiments under a 12-h light–dark cycle. The animals accessed water ad libitum. Rats were food deprived to 80% of their original weight. The recording sessions were conducted during the light phase.

### Surgical implantation of recording electrodes

Eight tetrodes were implanted in the hippocampal CA1 area: −3.8 AP, 2.3 ML, and 1.8 mm dorsoventral to dura. For recordings of dopaminergic neurons, 8 tetrodes were implanted unilaterally in VTA: 5.7 AP, 1.9 ML, angle 10^o^ medially, and 8.0 mm dorsoventral to dura. We targeted the lateral VTA due to greater colocalization of TH and targeted dopaminergic neurons in the TH-Cre rodent lines compared to the medial VTA [56].

### Recording techniques

The recordings were performed as previously described [57]. After a minimum 1-week recovery, subjects were connected via a 32-channel headstage (Axona Ltd.) to a recording system, which allowed simultaneous animal position tracking. Signals were amplified (10,000–30,000-fold) and band-pass filtered between 380 Hz and 6 kHz for single-unit detection. To maximize cell separation, only waveforms of sufficient amplitude (at least 3 times the noise threshold) were recorded. Candidate waveforms were discriminated offline using graphical software (Tint, Axona Limited), which allows waveform separation based on multiple features including spike amplitude, spike duration, maximum and minimum spike voltage, and the time of occurrence of maximum and minimum spike voltages. To verify that spike sorting analysis targets the recording from individual cells, we examined the following parameters for each unit: (1) waveform, the spike from a single neuron is characterized with consistent amplitude and time of occurrence of maximum and minimum spike voltages, (2) spike cluster, the spike cluster of an individual spike has a characteristic shape and it is located at consistent coordinates on the cross-amplitude scatterplots, (3) spike autocorrelogram, the first 2 ms represent the refractory period. Autocorrelation histograms were calculated for each unit, and the unit was removed from further analysis if the histogram presented spiking within the first 2 ms (refractory period), inconsistent with good unit isolation. Only stable recordings across consecutive days were further analyzed. The stability of the signal was evaluated by the cross correlation of spike amplitudes and similarity comparison of the spike clusters between the training sessions. Electrode stability was assessed offline by comparison of waveforms and cluster distributions. The single-unit signals of the last recording session and the probe were compared for waveform similarity, cluster location, size, and boundaries. Peak and trough amplitudes of the averaged spike waveforms were compared by Pearson’s *r* [36]. *r* values ≥ 0.9 indicated that the same populations of cells were recorded throughout the last recording session and the probe.

### Hippocampal unit identification and spatial firing analysis

We analyzed the single-unit recordings as previously described [57]. Single hippocampal pyramidal cells and interneurons were identified using spike shape and firing frequency characteristics [58]. Firing rate maps allow for visual inspection of neurons’ preferred areas of firing (i.e., place fields). They were constructed by normalizing the number of spikes that occurred in specific pixelated coordinates by the total trial time the animal spent in that pixel. This produced maps depicting the place fields of each cell and quantified in Hz (smoothed maps). Place field was defined as the region of the arena consisting of at least 9 adjacent bins, which contain spikes. We defined place field size as the region of the arena in which the firing rate of the place cell was 20% or greater of the maximum firing frequency [59]. We used multiple measures to analyze the spatial properties of the hippocampal place cell firing (i.e., place field size, spatial selectivity, spatial coherence, and spatial specificity information content). The spatial information of a firing field (ratio of maximal signal to noise) was calculated by dividing the firing rate of the cell in the bin with the maximum average rate by its mean firing over the entire apparatus. Spatial coherence consists of a spatial autocorrelation of the place field map and measures the extent to which the firing rate in a particular bin is predicted by the average rate of the 8 surrounding bins. Thus, high positive values result if the rate for each bin can be better predicted from the firing frequency of a neighboring location. With each spatial autocorrelation performed on the place field map, a *p* value is calculated, indicating whether the correlation is statistically significant. Place field activity is not considered to be spatially coherent if the *p* value is greater than 0.001. The spatial information (or spatial specificity) is expressed in bits per spike and is calculated as follows

$$I=\sum_{i}P_{i}(\lambda_{i}/\lambda )\;\log_{2}\left(\lambda_{i}/\lambda \right)$$

where λ_i_ is the mean firing rate in bin *i*, λ is the overall mean firing rate, and *P*_*i*_ is the occupancy probability of bin *i*. The spatial specificity index is a measure of the amount of information about the location of the animal conveyed by a single spike generated by a single place cell.

### Global remapping

Global remapping indicates relocation of the place field for each of the recorded place cells. Due to the binning of the T-maze (10 cm width, 85 cm length of the leg arms) for place field analysis (2.5 cm/bin), the chance of a place field composed of ≥3 x 3 bins to appear on the same location during a random global remapping is 1/43 (2.32%). Consistent with this prediction, 5/179 (2.79%) of the remapped cells kept their location in respect to the maze geometry (mirror representation), while 6/179 (3.35%) kept their location in respect to the distal cues (opposite representation). The global remapping was evaluated by the cross correlation of pairs of cells between the training session and the probe, which represents the degree of spatial overlap between the place cells. Color-coded cross-correlation matrices visualize the Spearman’s correlation between each pair of cells. Full overlap of the maps is denoted with red and a value of 1; no overlap is denoted with green and a value of 0, and a spatially inversed map is denoted with blue and a value of −1. For statistical analyses, the correlations were transformed into z values.

### Continuous T-maze task

The continuous T-maze task evoked a reward-driven place preference, the location of which was associated with both maze geometry (source of proximal cues) and distal allocentric cues. The identification of the distal cues was designed in a manner in which approximately half of the animals would rely on them for spatial navigation (see below). During the training sessions, the animals were placed on the starting choice point and allowed to explore the maze (10 cm width, 85 cm length of the leg arms). The access to the opposite T-maze was disconnected. Two pellets (TestDiet, Formula 5TUL) were continuously positioned at the end of the SW corner (reward zone), while no pellets were positioned in the NE corner. In 33% of the passes, 1 pellet was positioned at the central choice point and in 33% of the passes, 1 pellet was positioned at the starting choice point. In the remaining 33% of the passes, no pellet was present at the choice points. The sequence of pellet positioning in the choice points was random. In this way, the animals adopted continuous locomotion in a loop pattern through the choice points. Direct passes between the SW and NE zones were not rewarded with pellets. The animals were allowed to navigate in both clockwise and anticlockwise directions of both arms. As such, the animals were not able to rely only on egocentric learning, as the central and the starting choice points required opposite head turns. Thus, the animals were required to learn to navigate with reference to proximal cues (choice point shapes) and/or distal cues (white plus, grey star, blue square, and yellow circle signs attached on the black curtains surrounding the maze). The duration of each trial was 12 minutes. Rats were given 3 daily training trials over 3 consecutive days (9 trials in total). On Day 4, the animals were given the probe trial. During the probe trial, the animals were placed on the starting choice point of the opposite T-maze, while access to the training T-maze was disconnected. In this way, the rats were exposed to the same proximal but opposite distal cues. Two pellets were constantly positioned in both the SW and NE corners. The training and the probe recordings took place in a room with 4 distal cues (size A4) and luminosity of 10–15 lux. The luminosity was set at a level such that there was approximately 50% probability that the rats would rely on allocentric navigation, dependent on distal cues [28]. The luminosity level determines the navigation strategy of the animals in the probe session of the continuous T-maze task. High luminosity (>20 lux) allowed the rats to identify the distal visual cues and use them as a spatial reference. During the probe, the conflict between the distal and proximal (choice point shapes) cues results in a split navigation strategy towards both arms. Low luminosity (<5 lux) results in navigation guided predominantly on the proximal maze cues, as the distal cues are an insufficient reference for spatial orientation. The task was designed to distinguish between allocentrically guided preferential and nonpreferential navigation, with a sufficient number of passes in the nonpreferential section of the maze. An insufficient number of passes results in incomplete experience for the formation of place fields and invalidates the evaluation of their properties [12]. Hippocampal neurons require 5–6 minutes of experience to form a stable spatial representation in a novel environment [13].

### Binomial probability

The chance level of preferential versus nonpreferential navigation was based on the number of passes from the choice points towards each of the corners, given the total number of passes. The binominal probability mass function was calculated as follows

$$f\;(x;n,p)=\frac{n!}{x!(n-x)!}\;p^{x}\;{\left(1-p\right)}^{n-x}$$

where *x* is the number of passes from the choice points towards 1 direction (south or east loops), *n* is the number of passes, and *p* is the probability of a pass outcome towards a certain direction.

### SFC

SFC for continuous T-maze was based on a smoothed rate map with the size [M x M] bins and the corresponding place field map containing *N* place subfields. The resulting map is grouped to 6 levels (>0, >1/6 f_max,_, >2/6 f_max_, >3/6 f_max_, >4/6 f_max_, >5/6 f_max_) according to the maximum firing frequency of the rate map (f_max_) and separated into leveled place fields. The maps are binary. For each level *l* {*l* ∈ ℕ| 2 ≤ *l* ≤ 6} an [N x N] relation matrix is created by the overlap between each map (*m*_*n*,*l*_) and each transposed map ($m_{m,k}^{t}$) of the same or lower level and the transposed place field map (${pf}_{p}^{t}$)

$$R_{l}=\left[\begin{array}{cccc} r_{1,1,l} & r_{1,m,l} & \cdots & r_{1,N,l}\\ r_{n,1,l} & r_{n,m,l} & \cdots & r_{n,N,l}\\ \vdots & \vdots & \ddots & \vdots \\ r_{N,1,l} & r_{N,m,l} & \cdots & r_{N,N,l} \end{array}\right]$$

The [N x N]-sized relation matrix displays the relations between place field *n* at level *l* and place field *m*:

$${r_{n,m,l}=A}_{n,l}^{-1}∙o_{n,m,l}$$

The relation between place field *n* at level *l* and place field *m* (*r*_*n*,*m*,*l*_) is the overlap between place field *n* at level *l* and place field *m* (*o*_*n*,*m*,*l*_), normalized by the area of place field *n* at level *l*(*A*_*n*,*l*_).

$$o_{n,m,l}=\sum_{i=1}^{M}\sum_{j=1}^{M}m_{n,l}(i,j)∙({pf}_{m}^{t}(i,j)+\sum_{k=2}^{l}m_{m,k}^{t}(i,j))$$

The overlap between place field *n* at level *l* and place field *m* is the sum of the product of the value of bin (*i*,*j*) of place field *n* at level *l* (*m*_*n*,*l*_(*i*,*j*)) and the value of the bin (*i*,*j*) transposed place field map (${pf}_{m}^{t}(i,j)$) and the value of the bins (*i*,*j*) transposed maps of place field *m* at all levels equal and smaller level ($m_{m,k}^{t}(i,j)$).

$$A_{n,l}=l∙\sum_{i=1}^{M}\sum_{j=1}^{M}m_{n,l}(i,j)$$

where A_n,l_ is the area, expressed by the number of bins, of place field *n* at level *l* weighted by the level *l*.

From the relation matrix, we calculate the SFC (*SFC*_*l*_) for each level by summing the rows of the relation matrices and weighting these sums by the ratio of the area of place field *n* at level *l* to the sum of the areas of all place fields in level *l* (*w*_nl_).

$${SFC}_{l}=\sum_{n=1}^{N}w_{n,l}\sum_{m=1}^{N}r_{n,m,l}$$

$$w_{n,l}=\frac{A_{n,l}}{\sum_{n=1}^{N}A_{n,l}}$$

The place SFC of the firing characteristics of the cell is the average of the field distributions of each level weighted by the ratio of the area of all place fields in level *l* to the sum of the area of all place fields in all levels (*A*_*total*_)

$${SFC}_{spat=}\sum_{l=2}^{6}{SFC}_{l}∙\frac{\sum_{n=1}^{N}A_{n,l}}{A_{total}}$$

$$A_{total}=\sum_{l=2}^{6}\sum_{n=1}^{N}A_{n,l}$$

where *SFC*_spat_ is the SFC value.

### COM

The COM of the place cells’ spike distribution is calculated as follows

$${COM}_{x}=\frac{\sum_{i=1}^{N_{x}}\sum_{j=1}^{N_{y}}f_{ij}∙i}{\sum_{i=1}^{N_{x}}\sum_{j=1}^{N_{y}}f_{ij}}∙l_{bin}-\frac{l_{bin}}{2}$$

$${COM}_{y}=\frac{\sum_{i=1}^{N_{x}}\sum_{j=1}^{N_{y}}f_{ij}∙j}{\sum_{i=1}^{N_{x}}\sum_{j=1}^{N_{y}}f_{ij}}∙l_{bin}-\frac{l_{bin}}{2}$$

where *N*_x_, *N*_y_ define the number of bins in the arena in X-, Y- direction; *f*_i,j_ is firing frequency in bin *i*, *j*; and *l*_bin_ is the bin size.

Given the origin O (O_x_,O_y_), which denotes the NW corner of the Cartesian coordinate system, and the direction of the symmetry axis D (D_x_,D_y_), which denotes the line between the SW and NE corners, the distance of the COM (COM_x_,COM_y_) to the symmetry axis is calculated as follows

$${dist}_{COM/sym}=\frac{\left|det[\begin{array}{cc} D_{x}-O_{x} & D_{y}-O_{y}\\ {COM}_{x}-O_{x} & {COM}_{y}-O_{y} \end{array}]\right|}{\sqrt{{\left(P_{x}-O_{x}\right)}^{2}+{\left(D_{y}-O_{y}\right)}^{2}}}$$

where *P* is the shortest distance between the COM and the symmetry line. For the rectangular-shaped linear track, the arena borders are defined as the square surrounding all motion-tracking sample points with an equal distance to the real limits of the arena at all sides. For the continuous T-maze track, the borders are defined as the hypotenuse of the arena, which is congruent with the diagonal connecting the SW and the NE corners.

Using *dist*_COM_, we calculated the distance between O and P as follows:

$$\bar{OP}=\sqrt{({{COM}_{x}}^{2}+{{COM}_{y}}^{2})-{{dist}_{COM}}^{2}}$$

The COM distance normalized by the arena width perpendicular to the symmetry axis through the COM is

$${dist}_{norm}=\left\{\begin{array}{c} \frac{{dist}_{COM}}{\bar{OP}∙C},\bar{OP}<\frac{\bar{OM}}{2}\\ \frac{{dist}_{COM}}{(\bar{OM}-\bar{OP})∙C},\bar{OP}>\frac{\bar{OM}}{2} \end{array}\right\}$$

where $\bar{OM}$ is the diagonal of a square enclosing all motion-tracking data points and *C* is a motion-tracking data factor set to 0.95 for the continuous T-maze and 0.85 for the linear rectangular track.

### COM angle

The COM angle is then calculated as follows

$$\theta_{COM}=\left\{\begin{array}{c} 45{}^{\circ}∙(1-{dist}_{norm}),{COM}_{x}<{COM}_{y}\\ 45{}^{\circ}∙(1+{dist}_{norm}),{COM}_{x}>{COM}_{y} \end{array}\right\}$$

Using an SFC angle *θ*_*SFC*_, we set a numerical value for the SFC, *SFC*_spat_ (0: no symmetry, 1: maximum symmetry), and the location of the COM:

$$\theta_{SFC}=\left\{\begin{array}{c} {SFC}_{spat}*45{}^{\circ},{COM}_{x}<{COM}_{y}\\ 90{}^{\circ}-{SFC}_{spat}*45{}^{\circ},{COM}_{x}>{COM}_{y} \end{array}\right\}$$

*SFC*_spat_: SFC value; COM_x_, COM_y_: X-, Y- coordinate of COM

### SPV

We used the population vector of the place field distribution *D* as well as the grand rate population vector *F* to describe the common behavior of the whole population of place cells recorded. The population vector of place field distribution consists of the COM angle of each cell *θ*_COM,n_, where *n* indicates the cell index

$$D_{n}=\theta_{COM,n}$$

$$F_{n}=\frac{s_{n}}{{\Delta t}_{tot}}$$

where *S*_n_ is number of spikes of cell *n*; Δ*t*_*t*ot_ is duration of the measurement.

The SPV weighted by the mean firing frequency of the cells *SPV*_weighted_ is calculated as the dot product of the grand rate population vector *F* and the population vector of place field distribution *D* normalized by the 1-norm of the grand rate population vector

$${SPV}_{weighted}=\frac{F∙D}{{\left\|F\right\|}_{1}}$$

and the average SPV, SPV_avg_, is defined as the 1-norm of the population vector of place field distribution *D*, normalized by the number of place cells *N* in the population

$${SPV}_{avg}=\frac{{\left\|D\right\|}_{1}}{N}$$

The 1-norm of a vector is defined as the sum of its elements

$${\left\|F\right\|}_{1}=\sum_{n=1}^{N}\left|F_{n}\right|;\;{\left\|D\right\|}_{1}=\sum_{n=1}^{N}\left|D_{n}\right|$$

All algorithms were created using MATLAB.

### Virus construction and optical activation

We used a Cre-inducible viral construct designed for optogenetic purposes [22, 60]. pAAV5-Ef1a-DIO-hChR2(E123T/T159C)-EYFP-WPRE-pA was serotyped with AAV5 coat proteins and packaged by Vector Core at the University of North Carolina. Viral titers ranged from 1.5–8 x 10^12^ particles per mL [22]. AAV8-EF1a-DIO-iC++-TS-EYFP was serotyped with AAV8 coat proteins, in a titer of 4.3 x 10^12^ particles per mL, provided by Karl Deisseroth (Stanford University). For control experiments, we used virus bearing only the YFP reporter [22]. Randomization of group allocation (iC++ or E123T/T159C versus YFP controls) was performed using an online randomization algorithm (http://www.randomization.com/). The virus injection was applied unilaterally in the VTA (5.7 AP, 1.9 ML, angle 10° medially), with volume of 2 μl injected on 2 levels: 1 μl at 8.0 mm and 1 μl at 9.0 mm dorsoventral to the dura. Subsequently, an optical fiber (200-μm core diameter, Thorlabs, Incorporated) was chronically inserted (5.7 AP, 1.9 ML, 8.0 DV, angle 10° medially). Simultaneous optical stimulation of the VTA and extracellular recording from CA1 were performed in freely behaving rats 3 weeks after the surgery. For the concurrent recordings in the hippocampal CA1 region, the optical fiber was inserted inside the microdrive cannula (Axona, Limited) of the recording tetrodes, with the tip of the tetrodes projecting beyond the fiber by 500 μm, and the optical fiber was coupled to a 473-nm laser (Thorlabs, Incorporated). The light power was controlled to be 10–15 mW at the fiber tip. Square-wave pulses with duration of 5 ms were delivered at frequency of 50 Hz.

### Rectangular-shaped linear track

The animals were trained to navigate between the SW and NE corners, where 2 pellets were continuously positioned. The animals were allowed to navigate in both clockwise and anticlockwise directions of the rectangular-shaped linear track (10 cm width, 85 cm length of the arms). For the optic stimulation sessions, the laser was switched on when the animal entered the north arm or the west arm, with continuous photostimulation trains (473 nm, 50 Hz, 5 ms pulse duration, 12 pulses per train, 0.5 sec intertrain interval) until the animal exited this section of the track. The duration of each session was 12 minutes. Because the detection of our dependent variable (navigation preference) was independent of the experimenter, we did not use a blinding process for group allocation or behavior scoring.

### Open field arena

For the open field recordings, the rats were placed in a square arena (60 x 60 cm) and 20-mg food pellets were thrown in every 20 seconds to random locations within the open field (pellet-chasing task); in this way, animals locomoted continuously, allowing for complete sampling of the environment. For optogenetic stimulation of dopaminergic fibers in the hippocampus, we applied photostimulation (473 nm, 50 Hz, 12 pulses, 5 ms pulse duration) every 6 seconds during a recording session with duration of 12 minutes. We evaluated the pre- and poststimulation firing rates for 100 and 250 ms before and after the onset of the blue light train. For optogenetic stimulation of dopaminergic VTA neurons, a single train of photostimulation (473 nm, 50 Hz, 12 pulses, 5 ms pulse duration) was applied when the animal crossed the borders of the selected quadrant. The experiment consisted of 4 recording sessions: first a baseline, followed by a first photostimulation session; 24 hours later, a second baseline (baseline 2) was followed by a second photostimulation session (ChR2 2). The blue laser was synchronized with the video tracking and with the recoding system through DACQBASIC scripts (Axona. Limited). The chosen duration of 12 minutes allowed the rats to explore evenly the arena in 2 subsequent recordings per day (baseline and stimulation sessions) for 2 consecutive days. Baseline recordings >15 minutes result in insufficient exploration of the subsequent stimulation session, while recordings <10 minutes reduced the sampling of the explored environment. The rats were habituated to the square arena before the recordings.

### Bhattacharyya distance

Bhatt is a parameter that quantifies the distance between 2 equally sized probability distributions [61], in which a complete overlap of 2 identical distributions is 0 bhatt. Bhattacharyya distance values are unaffected by the scale of the distributions. The Bhattacharyya distance J_B_ between the distributions p_1_ and p_2_ is given by:

$$J_{B}(p_{1},p_{2})=-log\int_{x}^{}\sqrt{p_{1}(x)∙p_{2}(x)}dx$$

### MFB stimulation

We delivered electric current through electrodes (SNEX-300, Kopf Instruments) implanted in the MFB: −3.3 AP, 1.8 ML, and 7.8 mm dorsoventral to dura. The stimulation protocol was generated by a constant current bipolar stimulus isolator (A365D, World Precision Instruments, Incorporated), which was controlled through a TTL input from the recording system [57]. The protocol consisted of 4 bursts, with each burst containing 3 pulses at 10 ms (100 Hz), with an intertrain interval of 125 ms (8 Hz). The current intensities were in the range of 50–200 μA [62] and were fine-tuned individually with respect to the amplitude of the test-pulse stimulus artifact. The stimulus isolator was synchronized with the video-tracking system through DACQBASIC scripts (Axona. Limited). The electrical stimulation was applied when the animal crossed the borders of the selected quadrant. The experiment consisted of 4 recording sessions: first a baseline, followed by a first MFB stimulation session; 24 hours later, a second baseline (baseline 2) was followed by a second MFB stimulation session (MFB 2).

### Histology

At the end of the study, brains were removed for histological verification of electrode localization, as previously described [57]. Rats were deeply anesthetized with sodium pentobarbital (390 mg⁄kg) and perfused transcardially with ice-cold 0.9% saline followed by 4% paraformaldehyde. Brains were removed, postfixed in paraformaldehyde for up to 24 hours, and cryoprotected in 25% sucrose for >48 hours. Brains were sectioned coronally at 40 μm on a freezing microtome. Primary antibody incubations were performed overnight at 4°C in PBS with BSA and Triton X-100 (each 0.2%). The concentration for primary antibodies was anti-TH 1:200 (Millipore, #MAB318). Sections were then washed and incubated in PBS for 10 minutes and secondary antibodies were added (1:200) conjugated to Alexa Fluor 594 dye (Invitrogen, # A11032) for 2 hours at room temperature. For visualization, the sections were mounted onto microscope slides in phosphate-buffered water and coverslipped with Vectashield mounting medium. The YFP fluorescence was evaluated within a selected region that was placed below the fiber tip in an area of 1.5 x 1.5 mm. Fluorescence was quantified based on the average pixel intensity within the selected region [22]. The stained sections were examined with an Olympus BX51 fluorescence microscope and an Olympus IX81 confocal microscope at 594 nm for Alexa Fluor secondary antibody and 488 nm for ChR2-YFP. TH-positive neurons were identified based on expression of red fluorescence, whereas ChR2-positive neurons were identified by expression of green fluorescence. Colocalization of Alexa Fluor 594 and YFP was determined manually using ImageJ software (Image Processing and Analysis in Java).

### Quantification and statistical analysis

Two different approaches were used to calculate the sample size [63]. We performed power analyses to establish the required number of rats for experiments in which we had sufficient data on response variables. For experiments in which the outcome of the intervention could not be predetermined, we used a sequential stopping rule. This approach allows null-hypothesis tests to be used subsequently by analyzing the data at different experimental stages using *t* tests against type II error. The experiment was initiated with 4 animals per group; if the *p* reached a value below 0.05, the testing was continued with 2 or 3 more animals to increase the statistical power. In case of *p* > 0.36, the experiment was discontinued and the null hypothesis was accepted [63]. All data were analyzed using SPSS Software. Statistical significance was estimated by using a 2-tailed independent samples *t* test for nonpaired data or a paired samples Student *t* test for paired data. Repeated measures were evaluated with 2-way analysis of variance (ANOVA) paired with post hoc Bonferroni test. Correlations between datasets were determined using Pearson’s correlation coefficient. The probability level interpreted as significant was fixed at *p* < 0.05. All data points are plotted ± SEM.

## Supporting information

S1 Fig Differential navigation in continuous T-maze task. (A) Navigation trajectory from the preference group of animals (n = 10) during the last training session (left panels) and during the probe (middle panels). The right panels show the respective time rate maps, where darker grey represents pixels with longer dwell time. The numbers on the right show the SW/NE passes ratio for each animal. They are presented in order from the lowest to the highest value. (B) Navigation trajectory from the non-preference group of animals (n = 10) during the last training session (left panels) and during the probe (middle panels). The right panels show the respective time rate maps, where darker grey represents pixels with longer dwell time. The numbers on the right show the SW/NE passes ratio for each animal. They are presented in order from the lowest to the highest value. Files dataset is available at Figshare public repository in Tsanov 2016 data / Continuous T-maze folder https://figshare.com/s/b86a9a111353ba04bd32 and Tsanov 2017 data / Continuous T-maze CA1 folder https://figshare.com/s/5c5ba9b2811f3d7b7696.
(TIF)

S2 Fig Stability of recorded single-units during global remapping. (A) Scatterplot, showing the signals from multiple units recorded between each pair of electrodes on a given tetrode from sample training session. The color-coded clusters represent the spikes from each unit at the scatterplot. The spike sorting technique compares the amplitude of the recorded signal between each electrode tip (A1, A2, A3, A4). The maximal waveform amplitude of each unit is measured at different electrode. Each waveform is represented by different spike shape, measured by the peak-trough amplitude of the spike. (B) Spike clusters of multiple cells from probe session. To confirm the stability of the signal after multiple recordings across consecutive days the spike waveform and the position of the spike clusters in the 6 electrode-pair scatterplots were examined between recording sessions. The stability of the waveform is evaluated by the position of each spike cluster on the two-dimensional comparison by the peak-trough amplitude on one electrode against the peak-trough amplitude on another. The probe session was characterized with activation of a new place cell (the new spike cluster is marked with black asterisk). (C) Displacement of the tetrodes results in simultaneous change of the clusters location across all electrode tip pairs. The scaterplot shows the rearrangement of the spike clusters after 50 μm lowering of the implanted microdrive. Note the change of the clusters locations compared to the training (A) and probe sessions (B) per each electrode pair. (D) The stability of the spike signal between the training (left) and probe session (middle) is evaluated by the peak-trough amplitude of the spike and the the time of occurrence of maximum and minimum spike voltages for all four electrode channels. The highest amplitude of the recorded signal for the blue spikes is expressed at the third electrode channel. Note the change of the spike shape at the third electrode after 50 μm microdrive lowering (right). Note also the increase of the spike amplitude in the rest of the electrode channels. (E) Above: colour-coded cross-correlation matrix of the raw maps for simultaneously-recorded cells during training (y-axis) and control probe sessions (x-axis) from a control rat. Below: Colour-coded cross-correlation matrix of the smoothed maps for the same cells. For the control probe the maze was not rotated but kept in the same position as during the preceding training session. Each bin represents Spearman’s correlation between a pair of cells, which shows the degree of spatial overlap between them. Full overlap of the maps is denoted with red and value of 1, no overlap–green and 0, and spatially inversed map with blue and– 1. The abbreviations #t#c represent the number of recording tetrode and the number of recording cell, respectively. (F) Colour-coded cross-correlation matrices of the smoothed maps recorded from four sample rats. Note the less symmetric distribution of the coloured bins across the symmetry axis (the diagonal marked by the white bins) compared to the control probe (E). (G) Cross-correlogram of the smoothed rate maps from a control rat. Spearman’s r = 0.72, *P* < 0.001. (H) Cross-correlogram of the smoothed rate maps from four sample rats. Spearman’s r = 0.04, *P* = 0.73, Spearman’s r = -0.05, *P* = 0.55, Spearman’s r = 0.11, *P* = 0.24, Spearman’s r = 0.01, *P* = 0.89. For statistical analyses the correlations were transformed into Z-values. Files dataset is available at Figshare public repository in Tsanov 2017 data /T-maze field correlations individual pairs / T-maze field correlations all pairs per rat folder https://figshare.com/s/5c5ba9b2811f3d7b7696.
(TIF)

S3 Fig Remapping of the place cells during T-maze probe session. (A) Color-coded firing rate maps of 45 sample place cells recorded during the training (left) and probe session (right) from 5 rats from the preference group (rats 1–5). (B) Color-coded firing rate maps of 45 sample place cells recorded during the training (left) and probe session (right) from 5 rats from the non-preference group (rats 6–10). The number of recorded spikes of each place cell is shown below the maps. Files dataset is available at Figshare public repository in Tsanov 2016 data / Continuous T-maze folder https://figshare.com/s/b86a9a111353ba04bd32 and Tsanov 2017 data / Continuous T-maze CA1 folder https://figshare.com/s/5c5ba9b2811f3d7b7696.
(TIF)

S4 Fig Evaluation of the center of mass spatial location during the probe. (A) Three sample place cells recorded from the reward loop of the training sessions (reward loop cells) from three representative animals. Upper panels show the animal trajectory with spikes, marked with colored dots and their color-coded firing rate map (right) from the last training session. Lower panels show the animal trajectory with spikes (left) and their place fields’ color-coded firing rate maps (right) from the probe session. The center of mass (COM) is indicated with black mark at the end of the red line. The straight red line denotes the direction (in degrees) of center of mass angle (COMa) between SW at 0°, NE at 90° with midline at 45°. (B) Sample place cell which was absent from the reward loop of the training sessions (non-reward loop cells). (C) COM angle values from the preference and non-preference groups are reported for the reward loop cells. Two-tailed independent *t*-test test, left: for the non-reward loop cells n = 90 cells (preference group), n = 89 cells (non-preference group), *t*(177) = 5.477, ***P < 0.001; middle: for the non-reward loop cells, n = 33 and n = 30, respectively, *t*(61) = -2.137, *P = 0.037; and left: for all cells, n = 123 and n = 119, respectively, *t*(240) = 3.274, **P = 0.001. Error bars, mean ± s.e.m. (D) Schematic representation of the COM location (indicated with black mark at the end of the red lines) for the four cells from (A-B). The red line connects COM with the starting coordinate to form an angle with the horizontal dashed line indicating 0 degrees. (E) Spike waveform of a sample place cells from each animal (n = 20), recorded from the last training session (above) and from the probe (below). For each waveform, the solid line is the average waveform shape, and the dashed lines show the 1 SD confidence intervals. The *y*-axis scale denotes the amplitude of the action potential in microvolts (negativity is up), and the dotted horizontal line through 0 denotes the baseline potential. The length of the *x*-axis represents 1 ms. Files dataset is available at Figshare public repository in Tsanov 2016 data / Continuous T-maze folder https://figshare.com/s/b86a9a111353ba04bd32 and Tsanov 2017 data / Continuous T-maze CA1 folder https://figshare.com/s/5c5ba9b2811f3d7b7696.
(TIF)

S5 Fig The place field assembly distribution differs between the animals from the preference and non-preference groups. (A) Spatial distribution of the spikes (colored dots) from the reward loop cells (represented by different colors) recorded from seven preference group animals and (B) from seven non-preference group animals. The straight red line denotes the weighted spatial population vector (SPV in degrees) between SW at 0° and NE at 90°. (C) Spatial distribution of the spikes (colored dots) from the all cells (represented by different colors), including reward and non-reward loop cells recorded from the preference and (D) non-preference group animals, respectively. Files dataset is available at Figshare public repository in Tsanov 2016 data / Continuous T-maze folder https://figshare.com/s/b86a9a111353ba04bd32 and Tsanov 2017 data / Continuous T-maze CA1 folder https://figshare.com/s/5c5ba9b2811f3d7b7696.
(TIF)

S6 Fig Forced navigation during the probe of continuous T-maze task. (A) Biased navigation trajectory from the sample animal during the last training session (left panel) and during the probe (right panel). After the animal explored the east track of the maze in each direction, the access to this arm was restricted. If the rat was approaching towards the east track either from the north or from the south side (marked with red arrows) the experimenter actively guided the animal towards the opposite direction. As a result, the rat navigated four times more often towards SW compared towards NE corner (the SW/NE passes ratio = 4) from the choice points. The forced navigation technique was chosen instead of compartmental obstruction for particular section of the maze. The compartmentalization of recording arena evokes remapping of place fields [1]. (B) Respective time rate map, where darker grey represents pixels with longer dwell time. Note that SW corner is the location with the longest dwell time. (C) Weighted spatial distribution of the spikes (colored dots) from the all cells (represented by different colors) recorded, with SPV value of 52.6°. The weighted SPV value for the spikes only from the reward loop cells was 51.3°. Files dataset is available at Figshare public repository in Tsanov 2017 data / Continuous T-maze forced navigation folder https://figshare.com/s/5c5ba9b2811f3d7b7696. 1. O'Keefe J, Burgess N. Geometric determinants of the place fields of hippocampal neurons. Nature. 1996;381(6581):425–8. 8632799.
(TIF)

S7 Fig Tegmental slow-spiking activity bias for the preferred navigation during the probe of continuous T-maze task. (A) The behavioral set-up of the continuous T-maze for the ventral tegmental recordings was the same as for the hippocampal recordings. In this case we evaluated the direction of the passes from the starting choice point (marked with a white arrow) and the central choice point as well as the firing rate of the recorded neurons for each direction (towards SW versus towards NE direction). (B) Representation of the passes from preference group rat towards the SW corner (in red) and towards NE corner (in blue) from the central choice point (above) and from the starting choice point (below). (C) Comparison of the firing rate ratio of southwest (SW) to northeast (NE) passes. Error bars, mean ± s.e.m., n = 16 cells from 3 rats (preference group), n = 14 cells from 4 rats (non-preference group), two-tailed independent *t*-test test, *t*(28) = -6.737, ***P < 0.001. (D) Respective time dwell map, of sample preference group animal, where darker grey represents pixels with longer dwell time. Note that NE corner is the location with the longest dwell time. (E) Color-coded firing rate maps from six cells for the same sample animal from the preference group. Each cell is represented with four pairs of panels: top left pair is path trajectory (black lines) with spikes (blue dots) and firing map from the last training session; bottom left pair is path trajectory (black lines) with spikes (blue dots) and firing map from the probe; top right pair is path trajectory with spikes from the probe choice points only for passes towards the NE corner (green dots) and respective firing rate; bottom right pair is path trajectory with spikes from the probe choice points only for passes towards the SW corner (yellow dots) and respective firing rate. Note the difference for the maximum firing rate between NE and SW passes (the values are displayed in the rate map insets). Files dataset is available at Figshare public repository in Tsanov 2017 data / Continuous T-maze VTA folder https://figshare.com/s/5c5ba9b2811f3d7b7696.
(TIF)

S8 Fig Expression of iC++ AVV in the ventral tegmental area of TH::Cre rats. (A) YFP expression, tyrosine hydroxylase (TH) staining and their overlay in the VTA of TH::Cre rats injected with cre-inducible iC++ adeno-associated virus. (B) High-magnification confocal image shows confocal 3D view of a VTA neuron with YFP, TH and DAPI overlaid. (C) Images with a tetrode’s track showing the tip location of the recording electrode (marked with the white arrow). The last recorded neuron (denoted with the white asterisk) expresses YFP (upper image) and it is TH-positive (lower image). Files dataset is available at Figshare public repository in Tsanov 2016 data / iC++ immunohistology folder https://figshare.com/s/b86a9a111353ba04bd32.
(TIF)

S9 Fig Electrophysiological response of VTA neurons to blue light application in TH::Cre rats injected with iC++ AVV. (A) Firing rate of 78 recorded slow-spiking (<10Hz) neurons represented as percentage of pre-stimulation values. 45 cells showed no effect, 30 cells responded with significant inhibition of their firing rate, two-tailed independent *t*-test test, *t*(73) = 10.371, ***P < 0.001, n = 45 non-affected cells, and 3 cells responded with excitation: *t*(46) = -10.909, ***P < 0.001. Error bars, mean ± s.e.m. (B) Raster plot from 120 repetitions (above) and spike count of 120 repetitions (below) of two slow-spiking interneurons. Time 0 indicates the delivery of the first train of the stimulation protocol. (C) Raster plot from 120 repetitions (above) and spike count of 120 repetitions (below) of sample slow-spiking cell in VTA show the novelty-induced increase of their baseline spiking (right) compared to familiar well-habituated environment (left). A novelty-induced increase of the firing rate is an electrophysiological feature of VTA dopaminergic cells [1]. Files dataset is available at Figshare public repository in Tsanov 2016 data / iC++ electrophysiology folder https://figshare.com/s/b86a9a111353ba04bd32. 1. McNamara CG, Tejero-Cantero A, Trouche S, Campo-Urriza N, Dupret D. Dopaminergic neurons promote hippocampal reactivation and spatial memory persistence. Nat Neurosci. 2014;17(12):1658–60. 10.1038/nn.3843. 25326690; PubMed Central PMCID: PMC4241115.
(TIF)

S10 Fig VTA TH+ suppression evokes gradual place field remapping of place cells with shift of the averaged SPV and navigation preference. (A) Spatial firing rate maps of sample place cells recorded from each animal of the iC++group (n = 6) from baseline recording (left pair of panels), first (middle pair of panels) and second iC++ photoinhibition session (right pair of panels). Each pair of panels represents the animal trajectory with spikes (colored dots) (left) and a color-coded firing rate map (right). Field and rate remapping is evident in the place cells of animals 1, 3, 6. Field center of mass remaps in the place cell of animals 2 and 5, while rate remaps in the place cell of animal 4. (B) Spatial firing rate maps of sample place cells recorded from each animal of the control YFP group (n = 7). The color-coded firing rate maps are scaled to the cell’s maximum firing rate within a session. Red symbolizes the peak rate and blue represents no firing. Random rate remapping also occurs in control recordings and here is evident in the place cells of animals 2, 4 and 5. No filed remapping is observed in YFP controls. Files dataset is available at Figshare public repository in Tsanov 2016 data / Rectangular track folder https://figshare.com/s/b86a9a111353ba04bd32.
(TIF)

S11 Fig The suppression of ventral tegmental dopaminergic activity shifts the averaged SPV and evokes navigation preference. (A) Comparison of the averaged SPV in degrees between the baseline (left bar), first session (middle bar) and second session photoinhibition (right bar) for the with iC++ group of rats, n = 6 rats, paired *t*-test test, day 1: *t*(5) = -2.935, *P = 0.032; day 2: *t*(5) = -2.169, *P* = 0.082. (B) Comparison of the SW/NE passes ratio between the baseline (left), first (middle) and second photoinhibition session (right) for the iC++ group of rats, n = 6, paired *t*-test test, day 1: *t*(5) = 2.679, *P = 0.044; day 2: *t*(5) = 2.602, *P = 0.048. (C) Comparison of the SW/NE passes ratio between the baseline (left), first (middle) and second control light delivery session (right) for the YFP group of rats n = 7, paired *t*-test test, day 1: *t*(6) = 0. 562, *P* = 0.594; day 2: *t*(6) = -0.151, *P* = 0.885. Error bars, mean ± s.e.m. Files dataset is available at Figshare public repository in Tsanov 2016 data / Rectangular track folder https://figshare.com/s/b86a9a111353ba04bd32.
(TIF)

S12 Fig Forced navigation during navigation across rectangular-shaped linear track. Left: biased navigation trajectory of a sample iC++ group animal during recording session with laser application (marked with a blue dashed square) in the north and east arms of the track. The experimenter actively guided the animal towards the east or north sections of the track (marked with red arrows) for 50% of the recording session. The access towards the SW corner was repeatedly restricted, which led to SW/NE ratio of 0.51. Right: weighted SPV of the spikes (colored dots) from the place cells (represented by different colors) recorded during same session with value of 42.2°. The forced navigation technique was chosen instead of compartmental obstruction for particular section of the maze. The compartmentalization of recording arena evokes remapping of place fields [1]. Files dataset is available at Figshare public repository in Tsanov 2017 data /Rectangular track forced navigation folder https://figshare.com/s/5c5ba9b2811f3d7b7696. 1. O'Keefe J, Burgess N. Geometric determinants of the place fields of hippocampal neurons. Nature. 1996;381(6581):425–8. 8632799.
(TIF)

S13 Fig Optogenetic excitation of VTA dopaminergic neurons augments place cell spiking. (A) Raster plot from 40 repetitions (above) and spike count of 120 repetitions (below) of two place cells. (B) Intra-field (left graph) and extra-field (right graph) firing rate of 22 place cells 100 ms after the onset of the stimulation protocol expressed as percentage of the pre-stimulation values, for control (left) and photostimulation (middle). The right dots show the firing rate (% pre-stimulation) for 250 ms after photostimulation onset. Paired *t*-test test: for intra-field control vs 100ms ChR2 *t*(21) = -4.344, ***P < 0.001. Error bars, mean ± s.e.m. Paired *t*-test test: for extra-field control vs 100ms ChR2 *t*(21) = 0.111, *P* = 0.913; for extra-field control vs 250ms ChR2 *t*(21) = -0.871, *P* = 0.394. Error bars, mean ± s.e.m. (C) Sample place field map of CA1 pyramidal neuron after exploration of open square arena. The map represents animal’s trajectory with spikes as coloured dots. The intra-field spikes are marked in purple, while the extra-field spikes are marked in red. The image on the right represents the corresponding firing rate map for the same place cell. Files dataset is available at Figshare public repository in Tsanov 2016 data / E123T electrophysiology folder https://figshare.com/s/b86a9a111353ba04bd32.
(TIF)

S14 Fig Optogenetic excitation of VTA dopaminergic neurons suppresses postsynaptic slow-spiking interneurons. (A) Raster plot from 40 repetitions (above) and spike count of 120 repetitions (below) of two slow-spiking interneurons. (B) Spike train cross-correlogram between the interneurons and the place cells shown in (SA Fig). (C) Summary histogram of the spiking cross-correlation peak values for recorded place cell-interneuron pairs (n = 22). Files dataset is available at Figshare public repository in Tsanov 2016 data / Open arena regular stimulation folder https://figshare.com/s/b86a9a111353ba04bd32.
(TIF)

S15 Fig Place field’s center of mass is not affected in controls, but only in ChR2 animals. (A) Firing map of a sample place cell from the first (above) and the second (below) baseline recordings, and (B) firing map of the same sample place cell from the first (above) and second (below) control YFP light application. Top left panels represent the animal trajectory with spikes (red dots), top middle panels show the coordinates of the laser application (blue dashed lines) and the applied light pulses (red dots); and top right panels show color-coded bhatt overlap between the distribution of the spikes and the applied light pulses. Bottom images show 3D color-coded firing rate maps of the place cells. (C) Firing map of a sample place cell from the first (above) and the second (below) baseline recordings and (D) the spikes from the first (above) and second (below) ChR2 photostimulation session. Top left panels represent the animal trajectory with spikes (red dots), top middle panels show the coordinates of the laser application (blue dashed lines) and the applied light pulses (red dots); and top right panels show color-coded bhatt overlap between the distribution of the spikes and the applied light pulses. Bottom images show 3D color-coded firing rate maps of the place cells. Files dataset is available at Figshare public repository in Tsanov 2016 data / Open arena spatial stimulation folder https://figshare.com/s/b86a9a111353ba04bd32 and Tsanov 2016 data addition folder https://figshare.com/s/b2c6e7a8a0417820720c.
(TIF)

S16 Fig Dopaminergic photostimulation does not evoke consistent alterations of hippocampal place filed properties. (A) Comparison of the place field center rate (left), mean filed rate (middle) and spatial coherence (right) between baselines and light delivery sessions of the control YFP group (n = 16 cells) and ChR2 group of rats (n = 18 cells). (B) Comparison of the field size (left), spatial information content (middle) and raw Bhattacharyya distance (right) between baselines and light delivery sessions of the control YFP group (n = 16 cells) and ChR2 group of rats (n = 18 cells). The place field size ratio of the baseline over the second baseline underwent non-significant decrease for the ChR2 group, two-tailed independent *t*-test test, *t*(31) = -1.886, p = 0.069. Concurrently, the spatial information ratio of the baseline over the second baseline underwent non-significant increase for the ChR2 group, *t*(31) = 1.895, p = 0.068. The raw Bhattacharyya distance (bhatt) was calculated for the non-smoothed rate maps for the ChR2 group (n = 18) and control YFP group (n = 16), two-tailed independent *t*-test test, baseline 2 session, *t*(31) = 3.547, **P = 0.001; light delivery 2 session, *t*(31) = 2.539, **P = 0.016. Error bars, mean ± s.e.m. The values are represented as ratios of the measured values from first baseline session over the values of the subsequent light delivery, second baseline and second light delivery session. Tsanov 2016 data / Open arena spatial stimulation folder https://figshare.com/s/b86a9a111353ba04bd32 and Tsanov 2016 data addition folder https://figshare.com/s/b2c6e7a8a0417820720c.
(TIF)

S17 Fig Electrical stimulation of the medial forebrain bundle. Inset atlas schematic shows the location of chronically-implanted electrodes in the medial forebrain bundle (mfb). A sample histology shows the tip of the bipolar concentric electrode (marked with white arrow). For verification of electrode location electrolytic lesion was induced by high-current intensity (0.5 mA *current* applied for a period of 10 s) after the completion of the experiments under non-recovery isoflurane anaesthesia. Files dataset is available at Figshare public repository in Tsanov 2016 data / Open arena spatial stimulation folder https://figshare.com/s/b86a9a111353ba04bd32.
(TIF)

S18 Fig MFB stimulation evokes relocation of place field’s center of mass. (TIF)

S19 Fig MFB stimulation shifts place field’s center of mass (COM) in direction of the applied stimulation pulses. (A) Pearson’s correlation between bhatt and ΔCOM for the MFB group. (B) The difference of the center of mass (ΔCOM) between the baseline and the following first light delivery and MFB stimulation; baseline and second baseline; baseline and second light delivery and MFB stimulation session, two-tailed independent *t*-test test; *t*(32) = 3.470, **P = 0.002 for YFP control (n = 16 cells) and MFB stimulation (n = 18 cells) groups. Error bars, mean ± s.e.m. (C) Ratio of Bhattacharyya distance (bhatt) of the baseline values over the following first light delivery and MFB stimulation; baseline over second baseline, two-tailed independent *t*-test test, *t*(32) = 2.041, *P = 0.049; baseline over second light delivery and MFB stimulation session, *t*(32) = 3.040, **P = 0.005, for YFP control (n = 16) and MFB stimulation (n = 18) groups. Error bars, mean ± s.e.m. (D) Comparison of the place field peak rate (left), mean field rate (middle) and spatial coherence (right) between baselines and light delivery sessions of the control YFP and MFB group of rats. (E) Comparison of the place field size (left) and spatial information (middle) between baselines and light delivery sessions of the control YFP and MFB group of rats. Right: raw bhatt calculated for the non-smoothed rate map for the MFB group (n = 18) and control YFP group (n = 16), two-tailed independent *t*-test test, baseline 2 session, *t*(32) = 2.415, *P = 0.022; light delivery 2 and MFB 2 stimulation *t*(32) = 3.256, **P = 0.003. Error bars, mean ± s.e.m. Files dataset is available at Figshare public repository in Tsanov 2016 data / Open arena spatial stimulation folder https://figshare.com/s/b86a9a111353ba04bd32.
(TIF)

S1 Table Number of passes in the probe session of continuous T-maze task. (DOCX)

S2 Table Spatial filed configuration (degrees) for all cells in the probe session of T-maze task. (DOCX)

S3 Table Center of mass angle (degrees) for all cells in the probe session of T-maze task. (DOCX)

S4 Table Spatial population vector (degrees) in the probe session of continuous T-maze task. (DOCX)

S5 Table Number of passes and SPV in rectangular-shaped linear task for iC++ rats. (DOCX)

S6 Table Number of passes and SPV in rectangular-shaped linear task for YFP rats. (DOCX)

S7 Table Post-photostimulation firing rate (% pre-stimulation). (DOCX)

S8 Table Post-stimulation firing rate (% pre-stimulation) for intra- vs extra place field spikes. (DOCX)

S1 Data Place field properties of place cells in open arena after hippocampal VTA fibers-photostimulation. (XLSX)

S2 Data Place field properties of place cells in open arena after control light delivery. (XLSX)

S3 Data Place field properties of place cells in open arena after VTA-photostimulation. (XLSX)

S4 Data Place field properties of place cells in open arena after MFB-stimulation. (XLSX)

## Abbreviations

AAV: adeno-associated virus
bhatt: Bhattacharyya distance metric
ChR2: channelrhodopsin 2
COM: center of mass
fMRI: functional magnetic resonance imaging
iC++: light-activated chloride channel
MFB: medial forebrain bundle
NE: northeast
NO: nitric oxid
NPY: neuropeptide Y
SFC: spatial field configuration
SPV: spatial population vector
SW: southwest
TH+: tyrosine hydroxylase positive
VTA: ventral tegmental area
YFP: yellow fluorescent protein
