# Supplementary figures and images for: Place field assembly distribution encodes preferred locations
Source: PLoS Biol. 2017 Sep 12;15(9):e2002365. doi: 10.1371/journal.pbio.2002365 (PMC5609775; doi:10.1371/journal.pbio.2002365)

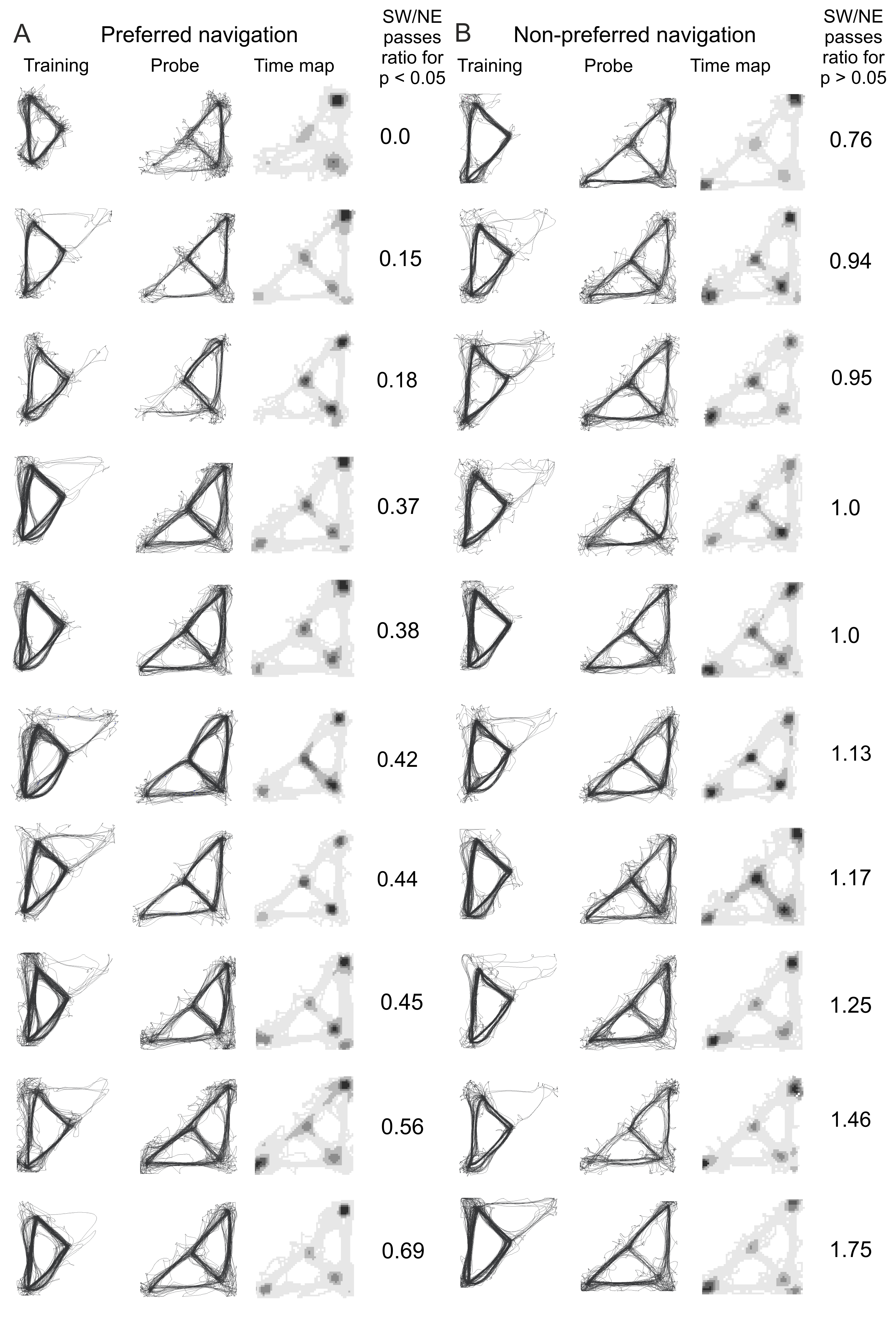

Supplement: S1 Fig — (A) Navigation trajectory from the preference group of animals (n = 10) during the last training session (left panels) and during the probe (middle panels). The right panels show the respective time rate maps, where darker grey represents pixels with longer dwell time. The numbers on the right show the SW/NE passes ratio for each animal. They are presented in order from the lowest to the highest value. (B) Navigation trajectory from the non-preference group of animals (n = 10) during the last training session (left panels) and during the probe (middle panels). The right panels show the respective time rate maps, where darker grey represents pixels with longer dwell time. The numbers on the right show the SW/NE passes ratio for each animal. They are presented in order from the lowest to the highest value. Files dataset is available at Figshare public repository in Tsanov 2016 data / Continuous T-maze folder https://figshare.com/s/b86a9a111353ba04bd32 and Tsanov 2017 data / Continuous T-maze CA1 folder https://figshare.com/s/5c5ba9b2811f3d7b7696. (TIF) [file pbio.2002365.s001.tif]

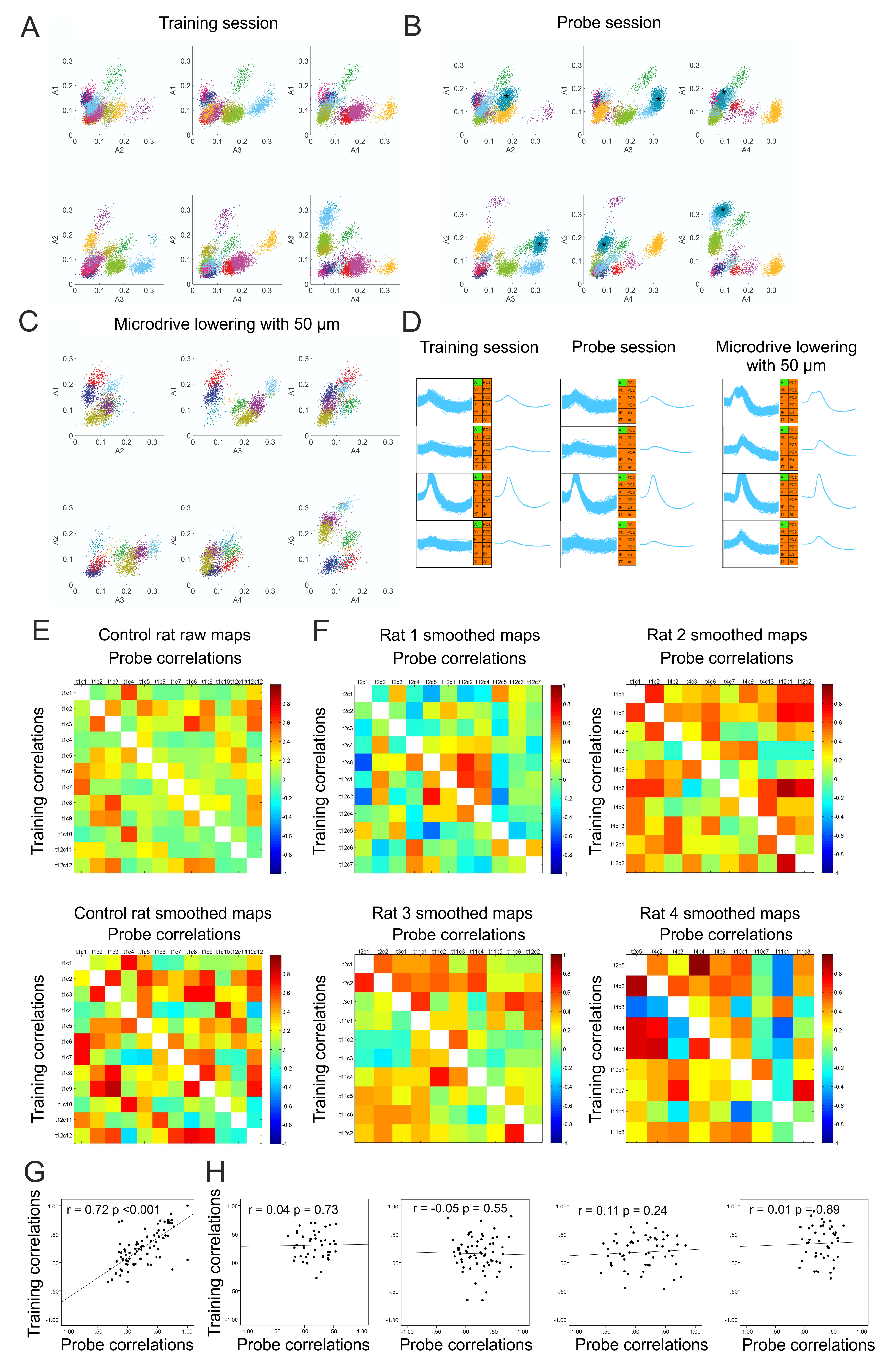

Supplement: S2 Fig — (A) Scatterplot, showing the signals from multiple units recorded between each pair of electrodes on a given tetrode from sample training session. The color-coded clusters represent the spikes from each unit at the scatterplot. The spike sorting technique compares the amplitude of the recorded signal between each electrode tip (A1, A2, A3, A4). The maximal waveform amplitude of each unit is measured at different electrode. Each waveform is represented by different spike shape, measured by the peak-trough amplitude of the spike. (B) Spike clusters of multiple cells from probe session. To confirm the stability of the signal after multiple recordings across consecutive days the spike waveform and the position of the spike clusters in the 6 electrode-pair scatterplots were examined between recording sessions. The stability of the waveform is evaluated by the position of each spike cluster on the two-dimensional comparison by the peak-trough amplitude on one electrode against the peak-trough amplitude on another. The probe session was characterized with activation of a new place cell (the new spike cluster is marked with black asterisk). (C) Displacement of the tetrodes results in simultaneous change of the clusters location across all electrode tip pairs. The scaterplot shows the rearrangement of the spike clusters after 50 μm lowering of the implanted microdrive. Note the change of the clusters locations compared to the training (A) and probe sessions (B) per each electrode pair. (D) The stability of the spike signal between the training (left) and probe session (middle) is evaluated by the peak-trough amplitude of the spike and the the time of occurrence of maximum and minimum spike voltages for all four electrode channels. The highest amplitude of the recorded signal for the blue spikes is expressed at the third electrode channel. Note the change of the spike shape at the third electrode after 50 μm microdrive lowering (right). Note also the increase of the spike amp [file pbio.2002365.s002.tif]

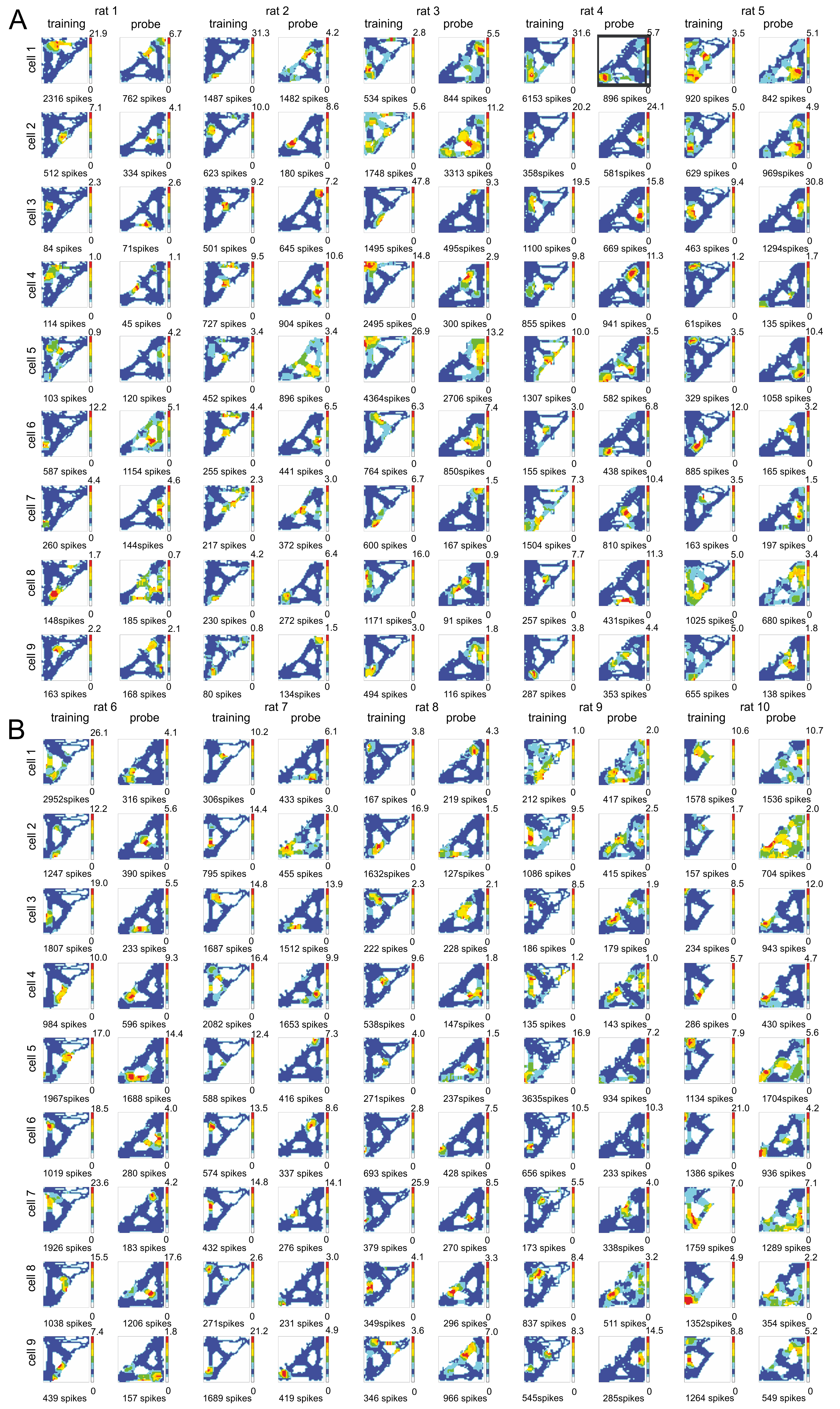

Supplement: S3 Fig — (A) Color-coded firing rate maps of 45 sample place cells recorded during the training (left) and probe session (right) from 5 rats from the preference group (rats 1–5). (B) Color-coded firing rate maps of 45 sample place cells recorded during the training (left) and probe session (right) from 5 rats from the non-preference group (rats 6–10). The number of recorded spikes of each place cell is shown below the maps. Files dataset is available at Figshare public repository in Tsanov 2016 data / Continuous T-maze folder https://figshare.com/s/b86a9a111353ba04bd32 and Tsanov 2017 data / Continuous T-maze CA1 folder https://figshare.com/s/5c5ba9b2811f3d7b7696. (TIF) [file pbio.2002365.s003.tif]

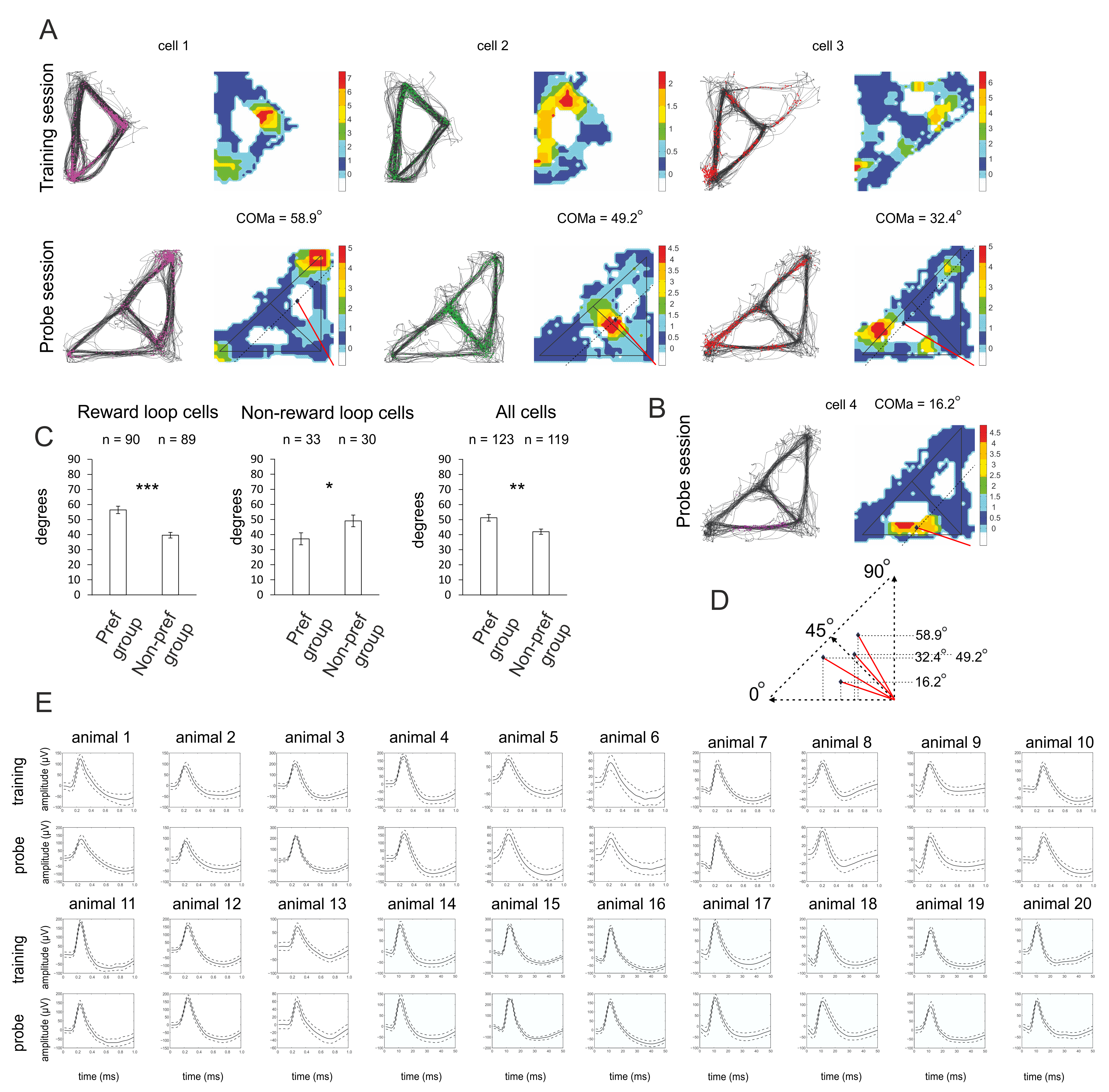

Supplement: S4 Fig — (A) Three sample place cells recorded from the reward loop of the training sessions (reward loop cells) from three representative animals. Upper panels show the animal trajectory with spikes, marked with colored dots and their color-coded firing rate map (right) from the last training session. Lower panels show the animal trajectory with spikes (left) and their place fields’ color-coded firing rate maps (right) from the probe session. The center of mass (COM) is indicated with black mark at the end of the red line. The straight red line denotes the direction (in degrees) of center of mass angle (COMa) between SW at 0°, NE at 90° with midline at 45°. (B) Sample place cell which was absent from the reward loop of the training sessions (non-reward loop cells). (C) COM angle values from the preference and non-preference groups are reported for the reward loop cells. Two-tailed independent t-test test, left: for the non-reward loop cells n = 90 cells (preference group), n = 89 cells (non-preference group), t(177) = 5.477, ***P < 0.001; middle: for the non-reward loop cells, n = 33 and n = 30, respectively, t(61) = -2.137, *P = 0.037; and left: for all cells, n = 123 and n = 119, respectively, t(240) = 3.274, **P = 0.001. Error bars, mean ± s.e.m. (D) Schematic representation of the COM location (indicated with black mark at the end of the red lines) for the four cells from (A-B). The red line connects COM with the starting coordinate to form an angle with the horizontal dashed line indicating 0 degrees. (E) Spike waveform of a sample place cells from each animal (n = 20), recorded from the last training session (above) and from the probe (below). For each waveform, the solid line is the average waveform shape, and the dashed lines show the 1 SD confidence intervals. The y-axis scale denotes the amplitude of the action potential in microvolts (negativity is up), and the dotted horizontal line through 0 denotes the baseline potential. The length of the x-axis represents 1 [file pbio.2002365.s004.tif]

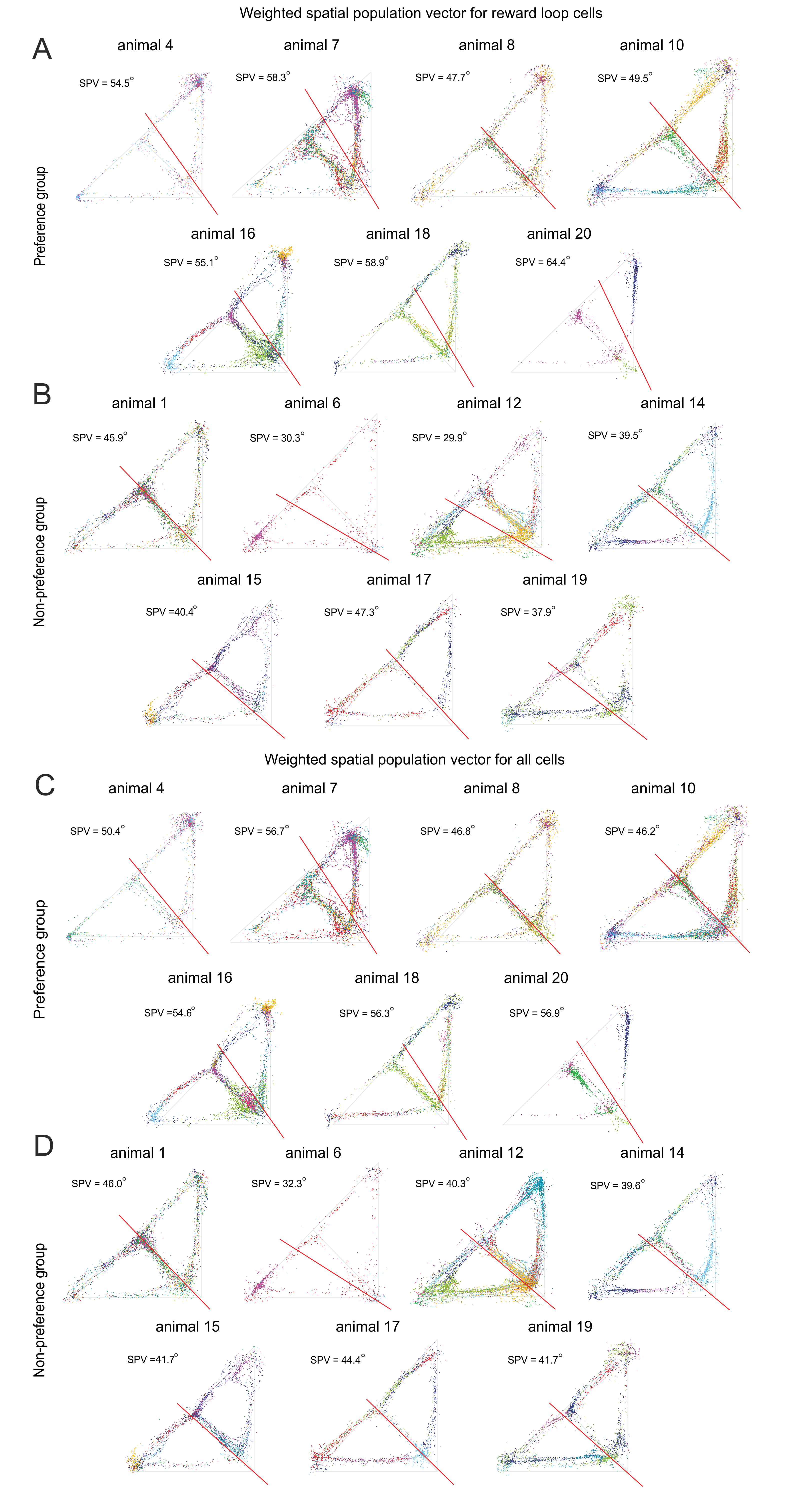

Supplement: S5 Fig — (A) Spatial distribution of the spikes (colored dots) from the reward loop cells (represented by different colors) recorded from seven preference group animals and (B) from seven non-preference group animals. The straight red line denotes the weighted spatial population vector (SPV in degrees) between SW at 0° and NE at 90°. (C) Spatial distribution of the spikes (colored dots) from the all cells (represented by different colors), including reward and non-reward loop cells recorded from the preference and (D) non-preference group animals, respectively. Files dataset is available at Figshare public repository in Tsanov 2016 data / Continuous T-maze folder https://figshare.com/s/b86a9a111353ba04bd32 and Tsanov 2017 data / Continuous T-maze CA1 folder https://figshare.com/s/5c5ba9b2811f3d7b7696. (TIF) [file pbio.2002365.s005.tif]

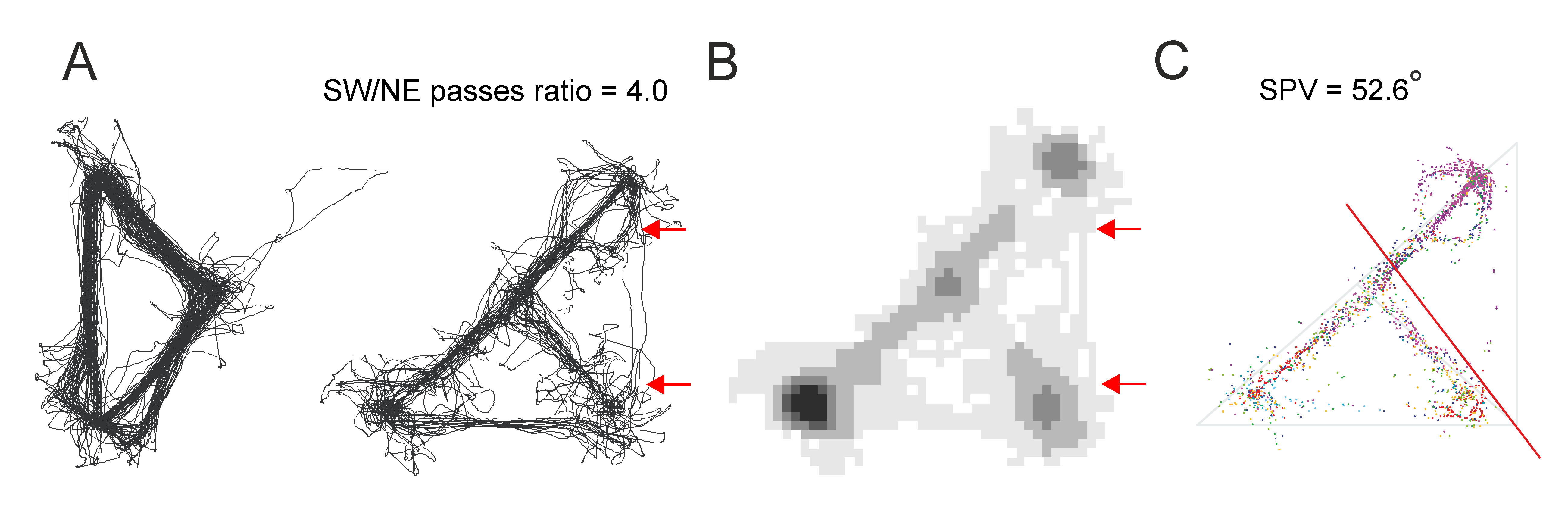

Supplement: S6 Fig — (A) Biased navigation trajectory from the sample animal during the last training session (left panel) and during the probe (right panel). After the animal explored the east track of the maze in each direction, the access to this arm was restricted. If the rat was approaching towards the east track either from the north or from the south side (marked with red arrows) the experimenter actively guided the animal towards the opposite direction. As a result, the rat navigated four times more often towards SW compared towards NE corner (the SW/NE passes ratio = 4) from the choice points. The forced navigation technique was chosen instead of compartmental obstruction for particular section of the maze. The compartmentalization of recording arena evokes remapping of place fields [1]. (B) Respective time rate map, where darker grey represents pixels with longer dwell time. Note that SW corner is the location with the longest dwell time. (C) Weighted spatial distribution of the spikes (colored dots) from the all cells (represented by different colors) recorded, with SPV value of 52.6°. The weighted SPV value for the spikes only from the reward loop cells was 51.3°. Files dataset is available at Figshare public repository in Tsanov 2017 data / Continuous T-maze forced navigation folder https://figshare.com/s/5c5ba9b2811f3d7b7696. 1. O'Keefe J, Burgess N. Geometric determinants of the place fields of hippocampal neurons. Nature. 1996;381(6581):425–8. 8632799. (TIF) [file pbio.2002365.s006.tif]

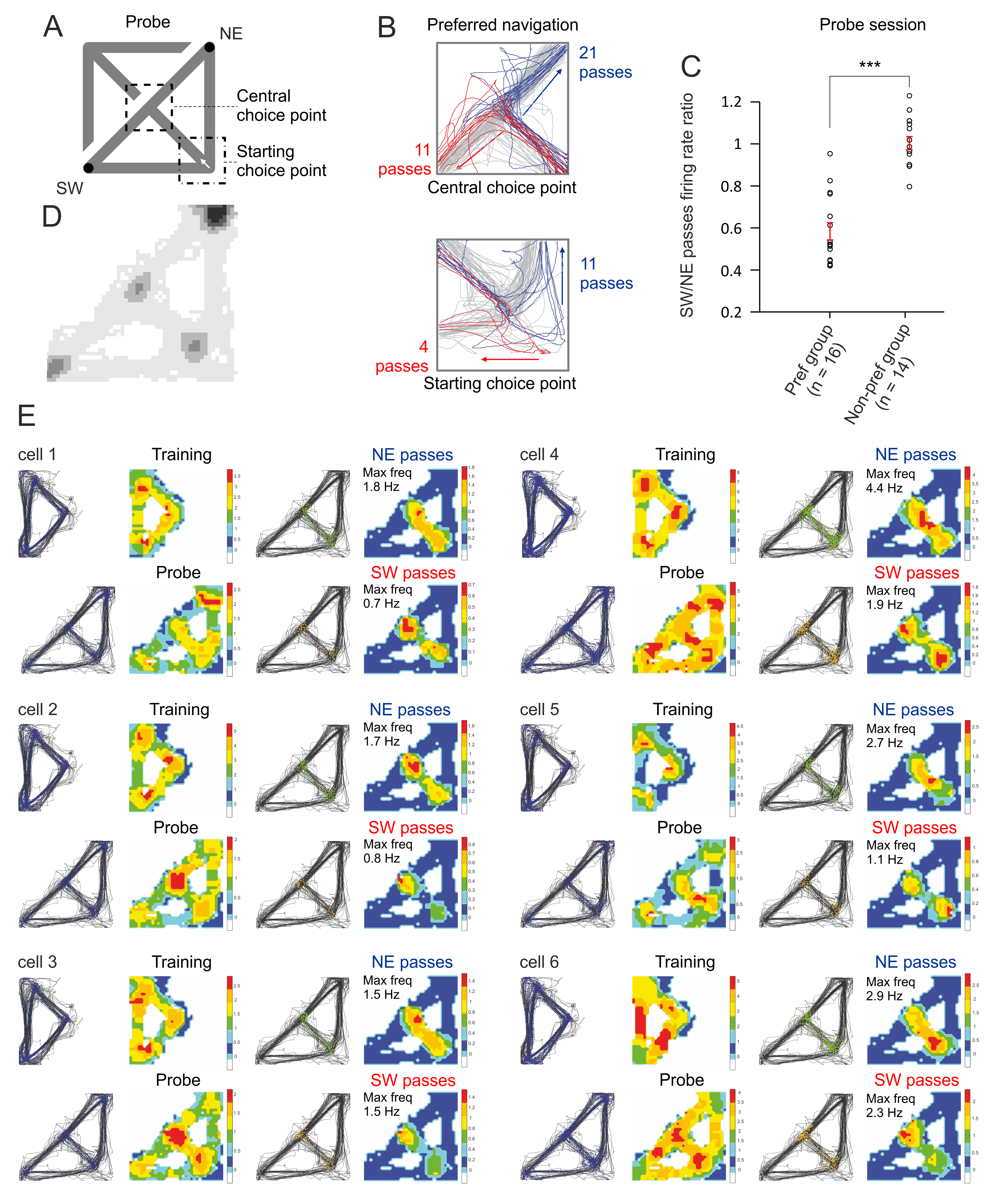

Supplement: S7 Fig — (A) The behavioral set-up of the continuous T-maze for the ventral tegmental recordings was the same as for the hippocampal recordings. In this case we evaluated the direction of the passes from the starting choice point (marked with a white arrow) and the central choice point as well as the firing rate of the recorded neurons for each direction (towards SW versus towards NE direction). (B) Representation of the passes from preference group rat towards the SW corner (in red) and towards NE corner (in blue) from the central choice point (above) and from the starting choice point (below). (C) Comparison of the firing rate ratio of southwest (SW) to northeast (NE) passes. Error bars, mean ± s.e.m., n = 16 cells from 3 rats (preference group), n = 14 cells from 4 rats (non-preference group), two-tailed independent t-test test, t(28) = -6.737, ***P < 0.001. (D) Respective time dwell map, of sample preference group animal, where darker grey represents pixels with longer dwell time. Note that NE corner is the location with the longest dwell time. (E) Color-coded firing rate maps from six cells for the same sample animal from the preference group. Each cell is represented with four pairs of panels: top left pair is path trajectory (black lines) with spikes (blue dots) and firing map from the last training session; bottom left pair is path trajectory (black lines) with spikes (blue dots) and firing map from the probe; top right pair is path trajectory with spikes from the probe choice points only for passes towards the NE corner (green dots) and respective firing rate; bottom right pair is path trajectory with spikes from the probe choice points only for passes towards the SW corner (yellow dots) and respective firing rate. Note the difference for the maximum firing rate between NE and SW passes (the values are displayed in the rate map insets). Files dataset is available at Figshare public repository in Tsanov 2017 data / Continuous T-maze VTA folder https://figshare.com/s/ [file pbio.2002365.s007.tif]

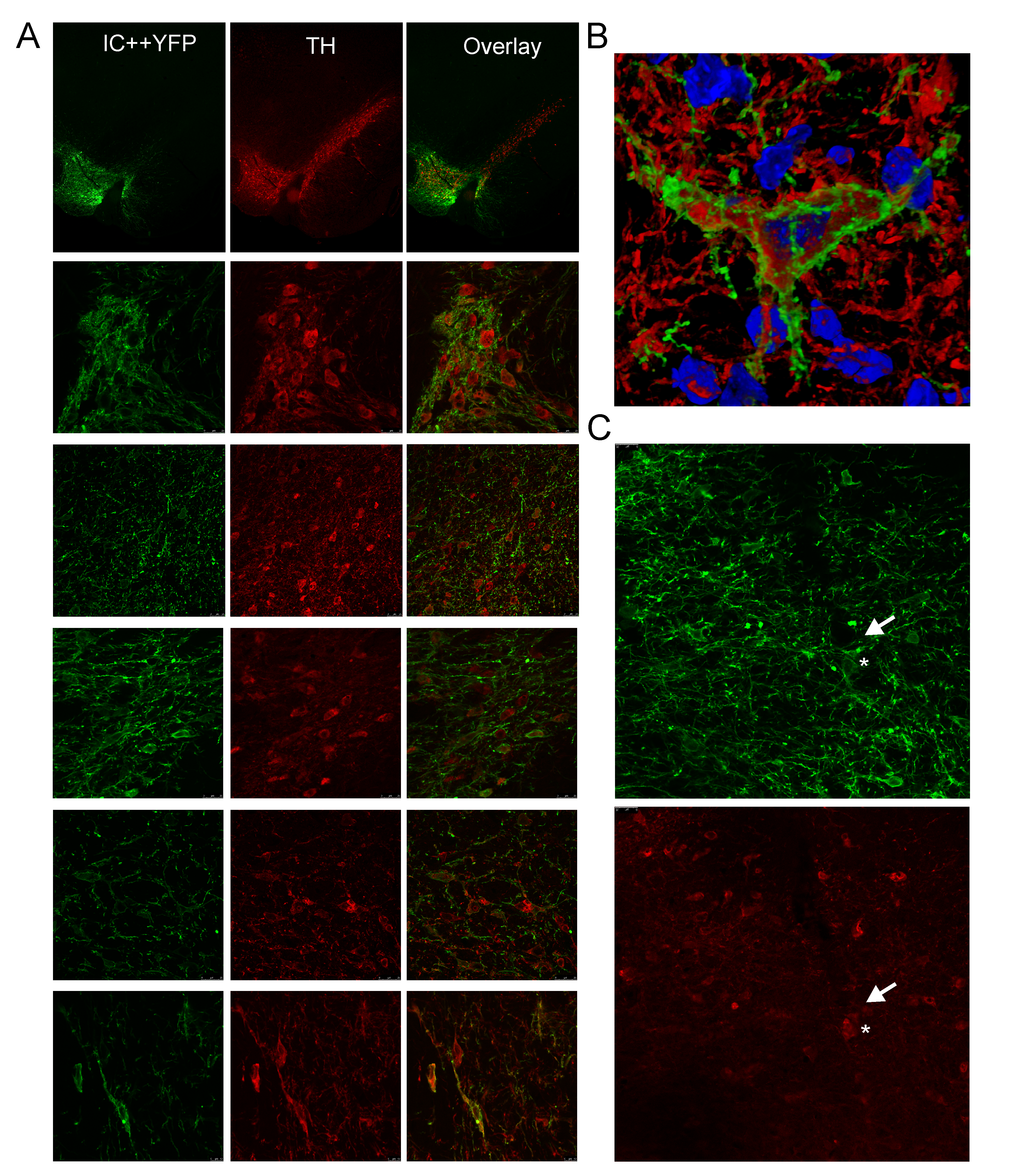

Supplement: S8 Fig — (A) YFP expression, tyrosine hydroxylase (TH) staining and their overlay in the VTA of TH::Cre rats injected with cre-inducible iC++ adeno-associated virus. (B) High-magnification confocal image shows confocal 3D view of a VTA neuron with YFP, TH and DAPI overlaid. (C) Images with a tetrode’s track showing the tip location of the recording electrode (marked with the white arrow). The last recorded neuron (denoted with the white asterisk) expresses YFP (upper image) and it is TH-positive (lower image). Files dataset is available at Figshare public repository in Tsanov 2016 data / iC++ immunohistology folder https://figshare.com/s/b86a9a111353ba04bd32. (TIF) [file pbio.2002365.s008.tif]

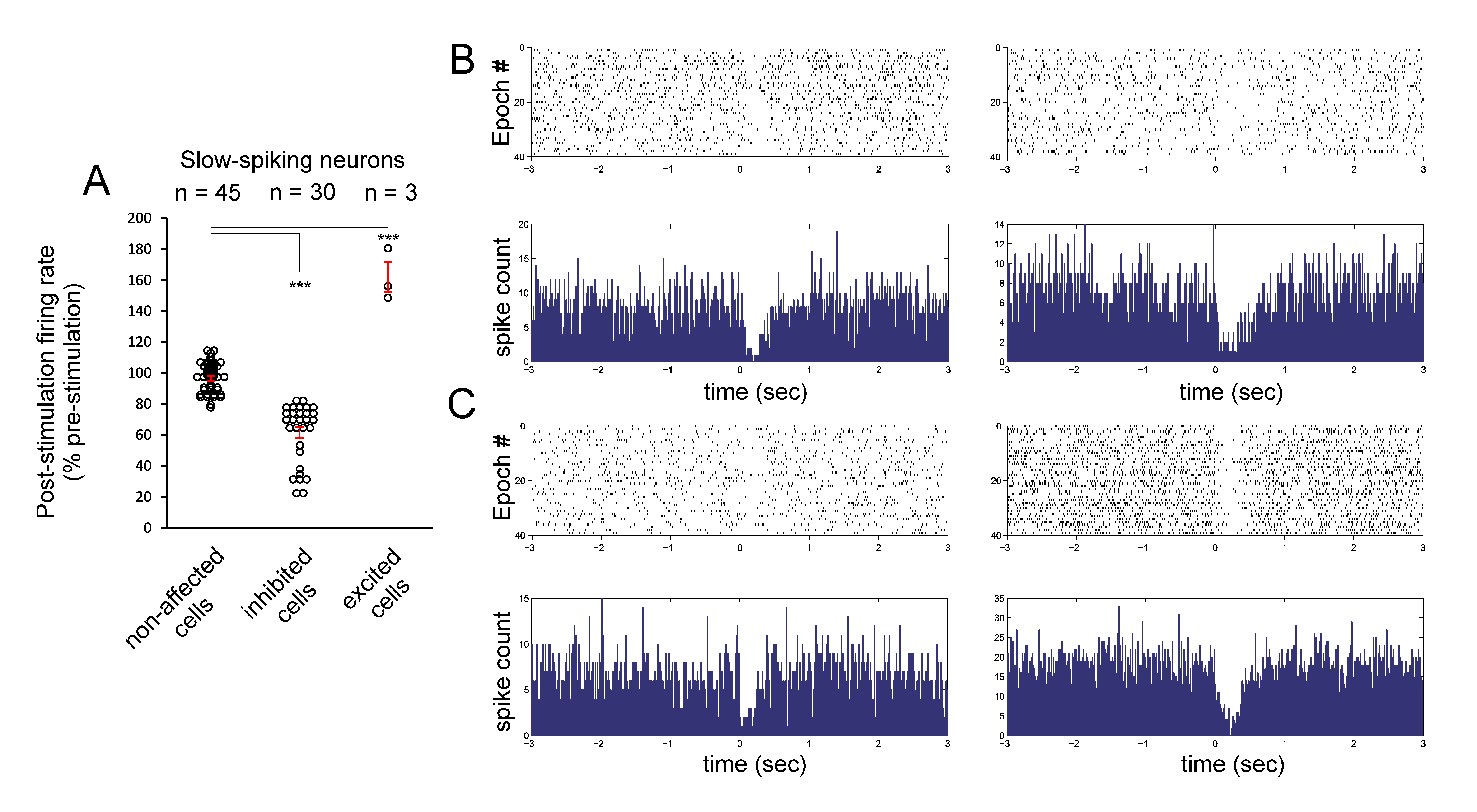

Supplement: S9 Fig — (A) Firing rate of 78 recorded slow-spiking (<10Hz) neurons represented as percentage of pre-stimulation values. 45 cells showed no effect, 30 cells responded with significant inhibition of their firing rate, two-tailed independent t-test test, t(73) = 10.371, ***P < 0.001, n = 45 non-affected cells, and 3 cells responded with excitation: t(46) = -10.909, ***P < 0.001. Error bars, mean ± s.e.m. (B) Raster plot from 120 repetitions (above) and spike count of 120 repetitions (below) of two slow-spiking interneurons. Time 0 indicates the delivery of the first train of the stimulation protocol. (C) Raster plot from 120 repetitions (above) and spike count of 120 repetitions (below) of sample slow-spiking cell in VTA show the novelty-induced increase of their baseline spiking (right) compared to familiar well-habituated environment (left). A novelty-induced increase of the firing rate is an electrophysiological feature of VTA dopaminergic cells [1]. Files dataset is available at Figshare public repository in Tsanov 2016 data / iC++ electrophysiology folder https://figshare.com/s/b86a9a111353ba04bd32. 1. McNamara CG, Tejero-Cantero A, Trouche S, Campo-Urriza N, Dupret D. Dopaminergic neurons promote hippocampal reactivation and spatial memory persistence. Nat Neurosci. 2014;17(12):1658–60. 10.1038/nn.3843. 25326690; PubMed Central PMCID: PMC4241115. (TIF) [file pbio.2002365.s009.tif]

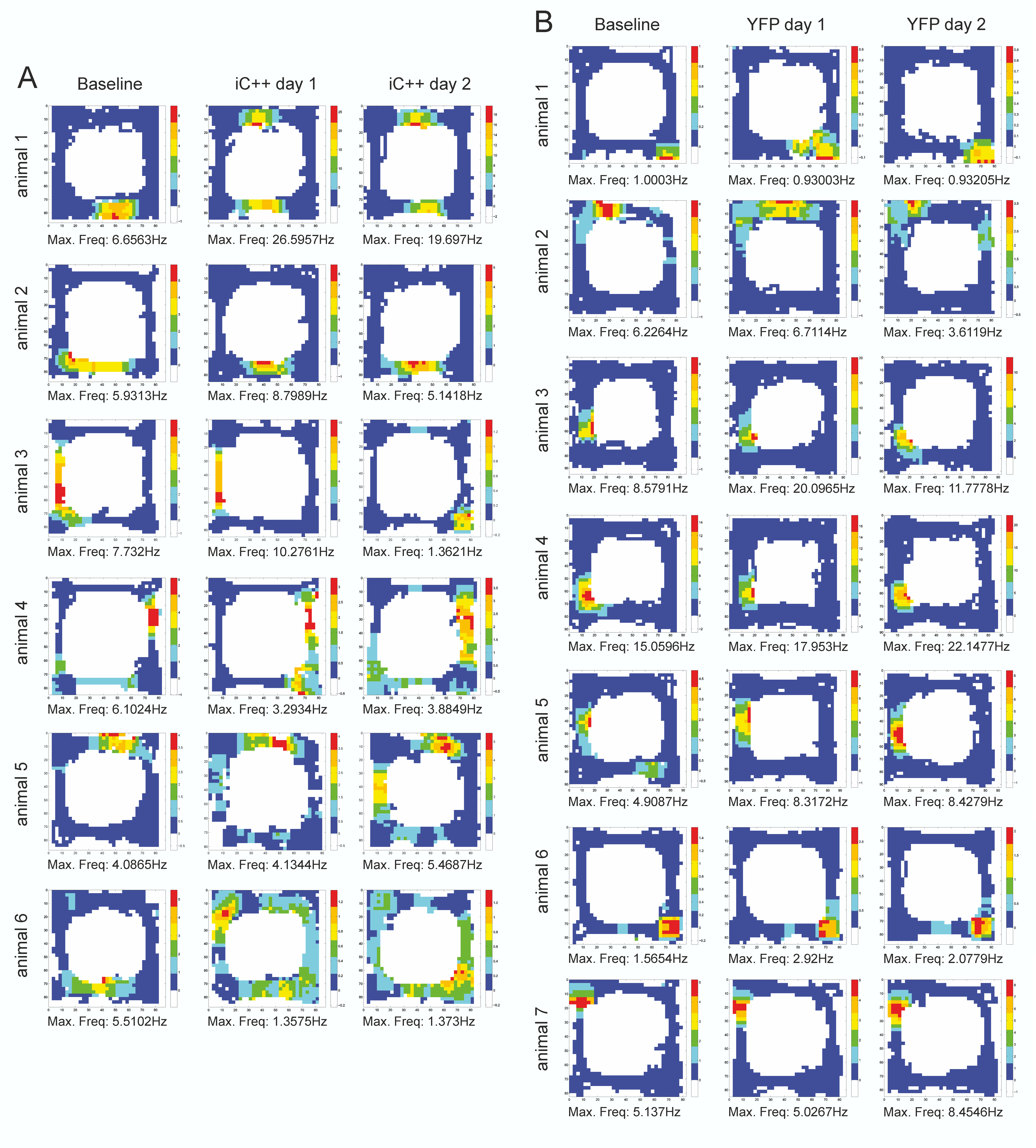

Supplement: S10 Fig — (A) Spatial firing rate maps of sample place cells recorded from each animal of the iC++group (n = 6) from baseline recording (left pair of panels), first (middle pair of panels) and second iC++ photoinhibition session (right pair of panels). Each pair of panels represents the animal trajectory with spikes (colored dots) (left) and a color-coded firing rate map (right). Field and rate remapping is evident in the place cells of animals 1, 3, 6. Field center of mass remaps in the place cell of animals 2 and 5, while rate remaps in the place cell of animal 4. (B) Spatial firing rate maps of sample place cells recorded from each animal of the control YFP group (n = 7). The color-coded firing rate maps are scaled to the cell’s maximum firing rate within a session. Red symbolizes the peak rate and blue represents no firing. Random rate remapping also occurs in control recordings and here is evident in the place cells of animals 2, 4 and 5. No filed remapping is observed in YFP controls. Files dataset is available at Figshare public repository in Tsanov 2016 data / Rectangular track folder https://figshare.com/s/b86a9a111353ba04bd32. (TIF) [file pbio.2002365.s010.tif]

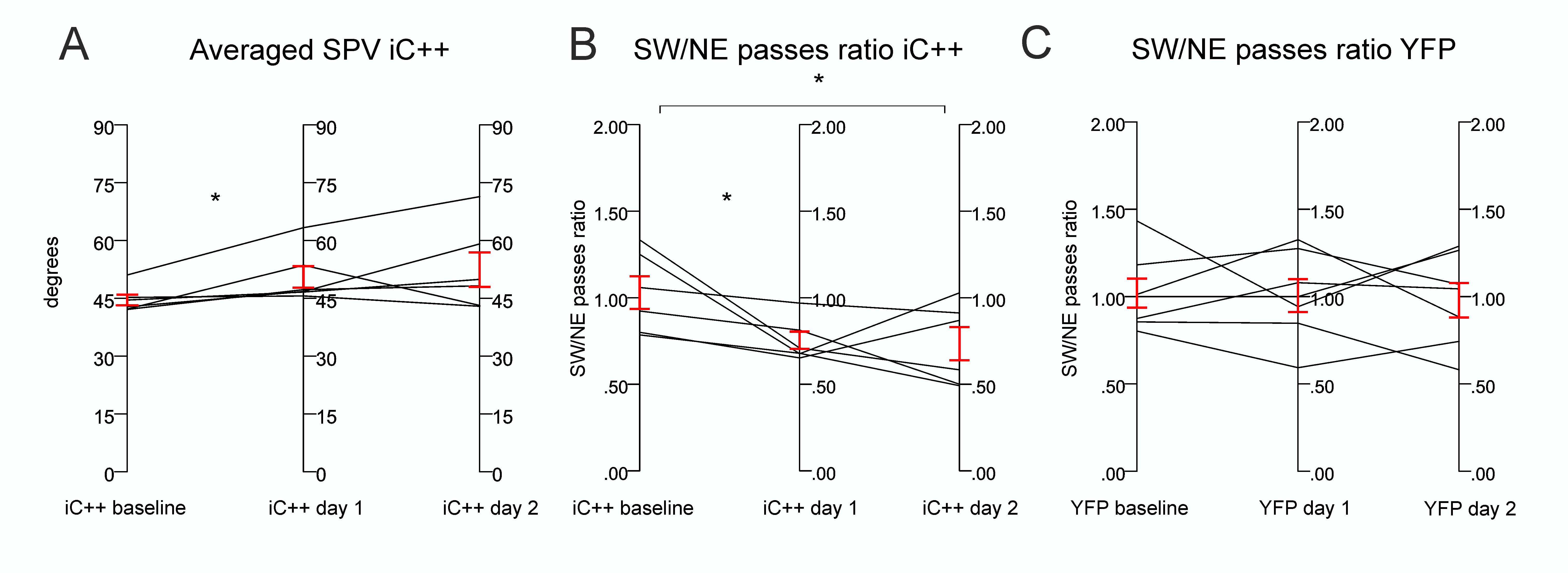

Supplement: S11 Fig — (A) Comparison of the averaged SPV in degrees between the baseline (left bar), first session (middle bar) and second session photoinhibition (right bar) for the with iC++ group of rats, n = 6 rats, paired t-test test, day 1: t(5) = -2.935, *P = 0.032; day 2: t(5) = -2.169, P = 0.082. (B) Comparison of the SW/NE passes ratio between the baseline (left), first (middle) and second photoinhibition session (right) for the iC++ group of rats, n = 6, paired t-test test, day 1: t(5) = 2.679, *P = 0.044; day 2: t(5) = 2.602, *P = 0.048. (C) Comparison of the SW/NE passes ratio between the baseline (left), first (middle) and second control light delivery session (right) for the YFP group of rats n = 7, paired t-test test, day 1: t(6) = 0. 562, P = 0.594; day 2: t(6) = -0.151, P = 0.885. Error bars, mean ± s.e.m. Files dataset is available at Figshare public repository in Tsanov 2016 data / Rectangular track folder https://figshare.com/s/b86a9a111353ba04bd32. (TIF) [file pbio.2002365.s011.tif]

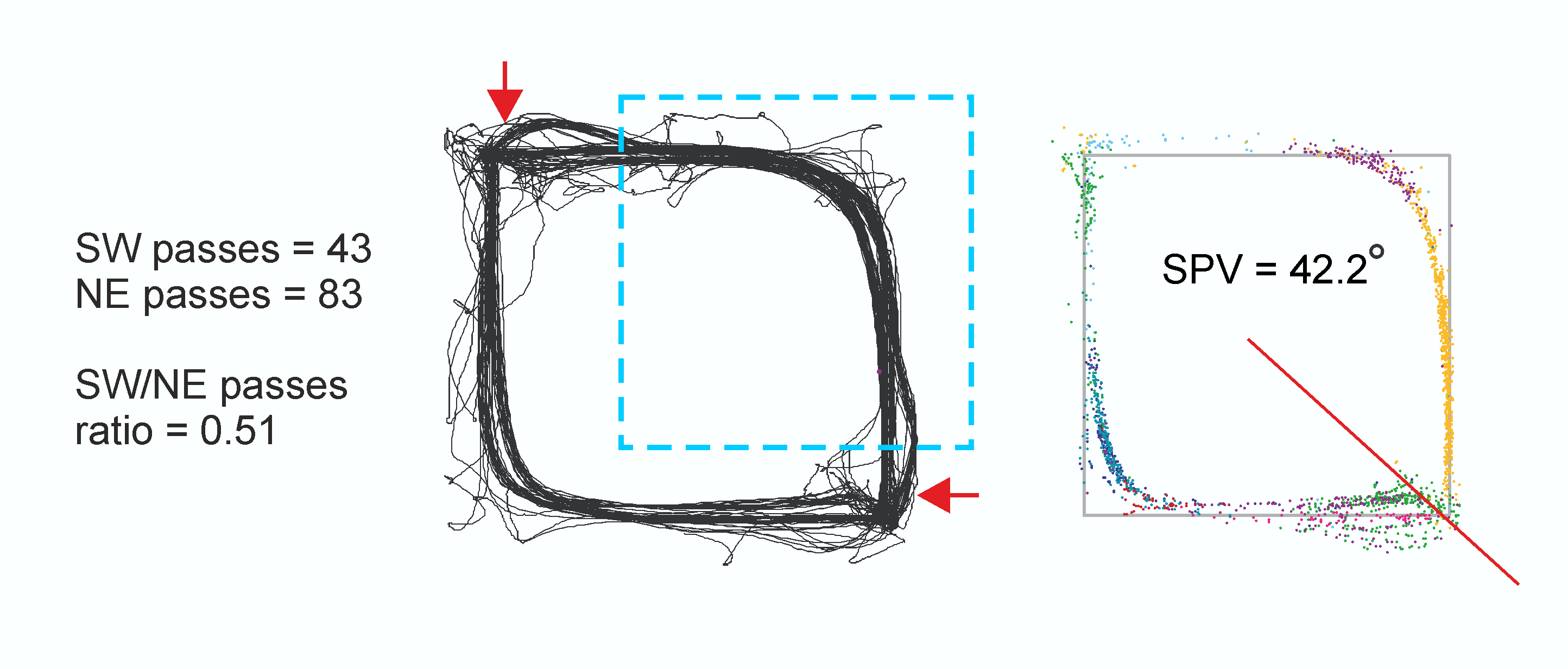

Supplement: S12 Fig — Left: biased navigation trajectory of a sample iC++ group animal during recording session with laser application (marked with a blue dashed square) in the north and east arms of the track. The experimenter actively guided the animal towards the east or north sections of the track (marked with red arrows) for 50% of the recording session. The access towards the SW corner was repeatedly restricted, which led to SW/NE ratio of 0.51. Right: weighted SPV of the spikes (colored dots) from the place cells (represented by different colors) recorded during same session with value of 42.2°. The forced navigation technique was chosen instead of compartmental obstruction for particular section of the maze. The compartmentalization of recording arena evokes remapping of place fields [1]. Files dataset is available at Figshare public repository in Tsanov 2017 data /Rectangular track forced navigation folder https://figshare.com/s/5c5ba9b2811f3d7b7696. 1. O'Keefe J, Burgess N. Geometric determinants of the place fields of hippocampal neurons. Nature. 1996;381(6581):425–8. 8632799. (TIF) [file pbio.2002365.s012.tif]

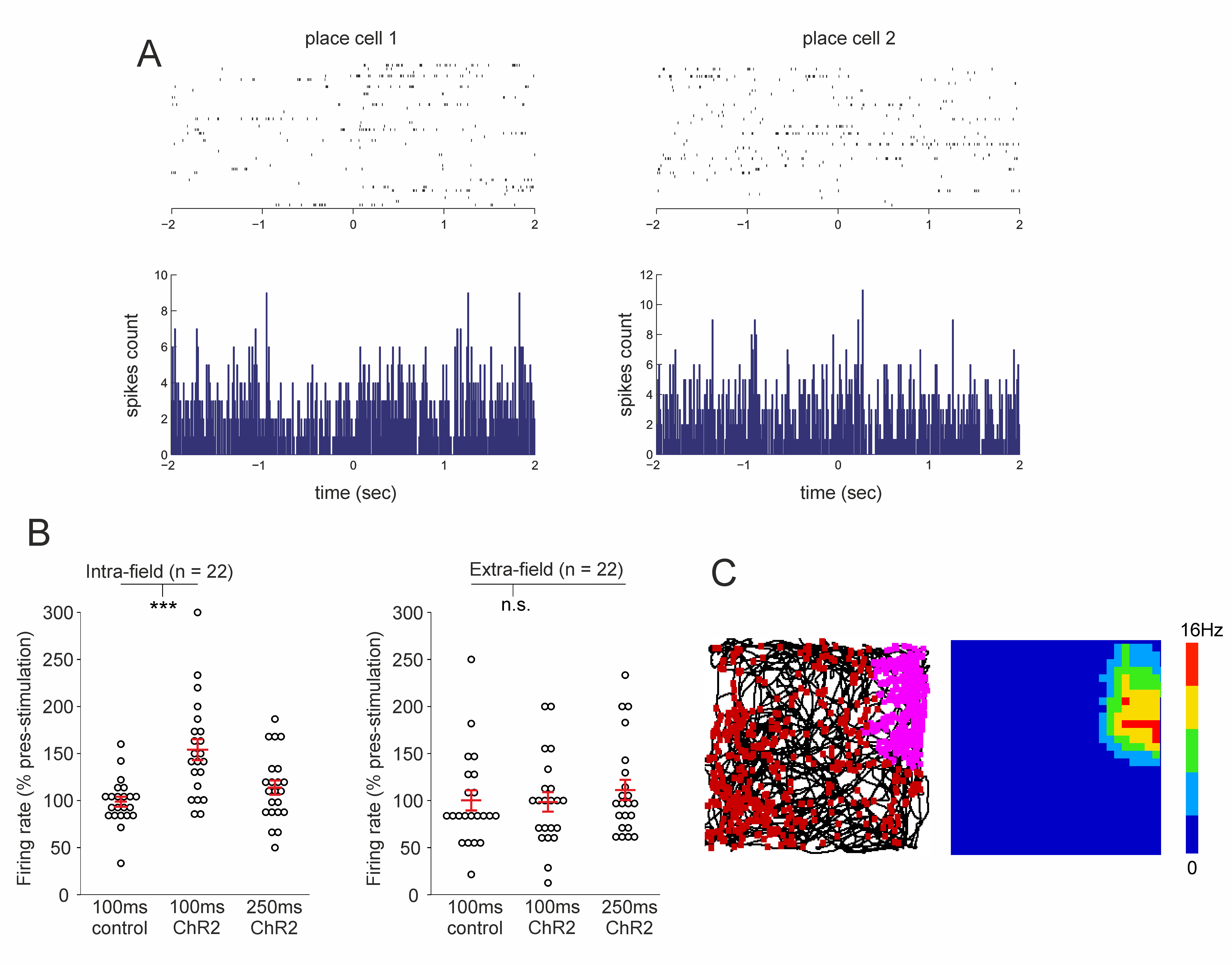

Supplement: S13 Fig — (A) Raster plot from 40 repetitions (above) and spike count of 120 repetitions (below) of two place cells. (B) Intra-field (left graph) and extra-field (right graph) firing rate of 22 place cells 100 ms after the onset of the stimulation protocol expressed as percentage of the pre-stimulation values, for control (left) and photostimulation (middle). The right dots show the firing rate (% pre-stimulation) for 250 ms after photostimulation onset. Paired t-test test: for intra-field control vs 100ms ChR2 t(21) = -4.344, ***P < 0.001. Error bars, mean ± s.e.m. Paired t-test test: for extra-field control vs 100ms ChR2 t(21) = 0.111, P = 0.913; for extra-field control vs 250ms ChR2 t(21) = -0.871, P = 0.394. Error bars, mean ± s.e.m. (C) Sample place field map of CA1 pyramidal neuron after exploration of open square arena. The map represents animal’s trajectory with spikes as coloured dots. The intra-field spikes are marked in purple, while the extra-field spikes are marked in red. The image on the right represents the corresponding firing rate map for the same place cell. Files dataset is available at Figshare public repository in Tsanov 2016 data / E123T electrophysiology folder https://figshare.com/s/b86a9a111353ba04bd32. (TIF) [file pbio.2002365.s013.tif]

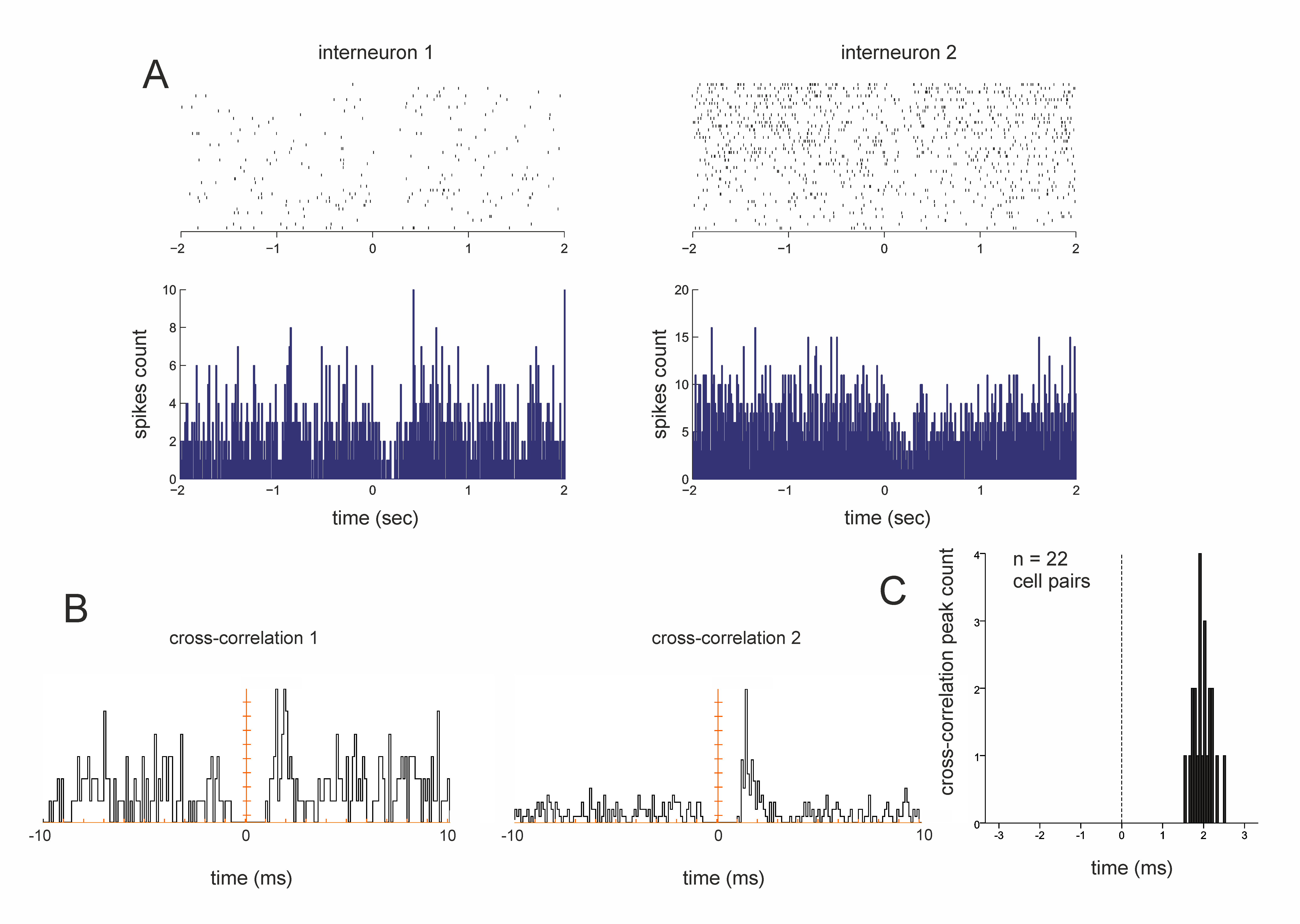

Supplement: S14 Fig — (A) Raster plot from 40 repetitions (above) and spike count of 120 repetitions (below) of two slow-spiking interneurons. (B) Spike train cross-correlogram between the interneurons and the place cells shown in (SA Fig). (C) Summary histogram of the spiking cross-correlation peak values for recorded place cell-interneuron pairs (n = 22). Files dataset is available at Figshare public repository in Tsanov 2016 data / Open arena regular stimulation folder https://figshare.com/s/b86a9a111353ba04bd32. (TIF) [file pbio.2002365.s014.tif]

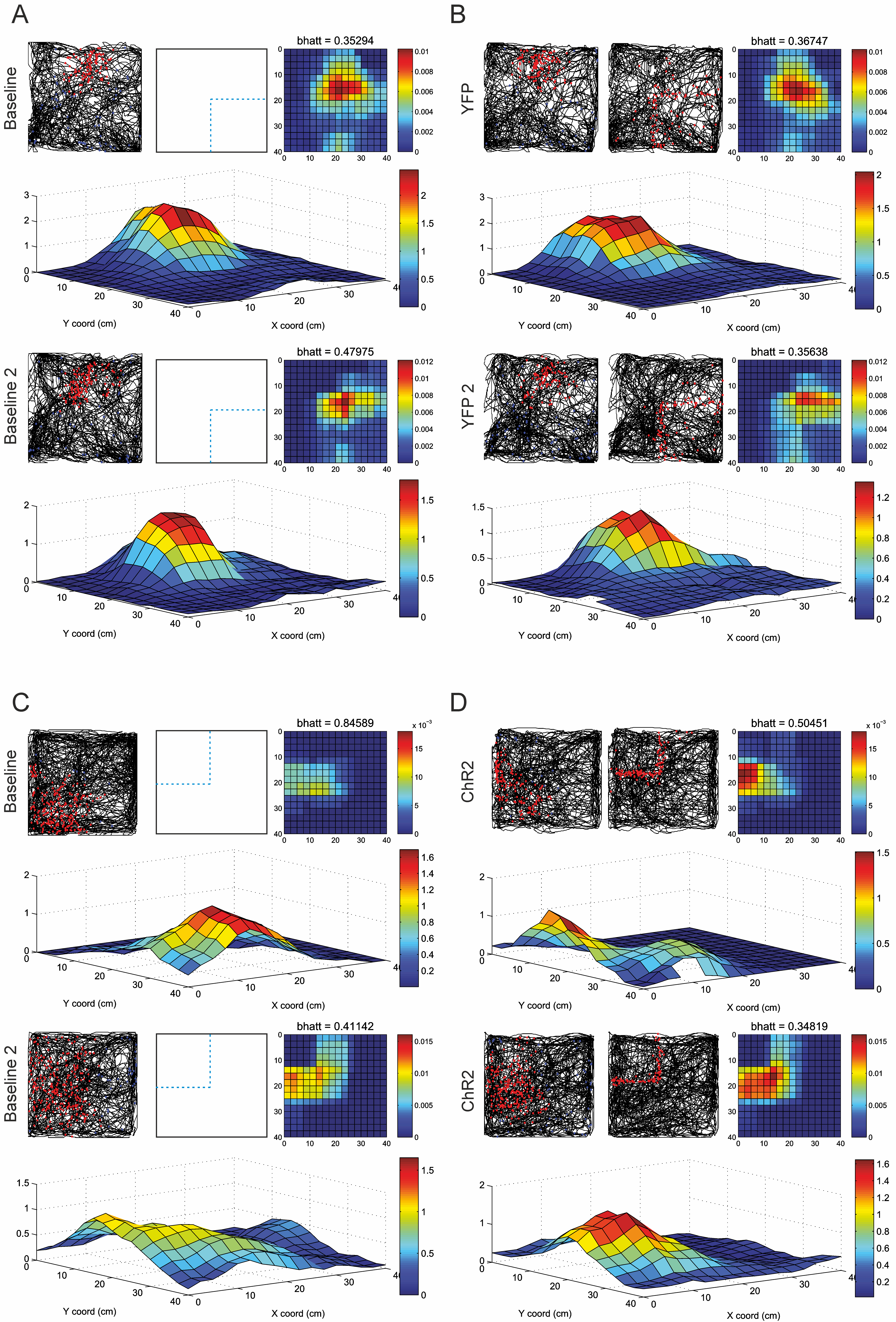

Supplement: S15 Fig — (A) Firing map of a sample place cell from the first (above) and the second (below) baseline recordings, and (B) firing map of the same sample place cell from the first (above) and second (below) control YFP light application. Top left panels represent the animal trajectory with spikes (red dots), top middle panels show the coordinates of the laser application (blue dashed lines) and the applied light pulses (red dots); and top right panels show color-coded bhatt overlap between the distribution of the spikes and the applied light pulses. Bottom images show 3D color-coded firing rate maps of the place cells. (C) Firing map of a sample place cell from the first (above) and the second (below) baseline recordings and (D) the spikes from the first (above) and second (below) ChR2 photostimulation session. Top left panels represent the animal trajectory with spikes (red dots), top middle panels show the coordinates of the laser application (blue dashed lines) and the applied light pulses (red dots); and top right panels show color-coded bhatt overlap between the distribution of the spikes and the applied light pulses. Bottom images show 3D color-coded firing rate maps of the place cells. Files dataset is available at Figshare public repository in Tsanov 2016 data / Open arena spatial stimulation folder https://figshare.com/s/b86a9a111353ba04bd32 and Tsanov 2016 data addition folder https://figshare.com/s/b2c6e7a8a0417820720c. (TIF) [file pbio.2002365.s015.tif]

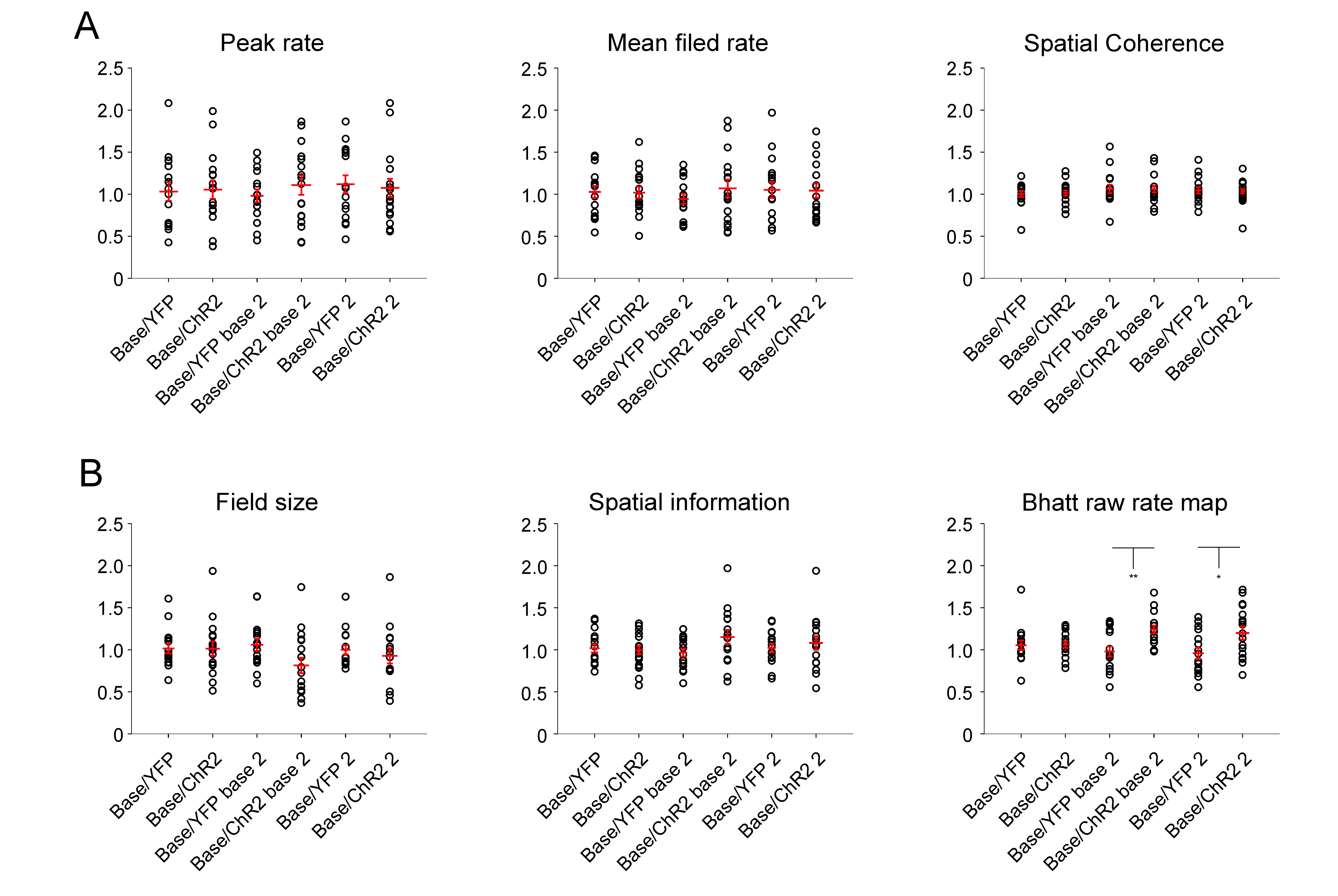

Supplement: S16 Fig — (A) Comparison of the place field center rate (left), mean filed rate (middle) and spatial coherence (right) between baselines and light delivery sessions of the control YFP group (n = 16 cells) and ChR2 group of rats (n = 18 cells). (B) Comparison of the field size (left), spatial information content (middle) and raw Bhattacharyya distance (right) between baselines and light delivery sessions of the control YFP group (n = 16 cells) and ChR2 group of rats (n = 18 cells). The place field size ratio of the baseline over the second baseline underwent non-significant decrease for the ChR2 group, two-tailed independent t-test test, t(31) = -1.886, p = 0.069. Concurrently, the spatial information ratio of the baseline over the second baseline underwent non-significant increase for the ChR2 group, t(31) = 1.895, p = 0.068. The raw Bhattacharyya distance (bhatt) was calculated for the non-smoothed rate maps for the ChR2 group (n = 18) and control YFP group (n = 16), two-tailed independent t-test test, baseline 2 session, t(31) = 3.547, **P = 0.001; light delivery 2 session, t(31) = 2.539, **P = 0.016. Error bars, mean ± s.e.m. The values are represented as ratios of the measured values from first baseline session over the values of the subsequent light delivery, second baseline and second light delivery session. Tsanov 2016 data / Open arena spatial stimulation folder https://figshare.com/s/b86a9a111353ba04bd32 and Tsanov 2016 data addition folder https://figshare.com/s/b2c6e7a8a0417820720c. (TIF) [file pbio.2002365.s016.tif]

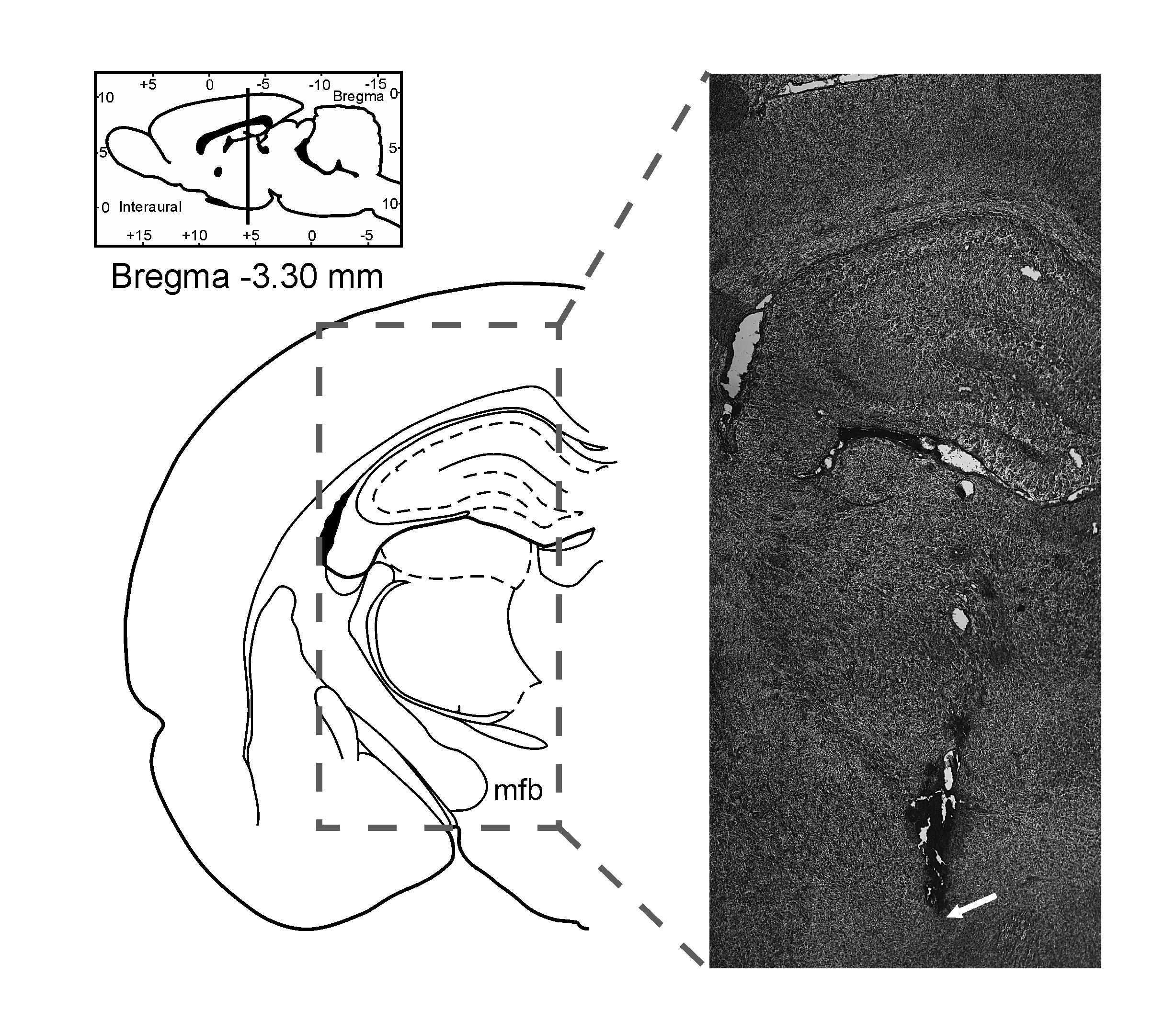

Supplement: S17 Fig — Inset atlas schematic shows the location of chronically-implanted electrodes in the medial forebrain bundle (mfb). A sample histology shows the tip of the bipolar concentric electrode (marked with white arrow). For verification of electrode location electrolytic lesion was induced by high-current intensity (0.5 mA current applied for a period of 10 s) after the completion of the experiments under non-recovery isoflurane anaesthesia. Files dataset is available at Figshare public repository in Tsanov 2016 data / Open arena spatial stimulation folder https://figshare.com/s/b86a9a111353ba04bd32. (TIF) [file pbio.2002365.s017.tif]

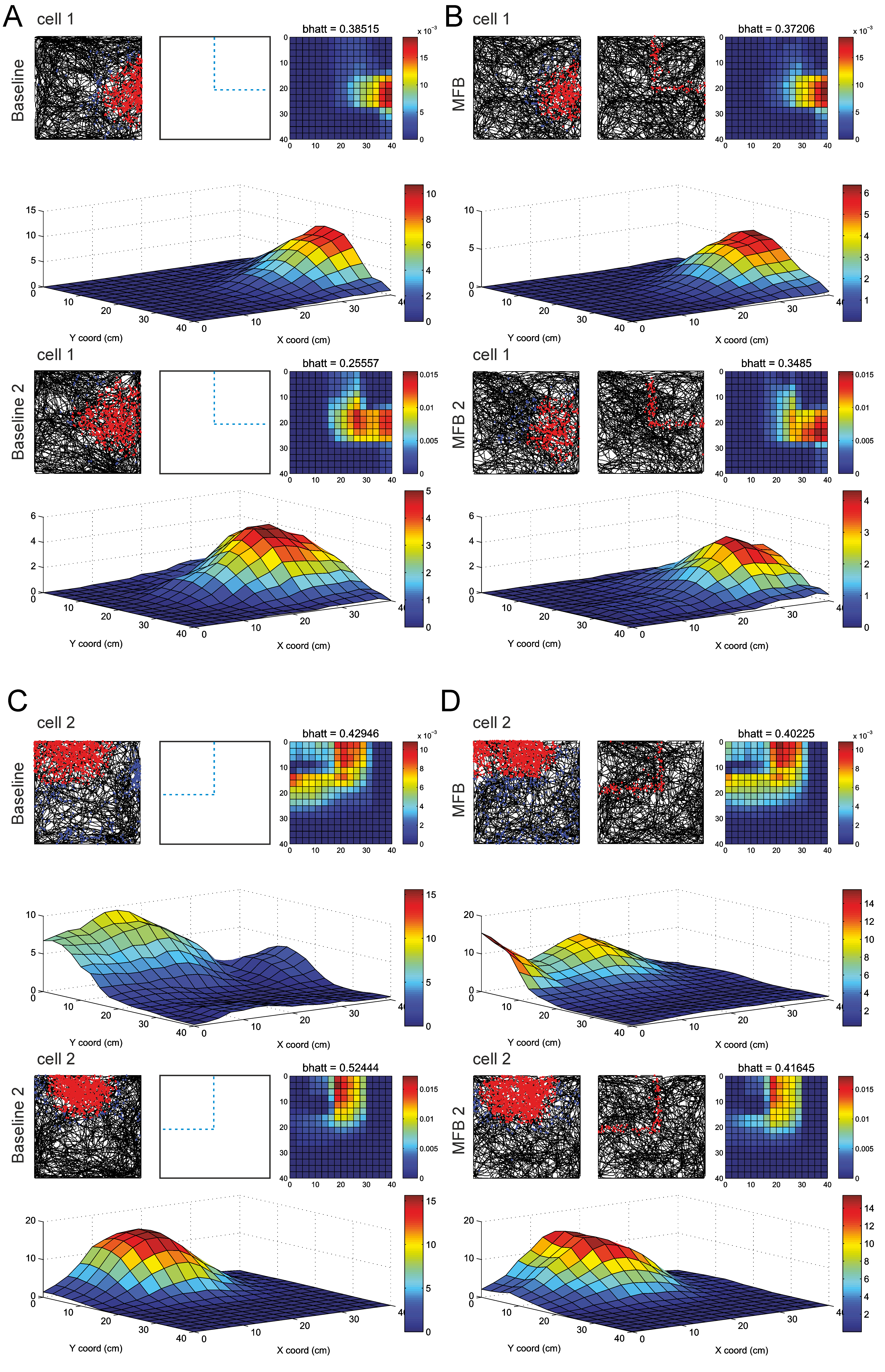

Supplement: S18 Fig — (TIF) [file pbio.2002365.s018.tif]

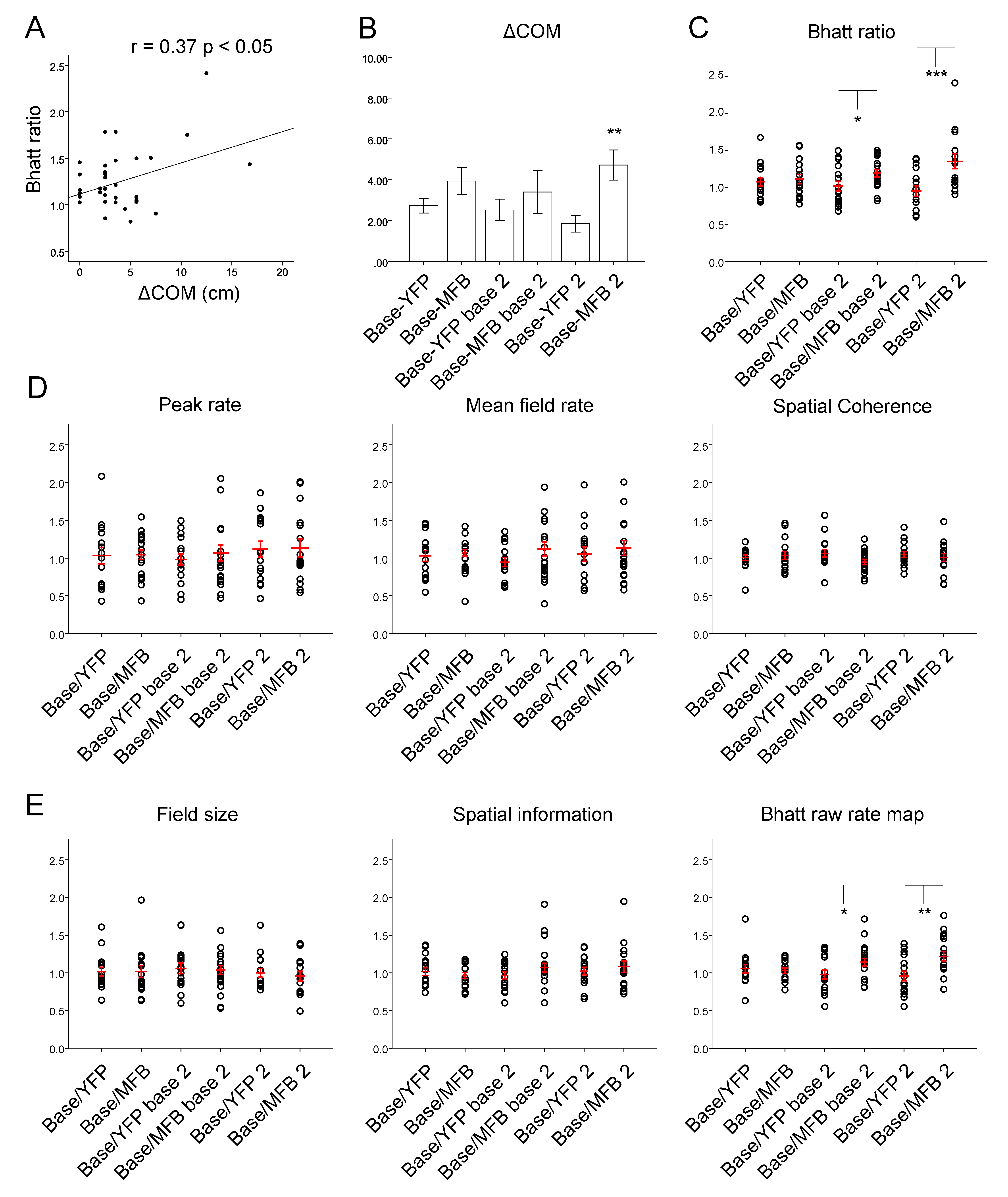

Supplement: S19 Fig — (A) Pearson’s correlation between bhatt and ΔCOM for the MFB group. (B) The difference of the center of mass (ΔCOM) between the baseline and the following first light delivery and MFB stimulation; baseline and second baseline; baseline and second light delivery and MFB stimulation session, two-tailed independent t-test test; t(32) = 3.470, **P = 0.002 for YFP control (n = 16 cells) and MFB stimulation (n = 18 cells) groups. Error bars, mean ± s.e.m. (C) Ratio of Bhattacharyya distance (bhatt) of the baseline values over the following first light delivery and MFB stimulation; baseline over second baseline, two-tailed independent t-test test, t(32) = 2.041, *P = 0.049; baseline over second light delivery and MFB stimulation session, t(32) = 3.040, **P = 0.005, for YFP control (n = 16) and MFB stimulation (n = 18) groups. Error bars, mean ± s.e.m. (D) Comparison of the place field peak rate (left), mean field rate (middle) and spatial coherence (right) between baselines and light delivery sessions of the control YFP and MFB group of rats. (E) Comparison of the place field size (left) and spatial information (middle) between baselines and light delivery sessions of the control YFP and MFB group of rats. Right: raw bhatt calculated for the non-smoothed rate map for the MFB group (n = 18) and control YFP group (n = 16), two-tailed independent t-test test, baseline 2 session, t(32) = 2.415, *P = 0.022; light delivery 2 and MFB 2 stimulation t(32) = 3.256, **P = 0.003. Error bars, mean ± s.e.m. Files dataset is available at Figshare public repository in Tsanov 2016 data / Open arena spatial stimulation folder https://figshare.com/s/b86a9a111353ba04bd32. (TIF) [file pbio.2002365.s019.tif]
