# Supplementary material for: Place field assembly distribution encodes preferred locations
Source: PLoS Biol. 2017 Sep 12;15(9):e2002365. doi: 10.1371/journal.pbio.2002365 (PMC5609775; doi:10.1371/journal.pbio.2002365)
Supplement: S1 Table — (DOCX) [file pbio.2002365.s020.docx]

**S1 Table**: number of passes in the probe session of continuous T-maze task.

| # rat | Central choice point,  towards South | Central choice point,  towards East | Starting choice point,  towards South | Starting choice point,  towards East | Both choice points,  towards South | Both choice points,  towards East | NE/SW Ratio | bi-normal p |
| --- | --- | --- | --- | --- | --- | --- | --- | --- |
| Rat 1 | 24 | 4 | 9 | 24 | 33 | 28 | 1.1785 | 0.0831 |
| Rat 2 | 2 | 12 | 3 | 21 | 5 | 33 | 0.1515 | 0.0001 |
| Rat 3 | 0 | 1 | 10 | 12 | 10 | 13 | 0.7692 | 0.1363 |
| Rat 4 | 9 | 28 | 8 | 16 | 17 | 44 | 0.3863 | 0.0002 |
| Rat 5 | 10 | 11 | 15 | 14 | 25 | 25 | 1 | 0.1122 |
| Rat 6 | 4 | 3 | 10 | 5 | 14 | 8 | 1.75 | 0.0762 |
| Rat 7 | 0 | 8 | 0 | 15 | 0 | 23 | 0 | 0.0001 |
| Rat 8 | 15 | 13 | 8 | 20 | 23 | 33 | 0.6969 | 0.0439 |
| Rat 9 | 7 | 24 | 12 | 27 | 19 | 51 | 0.3725 | 0.0001 |
| Rat 10 | 6 | 16 | 12 | 16 | 18 | 32 | 0.5625 | 0.0160 |
| Rat 11 | 13 | 29 | 8 | 17 | 21 | 46 | 0.4565 | 0.0008 |
| Rat 12 | 8 | 1 | 12 | 15 | 20 | 16 | 1.25 | 0.1063 |
| Rat 13 | 3 | 8 | 15 | 11 | 18 | 19 | 0.9473 | 0.1285 |
| Rat 14 | 7 | 14 | 18 | 11 | 25 | 25 | 1 | 0.11228 |
| Rat 15 | 20 | 3 | 6 | 20 | 26 | 23 | 1.1304 | 0.10364 |
| Rat 16 | 12 | 27 | 12 | 29 | 24 | 56 | 0.4285 | 0.00013 |
| Rat 17 | 4 | 9 | 16 | 12 | 20 | 21 | 0.9523 | 0.12239 |
| Rat 18 | 10 | 16 | 11 | 31 | 21 | 47 | 0.4468 | 0.00064 |
| Rat 19 | 6 | 5 | 13 | 8 | 19 | 13 | 1.4615 | 0.08088 |
| Rat 20 | 1 | 24 | 7 | 20 | 8 | 44 | 0.1818 | 0.00000 |
