# Supplementary material for: Place field assembly distribution encodes preferred locations
Source: PLoS Biol. 2017 Sep 12;15(9):e2002365. doi: 10.1371/journal.pbio.2002365 (PMC5609775; doi:10.1371/journal.pbio.2002365)
Supplement: S2 Table — (DOCX) [file pbio.2002365.s021.docx]

**S2 Table**: Spatial filed configuration (degrees) for all cells in the probe session of T-maze task.

| cell # | 1 | | 2 | | 3 | | | 4 | | 5 | | 6 | | | 7 | | 8 | | 9 | |
| --- | --- | --- | --- | --- | --- | --- | --- | --- | --- | --- | --- | --- | --- | --- | --- | --- | --- | --- | --- | --- |
| Rat 1 | 61.9 | | 64.8 | | 42.9 | | | 0.0 | | 24.0 | | 56.1 | | | 57.3 | | 12.4 | | 81.4 | |
| Rat 2 | 82.1 | | 69.1 | | 53.4 | | | 90.0 | | 68.5 | | 90.0 | | | 90.0 | | 90.0 | | 18.0 | |
| Rat 3 | 54.0 | | 33.8 | | 55.3 | | | 4.5 | | 30.3 | | 59.1 | | | 67.1 | | 53.5 | | 42.2 | |
| Rat 4 | 74.4 | | 32.9 | | 2.3 | | | 63.6 | | 75.7 | |  | | |  | |  | |  | |
| Rat 5 | 68.1 | | 37.3 | | 0.0 | | | 0.0 | | 58.9 | | 52.9 | | | 21.6 | | 21.7 | | 67.1 | |
| Rat 6 | 90.0 | | 28.5 | | 0.0 | | | 30.4 | |  | |  | | |  | |  | |  | |
| Rat 7 | 85.0 | | 49.5 | | 53.5 | | | 90.0 | | 70.7 | | 43.0 | | | 59.7 | | 63.9 | | 90.0 | |
| Rat 8 | 55.8 | | 49.9 | | 90.0 | | | 0.0 | | 54.8 | | 90.0 | | | 59.6 | | 36.7 | | 15.7 | |
| Rat 9 | 77.4 | | 36.0 | | 32.9 | | | 25.4 | | 0.3 | | 90.0 | | | 81.6 | | 82.8 | | 90.0 | |
| Rat 10 | 16.1 | | 19.5 | | 90.0 | | | 90.0 | | 90.0 | | 90.0 | | | 8.3 | | 90.0 | | 11.5 | |
| Rat 11 | 36.9 | | 60.9 | | 89.8 | | | 90.0 | | 9.7 | | 62.8 | | | 90.0 | | 38.8 | | 81.9 | |
| Rat 12 | 0.0 | | 38.3 | | 0.0 | | | 0.0 | | 57.0 | | 38.6 | | | 47.0 | | 90.0 | | 0.1 | |
| Rat 13 | 22.1 | | 20.5 | | 28.4 | | | 72.2 | | 27.6 | | 25.3 | | | 13.9 | | 83.0 | | 0.0 | |
| cell # | 10 | 11 | | 12 | | 13 | 14 | | 15 | | 16 | | 17 | 18 | | 19 | | 20 | | 21 |
| Rat 1 |  |  | |  | |  |  | |  | |  | |  |  | |  | |  | |  |
| Rat 2 | 3.8 | 37.6 | | 0.0 | | 0.0 | 6.0 | |  | |  | |  |  | |  | |  | |  |
| Rat 3 |  |  | |  | |  |  | |  | |  | |  |  | |  | |  | |  |
| Rat 4 |  |  | |  | |  |  | |  | |  | |  |  | |  | |  | |  |
| Rat 5 | 90.0 | 63.0 | | 27.7 | | 15.8 | 76.3 | | 34.7 | | 10.1 | |  |  | |  | |  | |  |
| Rat 6 |  |  | |  | |  |  | |  | |  | |  |  | |  | |  | |  |
| Rat 7 | 0.0 | 29.5 | |  | |  |  | |  | |  | |  |  | |  | |  | |  |
| Rat 8 |  |  | |  | |  |  | |  | |  | |  |  | |  | |  | |  |
| Rat 9 | 68.9 | 90.0 | | 29.6 | | 57.8 | 90.0 | | 23.1 | | 1.3 | | 0.0 | 0.0 | | 34.5 | | 90.0 | | 4.2 |
| Rat 10 | 20.4 | 0.0 | | 31.6 | | 0.0 | 21.8 | | 47.4 | | 33.2 | |  |  | |  | |  | |  |
| Rat 11 | 24.1 | 76.3 | | 0.0 | | 69.5 | 59.0 | |  | |  | |  |  | |  | |  | |  |
| Rat 12 | 0.0 | 2.5 | | 77.7 | | 90.0 | 27.1 | | 0.0 | | 24.9 | | 90.0 | 17.2 | | 18.3 | | 89.0 | | 0.0 |
| Rat 13 | 31.6 | 81.4 | | 90.0 | | 0.0 | 66.1 | | 16.6 | | 90.0 | | 90.0 | 25.0 | | 77.2 | |  | |  |

| cell # | 1 | | 2 | | 3 | | | 4 | | 5 | | 6 | | | 7 | | 8 | | 9 | |
| --- | --- | --- | --- | --- | --- | --- | --- | --- | --- | --- | --- | --- | --- | --- | --- | --- | --- | --- | --- | --- |
| Rat 14 | 0.0 | | 2.2 | | 12.0 | | | 89.8 | | 55.2 | | 58.1 | | | 19.3 | | 25.6 | | 22.5 | |
| Rat 15 | 0.0 | | 57.4 | | 71.9 | | | 23.7 | | 0.0 | | 37.0 | | | 4.0 | | 0.0 | | 67.6 | |
| Rat 16 | 16.0 | | 23.2 | | 0.0 | | | 90.0 | | 87.0 | | 55.6 | | | 89.3 | | 64.8 | | 0.0 | |
| Rat 17 | 85.5 | | 60.1 | | 14.4 | | | 0.0 | | 37.4 | |  | | |  | |  | |  | |
| Rat 18 | 90.0 | | 90.0 | | 74.0 | | | 0.0 | | 0.0 | | 90.0 | | | 0.0 | | 64.6 | | 90.0 | |
| Rat 19 | 0.0 | | 4.9 | | 0.0 | | | 64.5 | | 90.0 | | 90.0 | | | 0.0 | | 0.0 | | 15.0 | |
| Rat 20 | 90.0 | | 57.1 | | 26.4 | | | 37.5 | | 89.5 | |  | | |  | |  | |  | |
| cell # | 10 | 11 | | 12 | | 13 | 14 | | 15 | | 16 | | 17 | 18 | | 19 | | 20 | | 21 |
| Rat 14 | 33.8 | 28.3 | |  | |  |  | |  | | 33.8 | | 28.3 |  | |  | |  | |  |
| Rat 15 | 90.0 | 36.9 | | 88.8 | |  |  | |  | | 90.0 | | 36.9 | 88.8 | |  | |  | |  |
| Rat 16 | 90.0 | 90.0 | | 74.4 | | 55.7 |  | |  | | 90.0 | | 90.0 | 74.4 | | 55.7 | |  | |  |
| Rat 17 |  |  | |  | |  |  | |  | |  | |  |  | |  | |  | | 90.0 |
| Rat 18 | 63.7 | 90.0 | | 0.0 | | 90.0 | 55.0 | | 90.0 | | 63.7 | | 90.0 | 0.0 | | 90.0 | | 55.0 | |  |
| Rat 19 | 0.2 | 90.0 | | 78.6 | | 15.5 |  | |  | | 0.2 | | 90.0 | 78.6 | | 15.5 | |  | |  |
| Rat 20 |  |  | |  | |  |  | |  | |  | |  |  | |  | |  | |  |
