# Supplementary material for: Place field assembly distribution encodes preferred locations
Source: PLoS Biol. 2017 Sep 12;15(9):e2002365. doi: 10.1371/journal.pbio.2002365 (PMC5609775; doi:10.1371/journal.pbio.2002365)
Supplement: S3 Table — (DOCX) [file pbio.2002365.s022.docx]

**S3 Table**: Center of mass angle (degrees) for all cells in the probe session of T-maze task.

| cell # | 1 | | 2 | | 3 | | | 4 | | 5 | | 6 | | | 7 | | 8 | | 9 | |
| --- | --- | --- | --- | --- | --- | --- | --- | --- | --- | --- | --- | --- | --- | --- | --- | --- | --- | --- | --- | --- |
| Rat 1 | 46.3 | | 49.0 | | 45.6 | | | 4.5 | | 42.8 | | 49.2 | | | 51.1 | | 40.5 | | 46.3 | |
| Rat 2 | 63.3 | | 48.0 | | 46.7 | | | 65.7 | | 51.0 | | 87.3 | | | 81.3 | | 83.2 | | 63.3 | |
| Rat 3 | 48.0 | | 45.3 | | 56.6 | | | 31.5 | | 45.3 | | 55.9 | | | 50.6 | | 46.1 | | 48.0 | |
| Rat 4 | 58.9 | | 43.1 | | 18.4 | | | 54.3 | | 59.1 | |  | | |  | |  | | 58.9 | |
| Rat 5 | 51.3 | | 41.9 | | 20.2 | | | 19.8 | | 48.2 | | 49.2 | | | 38.6 | | 36.4 | | 51.3 | |
| Rat 6 | 66.6 | | 42.5 | | 14.6 | | | 46.0 | |  | |  | | |  | |  | | 66.6 | |
| Rat 7 | 66.1 | | 46.5 | | 45.5 | | | 70.2 | | 53.5 | | 42.3 | | | 49.4 | | 45.6 | | 66.1 | |
| Rat 8 | 49.6 | | 49.4 | | 84.4 | | | 4.6 | | 45.6 | | 69.4 | | | 49.4 | | 43.1 | | 49.6 | |
| Rat 9 | 62.0 | | 40.0 | | 41.6 | | | 42.9 | | 31.2 | | 92.0 | | | 55.7 | | 70.5 | | 62.0 | |
| Rat 10 | 41.8 | | 33.1 | | 80.0 | | | 68.3 | | 119.8 | | 72.5 | | | 27.1 | | 62.9 | | 41.8 | |
| Rat 11 | 43.8 | | 49.5 | | 72.3 | | | 84.4 | | 27.8 | | 56.7 | | | 52.5 | | 38.9 | | 43.8 | |
| Rat 12 | 16.2 | | 40.3 | | 5.9 | | | 33.9 | | 50.7 | | 45.1 | | | 46.2 | | 75.1 | | 16.2 | |
| Rat 13 | 42.3 | | 43.9 | | 45.7 | | | 49.0 | | 40.1 | | 38.8 | | | 42.4 | | 59.6 | | 42.3 | |
| cell # | 10 | 11 | | 12 | | 13 | 14 | | 15 | | 16 | | 17 | 18 | | 19 | | 20 | | 21 |
| Rat 1 |  |  | |  | |  |  | |  | |  | |  |  | |  | |  | |  |
| Rat 2 | 20.2 | 42.7 | | 15.8 | | 7.5 | 30.5 | |  | |  | |  |  | |  | |  | |  |
| Rat 3 |  |  | |  | |  |  | |  | |  | |  |  | |  | |  | |  |
| Rat 4 |  |  | |  | |  |  | |  | |  | |  |  | |  | |  | |  |
| Rat 5 | 85.4 | 45.9 | | 36.4 | | 32.9 | 56.6 | | 41.2 | | 22.6 | |  |  | |  | |  | |  |
| Rat 6 |  |  | |  | |  |  | |  | |  | |  |  | |  | |  | |  |
| Rat 7 | 12.8 | 44.0 | |  | |  |  | |  | |  | |  |  | |  | |  | |  |
| Rat 8 |  |  | |  | |  |  | |  | |  | |  |  | |  | |  | |  |
| Rat 9 | 58.3 | 91.9 | | 41.5 | | 50.8 | 76.0 | | 35.6 | | 26.6 | | 15.2 | 8.6 | | 39.9 | | 86.7 | | 25.8 |
| Rat 10 | 33.0 | 17.7 | | 46.2 | | 16.6 | 48.9 | | 47.5 | | 41.4 | |  |  | |  | |  | |  |
| Rat 11 | 43.7 | 62.7 | | 18.6 | | 57.9 | 46.5 | |  | |  | |  |  | |  | |  | |  |
| Rat 12 | 24.9 | 13.2 | | 58.4 | | 61.6 | 37.5 | | 8.8 | | 34.6 | | 83.1 | 37.6 | | 23.9 | | 70.3 | | 20.4 |
| Rat 13 | 36.5 | 57.2 | | 47.8 | | 31.2 | 53.1 | | 44.8 | | 64.0 | | 85.9 | 35.0 | | 60.2 | |  | |  |
|  |  |  | |  | |  |  | |  | |  | |  |  | |  | |  | |  |

| cell # | 1 | | 2 | | 3 | | | 4 | | 5 | | 6 | | | 7 | | 8 | | 9 | |
| --- | --- | --- | --- | --- | --- | --- | --- | --- | --- | --- | --- | --- | --- | --- | --- | --- | --- | --- | --- | --- |
| Rat 14 | 22.2 | | 27.1 | | 29.9 | | | 65.6 | | 45.8 | | 45.8 | | | 23.2 | | 40.8 | | 22.2 | |
| Rat 15 | 9.1 | | 48.0 | | 58.0 | | | 35.1 | | 2.2 | | 43.6 | | | 30.9 | | 11.1 | | 9.1 | |
| Rat 16 | 44.3 | | 34.3 | | 24.5 | | | 114.7 | | 63.2 | | 46.9 | | | 59.0 | | 47.5 | | 44.3 | |
| Rat 17 | 57.4 | | 45.9 | | 32.5 | | | 16.2 | | 44.5 | |  | | |  | |  | | 57.4 | |
| Rat 18 | 62.3 | | 83.7 | | 55.1 | | | 25.6 | | 48.4 | | 72.7 | | | 6.3 | | 55.4 | | 62.3 | |
| Rat 19 | 13.9 | | 27.5 | | 15.3 | | | 48.9 | | 73.6 | | 68.9 | | | 33.8 | | 14.3 | | 13.9 | |
| Rat 20 | 91.6 | | 46.3 | | 39.5 | | | 45.1 | | 73.4 | |  | | |  | |  | | 91.6 | |
| cell # | 10 | 11 | | 12 | | 13 | 14 | | 15 | | 16 | | 17 | 18 | | 19 | | 20 | | 21 |
| Rat 14 | 40.6 | 45.6 | |  | |  |  | |  | | 40.6 | | 45.6 |  | |  | |  | |  |
| Rat 15 | 69.6 | 48.0 | | 66.7 | |  |  | |  | | 69.6 | | 48.0 | 66.7 | |  | |  | |  |
| Rat 16 | 89.5 | 89.1 | | 49.8 | | 47.0 |  | |  | | 89.5 | | 82.1 | 49.8 | | 47.0 | |  | |  |
| Rat 17 |  |  | |  | |  |  | |  | |  | |  |  | |  | |  | |  |
| Rat 18 | 49.5 | 65.4 | | 17.4 | | 70.5 | 59.8 | | 85.5 | | 49.5 | | 65.4 | 17.4 | | 70.5 | | 59.8 | | 85.5 |
| Rat 19 | 31.8 | 82.6 | | 56.0 | | 34.1 |  | |  | | 31.8 | | 82.6 | 56.0 | | 34.1 | |  | |  |
| Rat 20 |  |  | |  | |  |  | |  | |  | |  |  | |  | |  | |  |
