# Supplementary material for: Place field assembly distribution encodes preferred locations
Source: PLoS Biol. 2017 Sep 12;15(9):e2002365. doi: 10.1371/journal.pbio.2002365 (PMC5609775; doi:10.1371/journal.pbio.2002365)
Supplement: S4 Table — (DOCX) [file pbio.2002365.s023.docx]

**S4 Table**: Spatial population vector (degrees) in the probe session of continuous T-maze task.

| Name | # reward loop | weighted SPV reward loop cells | average SPV reward loop cells | # all cells | weighted SPV all cells | averaged SPV all cells |
| --- | --- | --- | --- | --- | --- | --- |
| Rat 1 | 7 | 45.92 | 41.223 | 9 | 46.025 | 43.975 |
| Rat 2 | 11 | 56.819 | 56.132 | 14 | 50.948 | 48.262 |
| Rat 3 | 8 | 48.037 | 47.415 | 9 | 48.031 | 47.4 |
| Rat 4 | 4 | 54.481 | 53.87 | 5 | 50.359 | 46.774 |
| Rat 5 | 9 | 38.69 | 37.132 | 16 | 46.499 | 42.72 |
| Rat 6 | 3 | 30.336 | 34.374 | 4 | 32.305 | 42.422 |
| Rat 7 | 9 | 58.326 | 57.265 | 11 | 56.674 | 51.861 |
| Rat 8 | 7 | 47.67 | 48.114 | 9 | 46.754 | 46.949 |
| Rat 9 | 12 | 57.106 | 63.666 | 21 | 51.33 | 51.203 |
| Rat 10 | 10 | 49.564 | 52.64 | 16 | 46.188 | 48.961 |
| Rat 11 | 10 | 58.709 | 52.069 | 14 | 54.411 | 47.701 |
| Rat 12 | 16 | 29.874 | 32.543 | 21 | 40.312 | 37.116 |
| Rat 13 | 15 | 45.743 | 47.187 | 19 | 46.29 | 46.692 |
| Rat 14 | 9 | 39.54 | 38.342 | 11 | 39.666 | 39.203 |
| Rat 15 | 9 | 40.491 | 38.314 | 12 | 41.757 | 39.216 |
| Rat 16 | 11 | 55.161 | 58.761 | 13 | 54.67 | 57.166 |
| Rat 17 | 3 | 47.345 | 45.266 | 5 | 44.431 | 39.304 |
| Rat 18 | 12 | 58.974 | 56.965 | 15 | 56.359 | 55.676 |
| Rat 19 | 10 | 37.946 | 36.623 | 13 | 41.76 | 42.816 |
| Rat 20 | 4 | 64.42 | 62.714 | 5 | 56.931 | 59.189 |
