# Supplementary material for: Place field assembly distribution encodes preferred locations
Source: PLoS Biol. 2017 Sep 12;15(9):e2002365. doi: 10.1371/journal.pbio.2002365 (PMC5609775; doi:10.1371/journal.pbio.2002365)
Supplement: S5 Table — (DOCX) [file pbio.2002365.s024.docx]

**S5 Table**: Number of passes and SPV in rectangular-shaped linear task for iC++ rats.

|  | passes SW | passes NE | SW/NE ratio | weighted SPV | averaged SPV |
| --- | --- | --- | --- | --- | --- |
| Rat 1 baseline | 32 | 24 | 1.333 | 41.857 | 42.198 |
| Rat 1 iC++ session 1 | 39 | 55 | 0.709 | 49.099 | 53.573 |
| Rat 1 iC++ session 2 | 28 | 48 | 0.583 | 48.49 | 43.135 |
|  |  |  |  |  |  |
| Rat 2 baseline | 36 | 39 | 0.923 | 42.337 | 44.539 |
| Rat 2 iC++ session 1 | 26 | 32 | 0.812 | 45.675 | 46.615 |
| Rat 2 iC++ session 2 | 23 | 46 | 0.5 | 58.28 | 49.906 |
|  |  |  |  |  |  |
| Rat 3 baseline | 20 | 25 | 0.8 | 43.071 | 42.797 |
| Rat 3 iC++ session 1 | 15 | 23 | 0.652 | 45.636 | 46.764 |
| Rat 3 iC++ session 2 | 20 | 23 | 0.869 | 59.888 | 59.106 |
|  |  |  |  |  |  |
| Rat 4 baseline | 18 | 17 | 1.058 | 50.595 | 51.053 |
| Rat 4 iC++ session 1 | 31 | 32 | 0.968 | 67.103 | 63.318 |
| Rat 4 iC++ session 2 | 31 | 34 | 0.911 | 76.875 | 71.369 |
|  |  |  |  |  |  |
| Rat 5 baseline | 70 | 56 | 1.25 | 44.757 | 45.221 |
| Rat 5 iC++ session 1 | 23 | 34 | 0.676 | 42.027 | 45.609 |
| Rat 5 iC++ session 2 | 36 | 35 | 1.028 | 48.606 | 42.919 |
|  |  |  |  |  |  |
| Rat 6 baseline | 84 | 107 | 0.785 | 43.194 | 42.113 |
| Rat 6 iC++ session 1 | 74 | 109 | 0.678 | 50.58 | 47.235 |
| Rat 6 iC++ session 2 | 58 | 118 | 0.491 | 51.147 | 48.225 |
