# Supplementary material for: Place field assembly distribution encodes preferred locations
Source: PLoS Biol. 2017 Sep 12;15(9):e2002365. doi: 10.1371/journal.pbio.2002365 (PMC5609775; doi:10.1371/journal.pbio.2002365)
Supplement: S6 Table — (DOCX) [file pbio.2002365.s025.docx]

**S6 Table**: Number of passes and SPV in rectangular-shaped linear task for YFP rats.

| passes SW | passes SW | passes NE | SW/NE ratio | weighted SPV | averaged SPV |
| --- | --- | --- | --- | --- | --- |
| Rat 1 baseline | 52 | 44 | 1.182 | 55.825 | 56.138 |
| Rat 1 YFP session 1 | 51 | 40 | 1.275 | 58.907 | 57.947 |
| Rat 1 YFP session 2 | 46 | 43 | 1.070 | 58.578 | 58.624 |
|  |  |  |  |  |  |
| Rat 2 baseline | 63 | 72 | 0.875 | 44.7 | 39.928 |
| Rat 2 YFP session 1 | 68 | 63 | 1.079 | 43.533 | 39.485 |
| Rat 2 YFP session 2 | 71 | 68 | 1.044 | 45.205 | 39.746 |
|  |  |  |  |  |  |
| Rat 3 baseline | 73 | 91 | 0.802 | 38.703 | 51.142 |
| Rat 3 YFP session 1 | 67 | 113 | 0.593 | 31.963 | 42.095 |
| Rat 3 YFP session 2 | 58 | 78 | 0.744 | 28.773 | 43.818 |
|  |  |  |  |  |  |
| Rat 4 baseline | 59 | 69 | 0.855 | 42.461 | 43.557 |
| Rat 4 YFP session 1 | 50 | 59 | 0.847 | 41.646 | 45.209 |
| Rat 4 YFP session 2 | 43 | 74 | 0.581 | 43.504 | 47.019 |
|  |  |  |  |  |  |
| Rat 5 baseline | 64 | 64 | 1.000 | 47.306 | 50.232 |
| Rat 5 YFP session 1 | 50 | 50 | 1.000 | 51.678 | 50.898 |
| Rat 5 YFP session 2 | 62 | 49 | 1.265 | 44.439 | 47.736 |
|  |  |  |  |  |  |
| Rat 6 baseline | 45 | 46 | 1.013 | 46.589 | 44.173 |
| Rat 6 YFP session 1 | 57 | 43 | 1.326 | 43.945 | 39.788 |
| Rat 6 YFP session 2 | 53 | 60 | 0.883 | 46.031 | 41.998 |
|  |  |  |  |  |  |
| Rat 7 baseline | 53 | 37 | 1.432 | 42.739 | 39.478 |
| Rat 7 YFP session 1 | 49 | 52 | 0.942 | 53.331 | 42.782 |
| Rat 7 YFP session 2 | 58 | 45 | 1.289 | 56.227 | 47.698 |
