# Supplementary material for: Place field assembly distribution encodes preferred locations
Source: PLoS Biol. 2017 Sep 12;15(9):e2002365. doi: 10.1371/journal.pbio.2002365 (PMC5609775; doi:10.1371/journal.pbio.2002365)
Supplement: S7 Table — (DOCX) [file pbio.2002365.s026.docx]

**S7 Table**: post-photostimulation firing rate (% pre-stimulation).

| place cells | baseline | 100 ms | 250 ms | slow-spiking interneurons | baseline | 100 ms | 250 ms |
| --- | --- | --- | --- | --- | --- | --- | --- |
| cell 1 | 83.8 | 113.3 | 82.5 | cell 1 | 100.8 | 83.0 | 76.3 |
| cell 2 | 91.5 | 89.5 | 104.9 | cell 2 | 101.0 | 126.7 | 79.6 |
| cell 3 | 91.3 | 166.7 | 78.3 | cell 3 | 101.3 | 63.6 | 57.7 |
| cell 4 | 92.5 | 104.8 | 132.1 | cell 4 | 96.6 | 103.8 | 83.8 |
| cell 5 | 106.0 | 132.0 | 148.3 | cell 5 | 104.5 | 91.8 | 82.1 |
| cell 6 | 92.9 | 142.1 | 108.9 | cell 6 | 96.3 | 57.1 | 81.5 |
| cell 7 | 83.2 | 114.3 | 107.4 | cell 7 | 87.0 | 90.0 | 95.7 |
| cell 8 | 104.0 | 110.0 | 80.0 | cell 8 | 100.2 | 80.0 | 68.6 |
| cell 9 | 88.7 | 88.9 | 94.3 | cell 9 | 93.2 | 109.7 | 84.5 |
| cell 10 | 113.4 | 190.9 | 117.1 | cell 10 | 96.0 | 69.4 | 84.0 |
| cell 11 | 103.2 | 102.8 | 72.7 | cell 11 | 93.5 | 61.0 | 55.1 |
| cell 12 | 92.9 | 133.3 | 200.0 | cell 12 | 99.4 | 119.2 | 57.1 |
| cell 13 | 109.6 | 170.0 | 150.6 | cell 13 | 108.0 | 72.4 | 63.8 |
| cell 14 | 138.0 | 141.7 | 132.0 | cell 14 | 103.4 | 83.3 | 64.9 |
| cell 15 | 90.3 | 150.0 | 77.8 | cell 15 | 108.0 | 80.0 | 89.9 |
| cell 16 | 115.4 | 150.0 | 130.8 | cell 16 | 103.8 | 100.0 | 84.6 |
| cell 17 | 98.9 | 126.3 | 147.7 | cell 17 | 99.6 | 80.4 | 88.6 |
| cell 18 | 110.0 | 78.3 | 98.0 | cell 18 | 93.5 | 54.5 | 73.9 |
| cell 19 | 90.3 | 105.6 | 92.2 | cell 19 | 105.9 | 107.7 | 91.2 |
| cell 20 | 121.1 | 113.3 | 82.8 | cell 20 | 90.5 | 83.3 | 87.1 |
| cell 21 | 96.3 | 109.1 | 114.6 | cell 21 | 108.1 | 100.0 | 96.8 |
| cell 22 | 91.7 | 91.3 | 116.7 |  |  |  |  |
