# Supplementary material for: Place field assembly distribution encodes preferred locations
Source: PLoS Biol. 2017 Sep 12;15(9):e2002365. doi: 10.1371/journal.pbio.2002365 (PMC5609775; doi:10.1371/journal.pbio.2002365)
Supplement: S8 Table — (DOCX) [file pbio.2002365.s027.docx]

**S8 Table**: post-stimulation firing rate (% pre-stimulation) for intra- vs extra place field spikes.

| Intra-field | baseline | 100 ms | 250 ms | Extra-field | baseline | 100 ms | 250 ms |
| --- | --- | --- | --- | --- | --- | --- | --- |
| cell 1 | 83.3 | 115.4 | 90.0 | cell 1 | 90.0 | 100.0 | 60.0 |
| cell 2 | 101.6 | 145.5 | 119.4 | cell 2 | 60.0 | 12.5 | 60.0 |
| cell 3 | 86.8 | 150.0 | 63.2 | cell 3 | 90.0 | 200.0 | 100.0 |
| cell 4 | 92.5 | 104.8 | 132.1 | cell 4 | 50.0 | 100.0 | 100.0 |
| cell 5 | 121.0 | 176.9 | 164.5 | cell 5 | 88.9 | 57.1 | 129.6 |
| cell 6 | 100.0 | 170.0 | 110.5 | cell 6 | 77.8 | 111.1 | 105.6 |
| cell 7 | 81.4 | 137.5 | 112.8 | cell 7 | 90.6 | 63.6 | 93.8 |
| cell 8 | 108.3 | 160.0 | 116.7 | cell 8 | 122.7 | 60.0 | 72.7 |
| cell 9 | 94.4 | 85.7 | 91.1 | cell 9 | 21.4 | 100.0 | 142.9 |
| cell 10 | 109.7 | 140.0 | 88.9 | cell 10 | 80.0 | 200.0 | 200.0 |
| cell 11 | 104.7 | 96.6 | 69.4 | cell 11 | 112.5 | 157.1 | 104.2 |
| cell 12 | 85.7 | 133.3 | 171.4 | cell 12 | 50.0 | 100.0 | 100.0 |
| cell 13 | 90.8 | 186.7 | 122.4 | cell 13 | 132.9 | 153.3 | 182.9 |
| cell 14 | 160.0 | 233.3 | 186.7 | cell 14 | 143.8 | 28.6 | 87.5 |
| cell 15 | 71.4 | 220.0 | 107.1 | cell 15 | 104.5 | 133.3 | 59.1 |
| cell 16 | 105.0 | 166.7 | 100.0 | cell 16 | 150.0 | 100.0 | 233.3 |
| cell 17 | 105.4 | 150.0 | 167.9 | cell 17 | 76.9 | 114.3 | 84.6 |
| cell 18 | 117.7 | 127.3 | 135.5 | cell 18 | 88.5 | 66.7 | 69.2 |
| cell 19 | 82.9 | 100.0 | 96.6 | cell 19 | 181.8 | 66.7 | 63.6 |
| cell 20 | 142.1 | 200.0 | 84.2 | cell 20 | 90.4 | 66.7 | 80.8 |
| cell 21 | 33.3 | 300.0 | 50.0 | cell 21 | 250.0 | 100.0 | 200.0 |
| cell 22 | 92.9 | 85.7 | 119.0 | cell 22 | 50.0 | 75.0 | 114.3 |
